# Easily Available, Amphiphilic Diiron Cyclopentadienyl Complexes Exhibit in Vitro Anticancer Activity in 2D and 3D Human Cancer Cells through Redox Modulation Triggered by CO Release

**DOI:** 10.1002/chem.202101048

**Published:** 2021-06-09

**Authors:** Lorenzo Biancalana, Michele De Franco, Gianluca Ciancaleoni, Stefano Zacchini, Guido Pampaloni, Valentina Gandin, Fabio Marchetti

**Affiliations:** ^1^ Department of Chemistry and Industrial Chemistry University of Pisa Via G. Moruzzi 13 I-56124 Pisa Italy; ^2^ Department of Pharmaceutical and Pharmacological Sciences University of Padova Via F. Marzolo 5 I-35131 Padova Italy; ^3^ Department of Industrial Chemistry “Toso Montanari” University of Bologna Viale Risorgimento 4 I-40136 Bologna Italy

**Keywords:** bioinorganic chemistry, CO release; cytotoxicity, diiron complexes, 3D cancer cell models

## Abstract

A straightforward two‐step procedure via single CO removal allows the conversion of commercial [Fe_2_Cp_2_(CO)_4_] into a range of amphiphilic and robust ionic complexes based on a hybrid aminocarbyne/iminium ligand, [Fe_2_Cp_2_(CO)_3_{CN(R)(R’)}]X (R, R’=alkyl or aryl; X=CF_3_SO_3_ or BF_4_), on up to multigram scales. Their physicochemical properties can be modulated by an appropriate choice of N‐substituents and counteranion. Tested against a panel of human cancer cell lines, the complexes were shown to possess promising antiproliferative activity and to circumvent multidrug resistance. Interestingly, most derivatives also retained a significant cytotoxic activity against human cancer 3D cell cultures. Among them, the complex with R=4‐C_6_H_4_OMe and R’=Me emerged as the best performer of the series, being on average about six times more active against cancer cells than a noncancerous cell line, and displayed IC_50_ values comparable to those of cisplatin in 3D cell cultures. Mechanistic studies revealed the ability of the complexes to release carbon monoxide and to act as oxidative stress inducers in cancer cells.

## Graphical abstract

**A class of cationic diiron complexes** containing a variable bridging iminium ligand displays promising antiproliferative activity towards a panel of 2D and 3D cancer cell lines, and a selectivity not correlated to a different degree of uptake in cancerous and noncancerous cells. The mode of action appears to be mainly related to the interference with cell redox processes (ROS production, inhibition of TrxR), initiated by intracellular CO release.

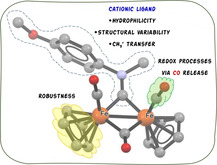

## Introduction

Transition metal complexes have unique characteristics which constitute an indispensable potential in medicinal chemistry, such as the availability of adjacent oxidation states of the metal enabling redox reactions, the reactivity of the coordinated organic fragments addressed by the metal centre, and a broad range of possible geometries, stereochemical configurations and kinetic behaviours.^[1]^ In the last century, the serendipitous discovery of the cytotoxic properties of cisplatin preceded the introduction of few platinum complexes in clinical treatments against various types of cancer.^[2]^ Then, the severe side effects caused by limited selectivity, platinum toxicity and resistance issues^[3]^ have fuelled an intense research for alternative drugs based on other transition metals.^[4]^ In principle, iron is a convenient choice, since it is a bioavailable element and nontoxic in many forms;^[5]^ moreover, its earth abundancy has made several cost‐effective compounds available to the researchers for the exploration of the reactivity and the consequent development of new structural motifs. A variety of mono‐iron compounds has been investigated for the anticancer potential,[^6^, ^7^] and substituted ferrocenes (ferrocifens) have been regarded as promising drug candidates.^[8]^ The feasible Fe^+II^ to Fe^+III^ oxidation in the physiological environment and the conjugation of suitable organic moieties to the robust *sandwich* scaffold are responsible for a considerable activity, which is basically related to the production of damaging metabolites within cancer cells.^[9]^

**Figure 1:**
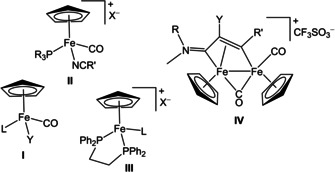
Structures of cyclopentadienyl iron complexes that show anticancer activity (plus year of publication). **I**) L=PPh_2_(alkyl), Y=C(O)Me (2020);^[10a]^ L=CO, Y=Cl, Br, I, NCS, SCN (2016);^[10b]^ L=PPh_3_ or related ring‐substituted molecule, Y=I (2017).^[10c]^ **II**) R=Ph, 4‐C_6_H_4_CO_2_H or 4‐C_6_H_4_F, R′=4‐C_6_H_4_NH_2_, X=PF_6_ (2017);^[10c]^ R=Ph, R′=aryl or vinyl, X=CF_3_SO_3_ (2020).^[11c]^ **III**) L=imidazole or N‐substituted imidazole, X=CF_3_SO_3_ (2013);^[11d]^ L=carbohydrate‐substituted nitrile, X=PF_6_ (2015);^[11a]^ L=N‐heteroaromatic nitrile, X=PF_6_ or CF_3_SO_3_ (2014).^[11b]^ **IV**) R=Me, CH_2_Ph or 2,6‐C_6_H_3_Me_2_ (Xyl), R′=alkyl, aryl, pyridyl, or thiophenyl, Y=H, Ph or S/Se group (2019, 2020).[^17a^, ^17b^]

Following the findings on ferrocifens, some *half‐sandwich* compounds, both neutral‐charged (Figure 1, structure **I**)^[10]^ and cationic (structures **II**–**III**),[^10c^, ^11^] were recently assessed for their cytotoxicity against various cancer cell lines, which might be enhanced by the synergic effect provided by the release of the carbon monoxide co‐ligands.^[12]^ A critical aspect is that, in general, ferrocifens and other organo‐iron compounds possess a poor solubility in physiological environments,^[13]^ resulting in serious drawbacks in the view of clinical applications.^[14]^ In this overall scenario, the medicinal potential of diiron bis‐cyclopentadienyl compounds was unexplored until 2019. The commercial dimer [Fe_2_Cp_2_(CO)_4_] (Cp=η^5^‐C_5_H_5_) represents an inexpensive and convenient entry into the chemistry of diiron compounds, and a plethora of organometallic motifs has been constructed on a bridging coordination site, exploiting the cooperativity of the two metal centres.^[15]^ Some of us reported a preliminary evaluation of the anticancer activity of complexes based on the {Fe_2_Cp_2_(CO)_*x*_} core (*x*=2 or 3),^[16]^ and especially those ones with a bridging vinyliminium ligand (Figure 1, structure **IV**) displayed interesting preliminary cytotoxicity profiles.^[17]^

Here, we show that a general and straightforward two‐step synthetic procedure (reproducible in multigram scales) allows to access a family of compounds showing a promising antiproliferative activity against 2D and 3D human cancer cell systems. Remarkably, 3D cell culture studies constitute an excellent base for more advanced in vivo cancer research, surpassing the traditional assays on monolayer cultures;^[18]^ indeed, 2D models provide limited predictivity for drug testing, while 3D models promise to bridge the gap between traditional 2D cell cultures and in vivo animal models.^[19]^

To the best of our knowledge, the present 3D investigation is one of the first ones ever reported on iron compounds. The proposed diiron complexes combine within a cationic structure two Cp rings, three carbonyls and a variable bridging aminocarbyne ligand, the latter tuning the water solubility, the lipophilicity and the activity. Several experiments aimed to clarify the mechanism of action of the complexes will be discussed.

## Results and Discussion

### Synthesis and characterization of diiron complexes and structural studies

The known complexes **1A**–**H** were prepared by the thermal substitution reaction of the corresponding isocyanides with a slight molar excess of [Fe_2_Cp_2_(CO)_4_] in acetonitrile.^[20]^ The final reaction mixtures were dried, and the crude residues were dissolved in dichloromethane or acetonitrile and treated with a range of alkylating agents under optimized conditions, to afford the corresponding products [**2**–**5**]^+^ (Table 1). The products were purified by alumina chromatography (see the Experimental Sections for details), and finally isolated in good to quantitative yields in a 300 mg to 10 g scale. Deliquescent [**2B**]CF_3_SO_3_ was converted into the less moisture‐sensitive tetrafluoroborate salt by repeated treatment with NaBF_4_ in water followed by CH_2_Cl_2_ extraction. A minor amount of [**2C**]Cl was isolated from the chromatography of [**2C**]CF_3_SO_3_, as a result of triflate/chloride exchange during the chromatographic purification, due to chloride impurities in commercial alumina; then, [**2C**]Cl was reliably obtained from [**2C**]CF_3_SO_3_ using a cation exchange resin. In order to ensure the solid‐state stability of the compounds, the light‐sensitive anions of [**3F**]I and [**5H**]Br were exchanged by metathesis with AgCF_3_SO_3_ in MeCN. In summary, Table 1 shows a general approach to obtain easily accessible ionic diiron complexes with variable substituents on the bridging hydrocarbyl ligand and counter anions, based on the choice of the isocyanide and the alkylating agent, thus offering wide opportunity for tuning the physicochemical properties (Scheme S1 in the Supporting Information). The cations [**2A**–**C**]^+^, [**4G**]^+^ and [**5H**]^+^ are unprecedented in the literature, while the others were previously isolated as [**2D**–**F**]CF_3_SO_3_,[^21^, ^22^] [**3F**]I^[23]^ and [**3G**]CF_3_SO_3_;^[20]^ however, the salts [**3G**]I and [**3F**]CF_3_SO_3_ are novel and an optimized synthesis for [**3F**]I is supplied here. The incorporation of the indolyl moiety in [**2A**]CF_3_SO_3_ is of some relevance, because many indole derivatives have been reported as potent anticancer agents, and some of them have been approved by FDA for clinical treatment.^[24]^ On the other hand, the introduction of the highly polar diethylphosphonato group in [**2B**]BF_4_ is functional to enhance the water solubility.^[25]^

**Table 1:** Two‐step preparation of cationic diiron bis‐cyclopentadienyl complexes with bridging aminocarbyne ligands. Experimental conditions for the second step: [**2A**–**F**]^+^, CH_2_Cl_2_, room temperature; [**3F**]^+^, MeCN, RT; [**3G**]^+^ and [**5H**]^+^, MeCN, reflux; [**4G**]^+^, MeCN, 0 °C. *Not isolated.

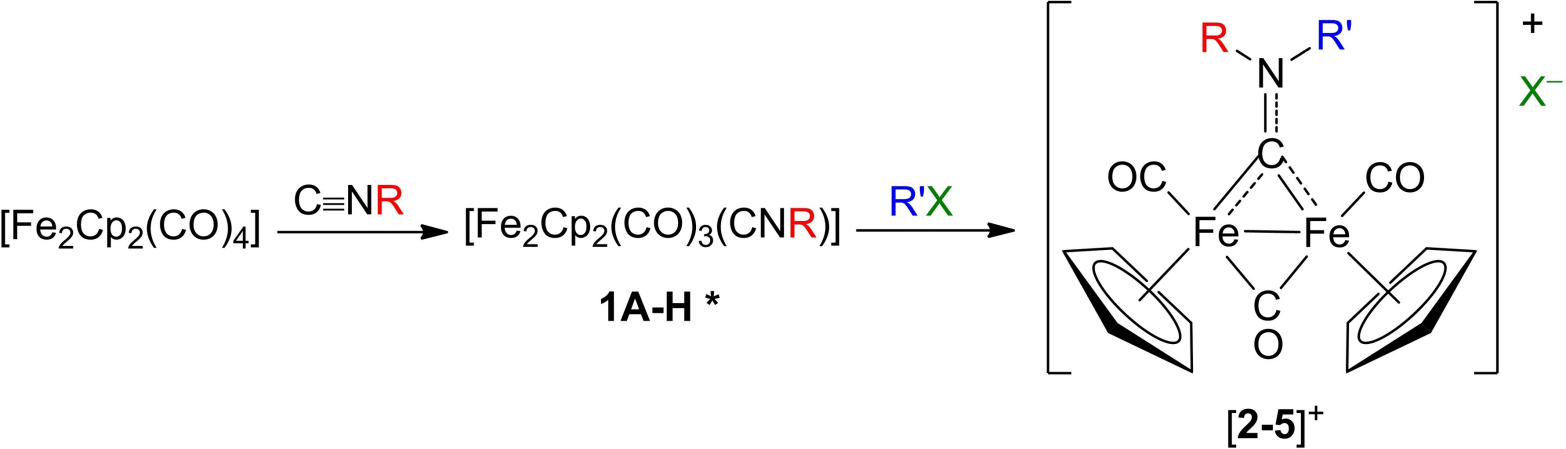			
R		R′X	
1*H*‐indol‐6‐yl	**1**	CH_3_SO_3_CF_3_	[**2A**]^+^
CH_2_P(O)(OEt)_2_	**1B**	CH_3_SO_3_CF_3_	[**2B**]^+^
Cy=C_6_H_11_	**1C**	CH_3_SO_3_CF_3_	[**2C**]^+^
4‐C_6_H_4_OMe	**1D**	CH_3_SO_3_CF_3_	[**2D**]^+^
Xyl=2,6‐C_6_H_3_Me_2_	**1E**	CH_3_SO_3_CF_3_	[**2E**]^+^
Me	**1F 1F**	CH_3_SO_3_CF_3_	[**2F**]^+^
		CH_2_=CHCH_2_I	[**3F**]^+^
2‐naphthyl	**1G 1G**	CH_3_I	[**3G**]^+^
		[Et_3_O]BF_4_	[**4G**]^+^
CH_2_Ph	**1H**	PhCH_2_Br	[**5H**]^+^

**Figure 2:**
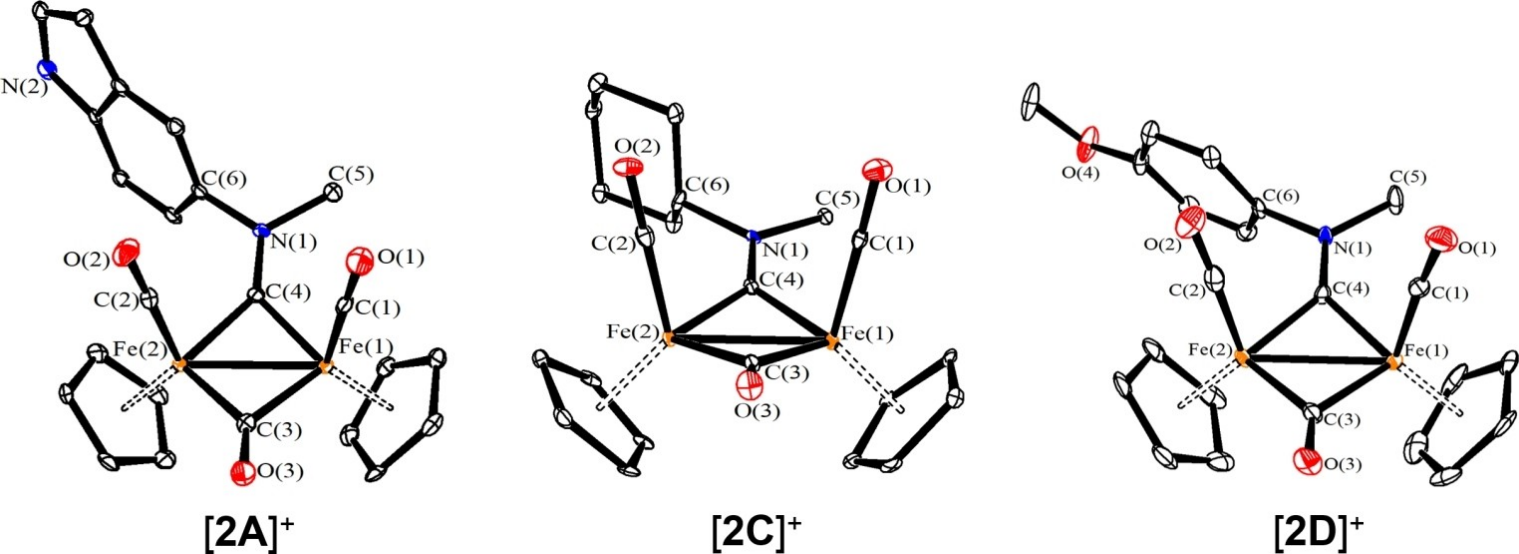
Molecular structures of [**2A**]^+^, [**2C**]^+^ and [**2D**]^+^ (triflate salts). Displacement ellipsoids are at the 30 % probability level. H‐atoms have been omitted for clarity.

New compounds were fully characterized by elemental analysis, IR and multinuclear NMR spectroscopy. In addition, the structures of [**2A**]CF_3_SO_3_, [**2C**]CF_3_SO_3_, [**2C**]Cl and [**2D**]CF_3_SO_3_ were confirmed by single crystal X‐ray diffraction. Relevant bonding parameters are comparatively compiled in Table 2, and a view of the cations are shown in Figure 2. The structure of the cations consists of a typical *cis*‐{Fe_2_Cp_2_(CO)_2_} core, connected to a further CO and an aminocarbyne group occupying the two bridging sites. Along this series of complexes, the C(4)‐N(1) distance does not significantly vary, indicating a substantial double‐bond character which accounts for the complementary description of the bridging ligand as iminium (see below).

**Table 2:** Selected bond lengths [Å] and angles [°] for [**2A**]^+^, [**2C**]^+^, and [**2D**]^+^.

	**[2A]^+^**	**[2C]^+ (a)^**	**[**2C**]^+ (b)^**	**[2D]^+^**
Fe(1)‐Fe(2)	2.5136(11)	2.509(3)	2.5152(15)	2.5161(10)
Fe(1)‐C(1)	1.760(6)	1.765(16)	1.776(7)	1.774(6)
Fe(2)‐C(2)	1.782(6)	1.760(16)	1.754(8)	1.762(6)
Fe(1)‐C(3)	1.954(6)	1.924(16)	1.915(8)	1.935(5)
Fe(2)‐C(3)	1.944(6)	1.965(16)	1.962(7)	1.934(5)
Fe(1)‐C(4)	1.874(6)	1.881(14)	1.883(8)	1.870(5)
Fe(2)‐C(4)	1.870(6)	1.873(15)	1.888(8)	1.866(5)
C(1)‐O(1)	1.137(7)	1.119(17)	1.139(9)	1.139(6)
C(2)‐O(2)	1.131(7)	1.119(17)	1.147(10)	1.148(7)
C(3)‐O(3)	1.155(7)	1.150(18)	1.172(9)	1.161(6)
C(4)‐N(1)	1.289(7)	1.291(18)	1.274(10)	1.287(6)
Fe(1)‐C(3)‐Fe(2)	80.3(2)	80.3(6)	80.9(3)	81.1(2)
Fe(1)‐C(4)‐Fe(2)	84.3(2)	83.9(6)	83.7(3)	84.7(2)
Fe(1)‐C(1)‐O(1)	177.9(6)	178.9(14)	178.5(7)	175.6(5)
Fe(1)‐C(2)‐O(2)	177.8(6)	179.6(15)	179.5(8)	176.3(5)
C(4)‐N(1)‐C(5)	122.2(5)	122.6(12)	122.5(7)	123.4(4)
C(4)‐N(1)‐C(6)	124.1(5)	121.0(12)	121.4(6)	120.9(4)
C(5)‐N(1)‐C(6)	113.7(4)	116.3(11)	116.0(6)	115.5(4)

(a) As [**2C**]CF_3_SO_3_. (b) As [**2C**]Cl.

**Figure 3:**
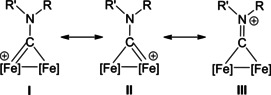
Resonance structures for the bridging hydrocarbyl ligand in the diiron complexes [**2**–**5**]^+^ and **6**. Structures **I** and **II**: aminocarbyne; structure **III**: iminium.

The bonding description of {μ‐CNR(R′)} ligands in dimetal complexes is not clearly defined in the literature, therefore we performed a DFT study to shed light on this point. The representative structure of [**2C**]^+^ was calculated and compared to that of the parent compound *cis*‐**1C** with a bridging cyclohexyl‐isocyanide ligand (CNCy). DFT‐optimized structures and relevant bonding parameters are reported in Table S1 and Figure S1. In **1C**, the (μ‐C)−N Mayer bond order (b.o.)^[26]^ is 2.0 and the bond distance is 1.233 Å, in agreement with the X‐ray data available for related systems [(μ‐C)−N=1.26(3) Å for CN*i*Pr instead of CNCy].^[27]^ The b.o. of (μ‐C)−N lowers to 1.58 upon methylation of the isocyanide moiety leading to [**2 C**]^+^. The nature of the nitrogen substituents has a negligible influence, and for instance the corresponding b.o. is 1.54 in the case of [**2A**]^+^, the related bond length being consistent with the crystallographic characterization of [**2A**]CF_3_SO_3_ [1.300 vs. 1.289(7) Å; Table 2]. These theoretical results strongly support the description of the bridging N‐containing ligand in the cationic complexes [**2**–**5**]^+^ as an almost perfect aminocarbyne‐iminium hybrid structure (Figure 3).

Then, in order to clarify the possible effect of the net ionic charge of the complex, we also synthesized and characterized the novel neutral derivative [Fe_2_Cp_2_Cl(CO)(μ‐CO){μ‐CNMe(Cy)}], **6** (see Experimental Section). Based on a comparison of the ^1^H NMR and IR spectra with data available in the literature for similar compounds,^[28]^ **6** exists in CDCl_3_ solution as a nearly equimolar mixture of two isomers, maintaining the *cis*‐geometry of the Cp ligands (as ascertained by ^1^H NOE experiments, Figure S32) and differing in the orientation of the *N*‐substituents with respect to the chloride (*E*/*Z* isomers). This feature suggests that the (μ‐C)−N bond in the neutral **6** still holds a substantial double bond character. The X‐ray structure of the *E* isomer was determined and is shown in Figure S2, although the low quality of the crystals prevents a detailed discussion of bonding parameters.

In agreement with the NMR evidence, DFT outcomes indicate that **6^Z^** (*Z* isomer) and **6^E^** (*E* isomer) are practically isoenergetic, the latter being only 0.2 kcal mol^−1^ more stable than the former (Figure S1). In both *E/Z* isomers, the bridging {CN(Me)(Cy)} group resembles that in [**2C**]^+^, with only a slight decrease of the (μ‐C)−N bond order (from 1.58 to 1.52). It is interesting to note that the bond length of the bridging carbonyl in **6** is very close to that in **1C** (1.187 vs. 1.188 Å), highlighting that the isocyanide/CO and aminocarbyne/Cl combinations supply very similar electron densities for back‐donation to the diiron framework.^[29]^

Salient spectroscopic features of all complexes are summarized in Table S2, and NMR and IR spectra are supplied in Figures S3–S32. In the IR spectra (CH_2_Cl_2_ solution), the vibrations due to the terminal carbonyl ligands occur in the ranges 2018–2028 (anti‐symmetric stretching) and 1985–1996 cm^−1^ (symmetric stretching), while the band related to the bridging carbonyl is found at 1833–1853 cm^−1^. Moreover, the absorption attributed to the carbyne‐N bond falls in between 1530 to 1602 cm^−1^, the higher values being associated to the N‐substituents with higher electron‐donor ability. The ^1^H and ^13^C NMR spectra (recorded in CDCl_3_, CD_3_CN or [D_6_]DMSO) display one set of resonances, ascribable to a single isomeric form featuring the Cp ligands in mutual *cis* position, as found in the X‐ray structures and confirmed by ^1^H NOE experiments in the case of [**4G**]^+^ (Figure S27). Two resonances for the Cp and terminal CO ligands are distinguishable in the unsymmetrical complexes, due to hampered rotation around the partially double C−N bond (see above); accordingly, such resonances coincide in the complexes [**2F**]^+^ and [**5H**]^+^, displaying two identical N groups. In the ^13^C NMR spectra, the bridging {CN} unit is detected at low fields (i. e., around 320 ppm), as it is typical for bridging aminocarbynes.[^20^, ^30^] Compound [**2B**]^+^ features a ^31^P NMR resonance at 17 ppm for the phosphonato group, and the ^13^C signal for the carbyne appears as a doublet (^3^ *J*_CP_ ca. 6 Hz).

Small differences between the spectroscopic data of [**2C**]CF_3_SO_3_ and [**2C**]Cl in chlorinated solvents are ascribable to the presence of ion pairs (or higher aggregates);^[31]^ otherwise, the two compounds display superimposable ^1^H NMR spectra in protic polar solvents (D_2_O and CD_3_OD), where ion pairs are practically absent.^[32]^

### Solubility, partition coefficient, stability in aqueous media and interaction with a model protein

A detailed study on the behaviour of a selection of the diiron complexes in aqueous media was undertaken. Experimental procedures are provided in the Experimental Section and in the Supporting Information, with data collected in Tables 3, S3 and S4. The D_2_O solubility of the compounds was assessed by ^1^H NMR, ranging from sub‐millimolar to 0.15 M in the case of [**2C**]Cl. The octanol/water partition coefficients (log *P*_ow_) were measured by a UV/Vis method and fall in between 0 and −1, reflecting an amphiphilic or slightly hydrophilic character. An exception is represented by [**2B**]BF_4_, wherein the marked hydrophilicity imparted by the diethylphosphonato does not appear sufficiently balanced (log *P*_ow_<−1.5). Conversely, those complexes with aromatic groups (i. e., 1*H*‐indol‐6‐yl, 2‐naphthyl and bis‐benzyl in [**2A**]^+^, [**3G**]^+^, [**4G**]^+^ and [**5H**]^+^) are less soluble in water and display increased affinity for *n*‐octanol (log *P*_ow_≈0). The diiron compounds are inert in aqueous solution at room temperature, and undergo a slow degradation at 37 °C. Under such conditions, a variable amount (50 to 86 %; Table S3) of the starting material was detected by ^1^H NMR in D_2_O or D_2_O/CD_3_OD mixtures after 72 h.^[33]^ During this time, the precipitation of a brown solid was observed, which in one case (from a [**2C**]CF_3_SO_3_ solution) was separated and identified as γ‐Fe_2_O_3_ by Raman analysis. In addition, headspace GC analyses performed on 5 % MeOH aqueous solutions of the compounds maintained at 37 °C allowed the identification and quantitation of the carbon monoxide released over a 48 h period (Table S4). These facts indicate that the thermal degradation process in aqueous solution leads to the progressive disassembly of the organometallic scaffold.^[16]^ The presumed, controlled release of the iron centres (converted to iron oxide, see above) and carbon monoxide are two aspects that deserve attention with reference to the biological activity: note that the decay of ferrocene drug candidates into Fe^II^ ions was previously associated to their cytotoxicity,^[34]^ while the activity of various iron carbonyl complexes was correlated to CO dissociation in the physiological media.^[35]^

**Table 3:** Solubility in D_2_O (21 °C) and octanol/water partition coefficient (log *P*_ow_) for diiron compounds.

Compound	Solubility [mol L^−1^]	Log *P*_ow_
[**2A**]CF_3_SO_3_	ca. 3 ⋅ 10^−4^ M	0.0±0.1
[**2B**]BF_4_	5.2 ⋅ 10^−2^ M	≤ −1.5
[**2C**]CF_3_SO_3_	6.2 ⋅ 10^−3^ M	−0.46±0.02
[**2C**]Cl	1.5 ⋅ 10^−1^ M	−1.1±0.08
[**2D**]CF_3_SO_3_	4.1 ⋅ 10^−3^ M	−0.25±0.04
[**2E**]CF_3_SO_3_^[b]^	1.4 ⋅ 10^−3^ M	−0.27±0.04
[**2F**]CF_3_SO_3_^[b]^	3.2 ⋅ 10^−3^ M	−0.9±0.1
[**3F**]CF_3_SO_3_	3.4 ⋅ 10^−3^ M	−1.01±0.02
[**3G**]CF_3_SO_3_^[b]^	not soluble ^[a]^	0.29±0.03
[**4G**]BF_4_	not soluble ^[a]^	0.1±0.1
[**5H**]CF_3_SO_3_	6 ⋅ 10^−4^ M	0.2±0.02

[a] Solubility below the lowest value of quantitation (ca. 3×10^−4^ M). [b] Data taken from ref. 16a.

Next, the stability of the diiron compounds in a cell culture medium (DMEM) solution at 37 °C, over a shorter time range (24 h), was assessed by ^1^H NMR. Notably, a considerable fraction (70–88 %; Table S3) of the starting material was detected at the end of the experiment, with a limited formation of an orange‐brown precipitate analogous to that described above.

To gain insights into the reactivity of the diiron compounds with possible biomolecular targets, we selected lysozyme, a small model protein often used to test interactions with metal compounds.^[36]^ Following 24 h incubation at 37 °C, diiron compounds/lysozyme solutions (2 : 1 molar ratio) in ammonium acetate were filtered and analysed by ESI‐MS. In all cases, the original organometallic cation was identified as the major Fe‐containing compound in solution (Figures S33‐S40). Concerning the protein scaffold, the only identified adduct from the interaction with [**2**–**3**]^+^ was the methylated derivative, which was also checked by HPLC separation (Figure S41). The expected diiron counterparts, that is, **1A**–**H**, could not be detected possibly due to their water insolubility. The [lysozyme+Me^+^] to [lysozyme] ratios, calculated on extracted ion chromatograms after protein peak reconstruction, are reported in Table 4. Compounds lacking *N*‐methyl groups (i. e., [**4G**]^+^ and [**5H**]^+^) did not react with lysozyme. Methylation is a common post‐translational modification of protein/enzymes (methyltransferase enzymes) but it has also been observed as a result of the interaction of proteins with various electrophiles.^[37]^

**Table 4:** Methylation ratio (%) upon lysozyme interaction with the diiron compounds (1 : 2 molar ratio, 24 h, 37 °C), as determined by ESI‐MS measurements.

Compound	% Lysozyme methylation (alkylation) ^[a]^	Compound	% Lysozyme methylation (alkylation) ^[a]^
[**2A**]CF_3_SO_3_	45 %	[**2F**]CF_3_SO_3_	35 %
[**2B**]BF_4_	49 %	[**3F**]CF_3_SO_3_	47 %
[**2C**]CF_3_SO_3_	50 %	[**4G**]BF_4_	0 %^[b]^
[**2D**]CF_3_SO_3_	31 %	[**5H**]CF_3_SO_3_	0 %^[b]^
[**2E**]CF_3_SO_3_	59 %	Blank exp	0 %^[b]^

[a] Calculated by the relative peak integrals for flow injection MS analysis. [b] No other protein MS peak beside that of lysozyme was observed.

### Cytotoxic activity in 2D cell cultures

The diiron complexes [**2A**]^+^, [**2C**–**E**]^+^, [**3F**–**G**]^+^, [**5H**]^+^ (as CF_3_SO_3_ salts) and [**2B**]^+^, [**4G**]^+^ (as BF_4_ salts) were tested for their cytotoxic potential by means of the MTT assay, as reported in the Experimental Section. The most convenient counter anion in terms of preparation and handling/storage was chosen in each case; in particular, [**2C**]CF_3_SO_3_ was included in the study, while [**2C**]Cl was excluded, being highly moisture sensitive and thus affecting the accuracy of the weighting operation. The in‐house human cancer cell line panel contains examples of ovarian (2008), colon (HCT‐15), pancreatic (PSN‐1), and breast (MCF‐7) cancers as well as of melanoma (A375). Cisplatin was used as a reference and tested under the same experimental conditions. The cytotoxicity parameters, expressed in terms of IC_50_ obtained after 72 h of exposure, are listed in Table 5. In addition, the cytotoxicity was also evaluated for shorter time of exposure (24 h) towards PSN‐1 cancer cells (Table S5). Data analysis reveals that the cytotoxic activity showed by tested complexes is time‐ and dose‐dependent. In particular, the highly hydrophilic complexes [**2B**]BF_4_ and [**3F**]CF_3_SO_3_ did not impact cell viability (average IC_50_ values were over 200 or 100 μM for all tested cell lines), whereas the other compounds elicited IC_50_ values in the micromolar range. Despite all cytotoxic complexes showed an average in vitro antitumor potential lower than that of cisplatin, [**2C**]CF_3_SO_3_ and [**2D**]CF_3_SO_3_ proved to be the most effective derivatives, with average IC_50_ values of 17.4 and 16.6 μM (72 h time trials), and showed a similar pattern of response over the five tested cell lines. In particular, [**2D**]CF_3_SO_3_ exhibits a comparable activity with respect to cisplatin against HCT‐15, PSN‐1 and MCF‐7 cells.

**Table 5:** In vitro antitumor activity of diiron complexes in 2D cell cultures. Cells (3–8×10^3^ mL^−1^) were treated for 72 h with increasing concentrations of the tested compounds. The cytotoxicity was assessed by the MTT test. IC_50_ values were calculated by a four‐parameter logistic model 4‐PL (*P*<0.05). In brackets RF=IC_50_ (resistant cells)/IC_50_ (wild‐type cells) for MCF‐7 cells.SI=selectivity index (see main text).

Compound	IC_50_ (μM)±SD							SI
	2008	HCT‐15	A375	PSN‐1	MCF‐7	MCF‐7 ADR	HEK293	
[**2A**]CF_3_SO_3_	55.5±4.8	69.5±6.7	50.8±6.1	54.3±6.2	63.2±5.5	84.5±8.5 (1.3)	135.5±6.4	2.3
[**2B**]BF_4_	>100	>100	>100	>100	>100	>100	>100	−
[**2C**]CF_3_SO_3_	28.4±5.4	9.2±1.8	16.8±2.1	15.3±2.7	17.3±3.3	23.3±4.1 (1.3)	62.5±4.9	3.6
[**2D**]CF_3_SO_3_	21.3±3.1	15.6±3.5	15.5±2.6	16.2±2.9	14.3±2.6	22.6±4.2 (1.6)	94.3±6.1	5.6
[**2E**]CF_3_SO_3_	38.8±4.5	37.3±3.5	34.6±4.0	41.7±6.3	44.7±5.5	66.9±5.2 (1.5)	96.6±2.9	2.4
[**3F**]CF_3_SO_3_	>100	>100	>100	>100	>100	>100	>100	−
[**3G**]CF_3_SO_3_	36.6±3.4	24.6±2.9	25.2±3.3	26.3±2.8	20.7±3.0	30.5±6.7 (1.5)	90.3±5.3	3.3
[**4G**]BF_4_	38.8±6.9	22.9±5.8	26.2±5.6	25.6±4.5	21.4±3.6	26.9±5.1 (1.3)	89.5±4.5	3.3
[**5H**]CF_3_SO_3_	37.4±5.2	32.5±4.8	30.8±5.1	33.7±4.2	36.2±6.1	44.7±6.1 (1.2)	98.3±4.2	2.8
cisplatin	2.2±1.4	16.5±2.2	2.1±0.3	12.1±2.9	8.8±0.2	−	18.1±3.6	2.2
doxorubicin	−	−	−	−	0.2±0.03	7.8±2.2 (39)	−	−

As one of the main drawbacks of chemotherapeutics is the toxic effect toward noncancerous cells, we measured the cytotoxicity of tested complexes against a noncancerous cell line (HEK293) and calculated the selectivity index (SI, defined as the ratio between the IC_50_ value in noncancerous cells and the corresponding average IC_50_ value related to cancer cells; Table 5). The diiron complexes are generally less cytotoxic against HEK293 noncancerous cells, and again [**2D**]CF_3_SO_3_ exhibited the best profile (SI value of 5.6 to be compared with 2.2 for cisplatin). Overall, it appears that slight structural variations on the iminium group may have a notable impact on the activity of the cations, although not obvious: for instance, [**2D**]CF_3_SO_3_ and [**2E**]CF_3_SO_3_ differ from each other in the nature and the position of peripheral arene substituents and display almost identical log *P*_ow_ values, notwithstanding the former is significantly more cytotoxic and selective than the latter.

The antiproliferative activity was also investigated in a multidrug‐resistant cancer cell model overexpressing P‐gp (MCF‐7 ADR cells). All complexes exhibited activity levels very similar on both sensitive (MCF‐7) and multi‐drug resistant (MCF‐7ADR) cell lines; interestingly, the resistance factor (RF) values (Table 5) range from 24 to 35 times lower than that of doxorubicin, a drug belonging to the MDR spectrum, thus indicating the ability of the complexes to overcome MDR phenomena and not to act as P‐gp substrates.

### Cellular uptake in cancerous and noncancerous cells

**Figure 4:**
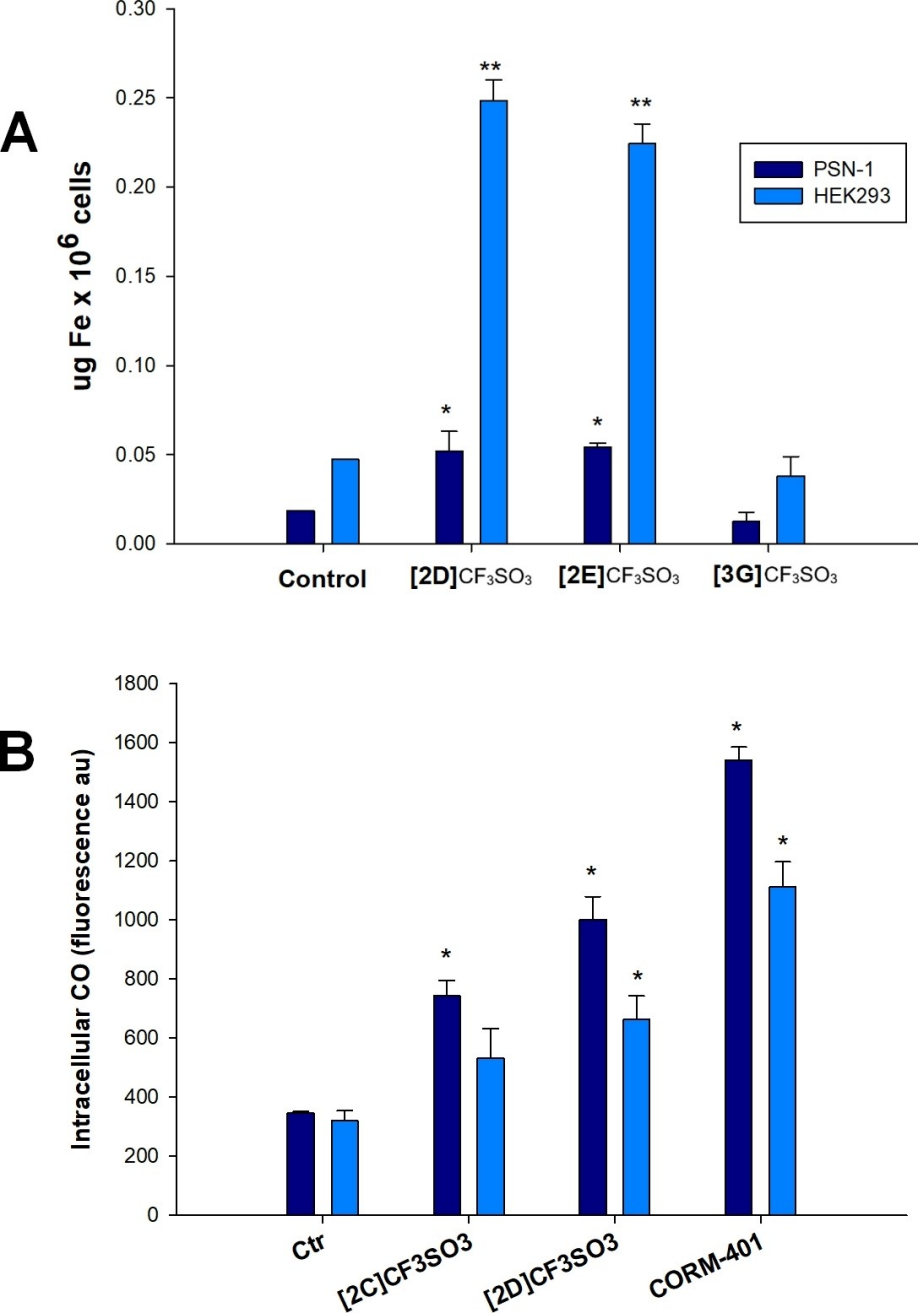
Intracellular detection of iron and carbon monoxide. A) Cellular uptake in PSN‐1 and HEK293 cells; cells were incubated with 50 μM of complexes for 24 h, and the cellular iron content was detected by GFAAS analysis. B) Intracellular CO levels were determined by using a fluorescent probe (BioTracker Carbon Monoxide Probe 1 Live Cell Dye®, Merck, Germany) after 30 min of treatment of PSN‐1 or HEK293 cells with 20 μM [**2C**]CF_3_SO_3_ and [**2D**]CF_3_SO_3_, or 20 μM CORM‐401 as positive control. ** *P*<0.01, * *P*<0.05.

In order to correlate the cytotoxic potential to the ability of the newly developed complexes to enter cells, we performed uptake experiments. To this purpose, we selected the most promising compound [**2D**]CF_3_SO_3_ (log *P*_ow_=−0.25) together with the less active/selective [**2E**]CF_3_SO_3_ (log *P*_ow_=−0.27) and [**3G**]CF_3_SO_3_ (log *P*_ow_=+0.29), to be assessed for their accumulation in human pancreatic PSN‐1 cancer cells and in untransformed HEK293 cells. Thus, cells were treated for 24 h with equimolar concentrations (50 μM) of the tested compounds. The cellular iron levels were quantified by means of GF‐AAS analysis, and the results, expressed as μg Fe per 10^6^ cells, are shown in Figure 4A. Complex [**3G**]CF_3_SO_3_ was ineffective in entering human cells, while both [**2D**]CF_3_SO_3_ and [**2E**]CF_3_SO_3_ were able to induce cellular iron accumulation, and no significant differences in the ability to enter cells were evidenced for the two complexes. Considering the log *P*_ow_ values, the obtained outcomes highlight that the hydrophilic complexes [**2D**]CF_3_SO_3_ and [**2E**]CF_3_SO_3_ are significantly more effective in entering cells compared to the more lipophilic one [**3G**]CF_3_SO_3_. In addition, both [**2D**]CF_3_SO_3_ and [**2E**]CF_3_SO_3_ led to major iron accumulation in untransformed cells with respect to pancreatic cancer cells. Intriguingly, these findings attest that their selectivity towards cancer cells with respect to untransformed ones cannot be attributable to their different ability to cross cellular plasmalemma and to enter human cells, and the reason of their preferential activity against cancer cells might be dependent on their ability to interfere with a cancer specific target.

### Cytotoxic activity in 3D cell cultures

The remarkable cell‐killing effect observed in 2D cultured cells prompted us to evaluate the in vitro antitumor activity of the complexes on 3D cell cultures. Differently from 2D monolayer culture, 3D spheroid cell culture systems comprise cancer cells in various cell growth stages. Consequently, the multicellular cancer spheroid model is recognized to better reflect the tumour mass in vivo regarding drug permeation, cell interactions, gene expression, hypoxia and nutrient gradients with respect to monolayer cell cultures, making 3D models more predictive than conventional 2D monolayer cultures in screening antitumor drugs.^[38]^ Table 6 summarizes the IC_50_ values obtained after treatment of 3D cell spheroids of human ovarian (2008) and pancreatic (PSN‐1) cancer cells with the diiron complexes and cisplatin. In accordance with 2D chemosensitivity assays, compounds [**2B**]CF_3_SO_3_ and [**3F**]CF_3_SO_3_ were ineffective against cancer cells spheroids, whereas [**2D**]CF_3_SO_3_ was the most effective complex among the series, thus indicating its ability to better penetrate the spheroid and reach the hypoxic core. It is noteworthy that [**2D**]CF_3_SO_3_ exhibited a level of activity either comparable (PSN‐1 cells) or even superior (2008 cells) to that of cisplatin.

**Table 6:** Activity of diiron complexes in 3D cell cultures.

	IC_50_±SD (μM)	
**Compound**	2008	PSN‐1
**[2A]CF_3_SO_3_**	>100	>100
**[2B]BF_4_**	>100	>100
**[2C]CF_3_SO_3_**	66.4±8.6	78.2±9.1
**[2D]CF_3_SO_3_**	42.8±4.2	53.7±6.1
**[2E]CF_3_SO_3_**	65.1±6.8	82.3±4.8
**[3F]CF_3_SO_3_**	>100	>100
**[3G]CF_3_SO_3_**	88.5±3.2	>100
**[4G]BF_4_**	58.9±4.8	77.4±8.4
**[5H]CF_3_SO_3_**	73.2±6.2	86.2±6.4
**cisplatin**	57.6±3.6	61.7±12.1

Spheroids (2.5×10^3^ cells/well) were treated for 72 h with increasing concentrations of tested compounds. The growth‐inhibitory effect was evaluated by means of the acid phosphatase (APH) test. IC_50_ values were calculated from the dose‐survival curves using a four‐parameter logistic model (*p*<0.05). SD=standard deviation.

An important aspect of the collected data is that only a limited decrease of activity is observed with the cytotoxic compounds, the IC_50_ ratio related to 3D and 2D experiments ranging from 2 to 5 for both cell lines; in particular, this ratio is approximately 2 for the cytotoxic diiron complexes against the 2008 cell line, while the corresponding value for cisplatin is 26. Note that a substantial drop of antiproliferative activity has been often recognized for potential anticancer metal drugs in 3D cell cultures compared to the corresponding 2D ones, highlighting the need for a combination of both models for screening of compounds.^[1c]^

### Intracellular CO release, ROS production and inhibition of thioredoxin reductase

Additional studies were conducted in order to elucidate the mode of action underlying the cell killing effect of the newly synthesized diiron complexes in cancer cells. In particular, based on the evidence of their tendency to slowly release CO in aqueous media (see section on solubility, partition coefficient, stability in aqueous media), we evaluated the ability of the most performant complexes [**2C**]CF_3_SO_3_ and [**2D**]CF_3_SO_3_ to release carbon monoxide inside PSN‐1 pancreatic cancer cells and HEK293 noncancer cells. A fluorometric test was conducted over a short time interval (30 min) by means of a CO sensing dye (Figure 4B). Interestingly, both complexes were effective in significantly release CO in PSN‐1 cells whereas lower levels of CO were detected in HEK293 cells. These results attest the power of the novel diiron complexes to release CO in cellular milieu and, more importantly, could at least in part explain the tumour‐selective cytotoxic effect exerted by [**2C**]CF_3_SO_3_ and [**2D**]CF_3_SO_3_. Note that the ability of a range of related diiron cyclopentadienyl complexes to release CO ligands in aqueous environments was previously demonstrated.[^16a^, ^39^] The relatively fast release of carbon monoxide within the cell might be associated to the presence of a consistent amount and variety of biological substrates, which can quicken the formation of CO substitution diiron products. As a matter of fact, it is well documented that [**2E**–**F**]CF_3_SO_3_ are susceptible to replacement of one terminal CO ligand by suitable C‐, P‐, S‐ and N‐donors to give adducts of general formula [Fe_2_Cp_2_(CO)(L)(μ‐CO){μ‐CNR(Me)}]^0/+^.[^15b^, ^16a^, ^20^] Although the nature of L in the cell context is far from being clarified, it is reasonable to speculate that CO release is readily operative via such substitution pathway. However, an acceleration of the route leading to the extensive decomposition of the diiron scaffold (elimination of three COs per complex), as observed in aqueous media, might also contribute.

**Figure 5:**
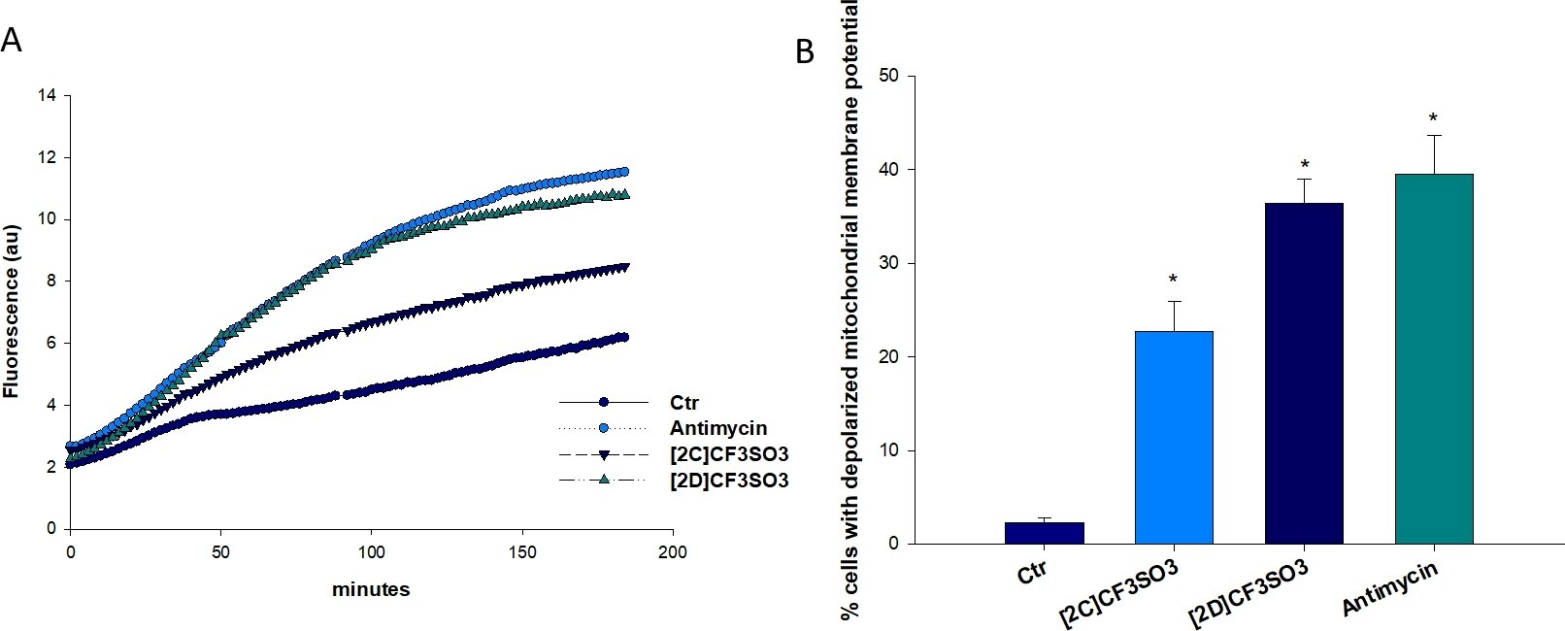
Effects induced by compounds [**2C**]CF_3_SO_3_ and [**2D**]CF_3_SO_3_ at the mitochondrial level. A) ROS production in PSN‐1 cells. Cells were pre‐incubated in phosphate‐buffered saline (PBS)/10 mM glucose medium for 20 min at 37 °C in the presence of 10 mM CM‐H_2_DCFDA and then treated with increasing concentrations of [**2C**]CF_3_SO_3_ and [**2D**]CF_3_SO_3_ or antimycin (3 μM). The fluorescence of DCF was measured at *λ*_ex_=485 nm and *λ*_em_=527 nm. B) Effects of [**2C**]CF_3_SO_3_ and [**2D**]CF_3_SO_3_ on mitochondrial membrane potential. PSN‐1 cells were treated for 24 h with increasing concentrations of [**2C**]CF_3_SO_3_ and [**2D**]CF_3_SO_3_ or antimycin (3 μM) and stained with TMRM (10 nM). Fluorescence was estimated at *λ*_ex_=490 nm and *λ*_em_=590 nm. Data are the means of five independent experiments. Error bars indicate S.D. * *P*<0.05.

Iron is a redox active metal that can generate ROS in cells via the Fenton reaction, thus inducing cellular damage and ultimately leading to cell death.^[40]^ Coherently, several iron complexes have been shown to exert antitumor activity through redox stress induction (see the Introduction).^[8]^ Based on this premises, we evaluated the effects induced by the diiron complexes on the mitochondria activity and the cellular redox environment. More precisely, we investigated the ROS production and the alteration of the mitochondrial membrane potential. Treatment of PSN‐1 cells with [**2C**]CF_3_SO_3_ and [**2D**]CF_3_SO_3_ determined a substantial increase in cellular ROS production, in a time‐dependent manner (Figure 5A). Remarkably, after 2 h treatment with [**2D**]CF_3_SO_3_, the hydrogen peroxide content was similar to that induced by antimycin, a classical inhibitor of the mitochondrial respiratory chain at the level of complex III. Consistently, a significant dose‐dependent increase of cells with depolarized mitochondria was observed after 24 h‐treatment of PSN‐1 cells with [**2C**]CF_3_SO_3_ and [**2D**]CF_3_SO_3_ (Figure 5B). The time‐dependent ROS basal production is probably associated to more than one phenomenon. First of all, the progressive disruption of the diiron structure leads to the extrusion of the Fe^I^ centres, which are expected to subsequently convert into Fe^III^ (in fact, slow precipitation of iron(III) oxides has been recognized from water solution, see above). Otherwise, based on previous voltammetric measurements on [**2E**–**F**]CF_3_SO_3_,^[41]^ oxidation of iron enclosed in the complex is out of the range of biologically relevant potentials. In the cases of [**2**–**3**]^+^, some contribution to ROS might be provided by the partial generation of the neutral products **1A**–**H** upon CH_3_^+^ elimination (see above); in principle, the absence of a net positive charge in **1A**–**H** might favour the oxidation of these complexes or their possible derivatives in the cells.^[40]^

**Figure 6:**
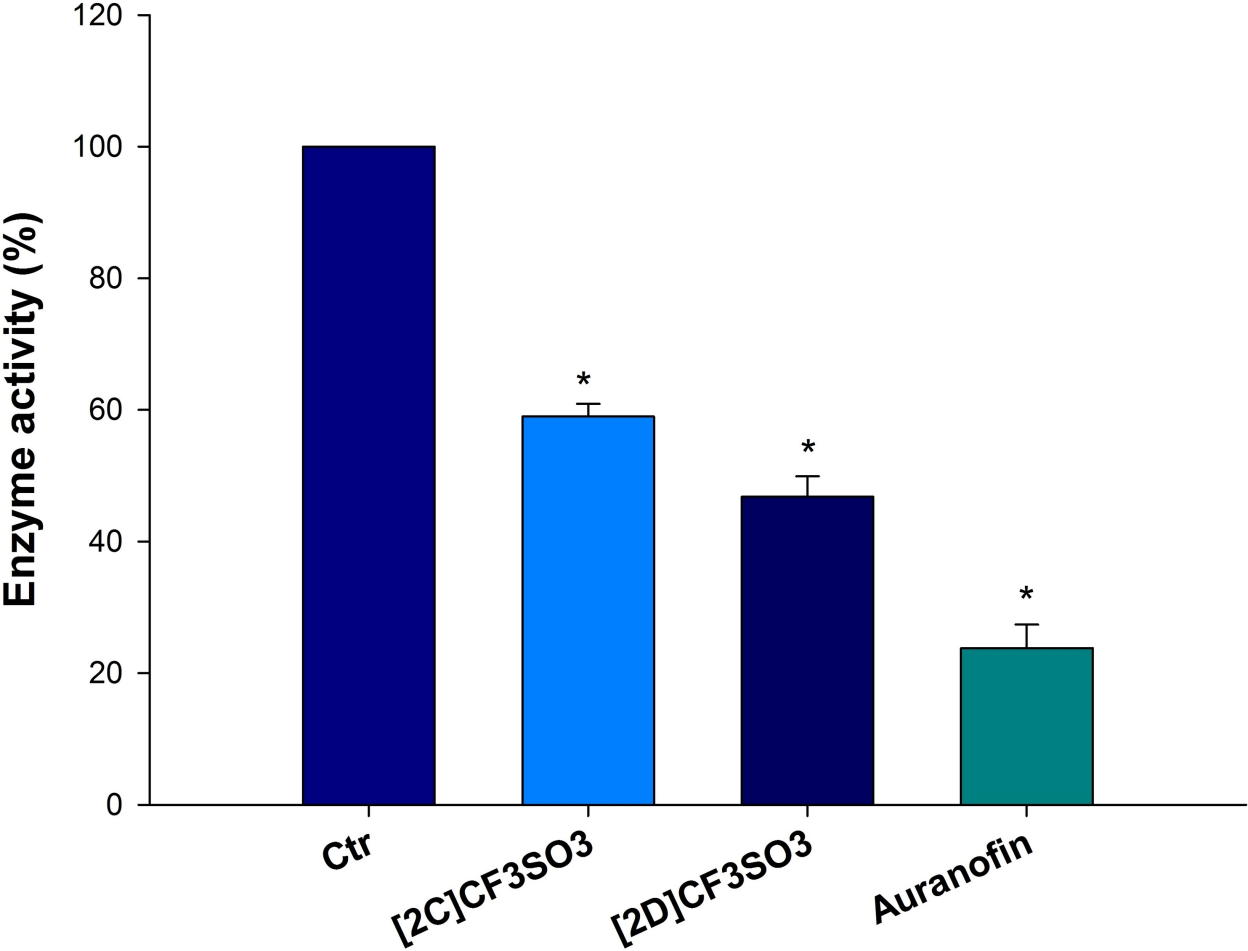
Effects of compounds [**2C**]CF_3_SO_3_, [**2D**]CF_3_SO_3_ and Auranofin as positive control on TrxR in human pancreatic cells. PSN‐1 cells were incubated for 24 h with the IC_50_ of tested compounds. Afterwards, cells were washed twice with PBS and lysed. TrxR activity was tested by measuring the NADPH‐dependent reduction of DTNB. * *P*<0.05.

It has recently emerged that the redox stress induced by some iron complexes might also be attributed to their ability to inhibit the selenoenzyme thioredoxin reductase (TrxR). In particular, Rigobello and co‐workers reported the capacity of a ferrocenyl diphenol and of a Tamoxifen‐like ferrocifen to target and hamper TrxR activity.^[42]^ On this basis, we thought of interest to test the ability of the most promising diiron complexes [**2C**]CF_3_SO_3_ and [**2D**]CF_3_SO_3_ to target TrxR both in cell‐free experiments and in cells. The two complexes showed a similar pattern of response, being completely ineffective in hampering TrxR in cell‐free experiment (data not shown) but elicited a substantial inhibition of the selenoenzyme in human pancreatic PSN‐1 cancer cells (Figure 6). In particular, PSN‐1 cells treated with 25 μM of [**2C**]CF_3_SO_3_ and [**2D**]CF_3_SO_3_ for 24 h showed a residual TrxR cellular activity of 57 and 39 %, respectively. These data well correlate with the observed formation of carbon monoxide in cells, and suggest that the inhibition of TrxR is ascribable to the interaction with diiron complexes and organic fragments generated from [**2**–**5**]^+^ by dissociation of CO ligand(s). On the other hand, TrxR inhibition might also be consistent with the capacity of the complexes to methylate specific substrates, as we have assessed for lysozyme. Actually, it was previously described that several alkylating agents are able to selectively modify the redox active Cys or Sec of TrxR, thus causing irreversible inhibition.^[43]^

## Conclusions

We have described a synthetic strategy to obtain a family of diiron carbonyl complexes containing a bridging aminocarbyne/iminium hybrid ligand from the readily available [Fe_2_Cp_2_(CO)_4_]. They have ideal properties for a potential anticancer drug, such as the presence of a nontoxic metal element, appreciable water solubility and amphiphilicity, and stability in aqueous media.^[44]^ Moreover, the synthesis works up to multigram scales and features a considerable structural variability. In general, the complexes showed moderate cytotoxic activity against human cancer cells and the ability to overcome resistance. Compound [**2D**]CF_3_SO_3_, with a 4‐methoxy phenyl substituent on the bridging hydrocarbyl ligand, was revealed to be the most promising and exhibited a noteworthy selectivity toward cancer cell lines compared to noncancerous cells. According to cellular‐uptake experiments, such selectivity is not correlated to a different ability to enter cancerous or noncancerous cells. Remarkably, the cytotoxicity profile of the complexes does not substantially decrease in 3D cell cultures, in contrast to what has often been observed in the literature upon comparison of 2D/3D studies on various transition metal compounds. Interestingly, the activity exhibited by [**2D**]CF_3_SO_3_ in the ovarian cancer cell 3D model was even superior to that of cisplatin. Experiments on selected complexes outlined their power to behave as carbon monoxide‐releasing molecules (CORMs) inside cells, to unbalance cellular redox homeostasis and to alkylate biological targets. The preferential CO release in cancer cells with respect to untransformed cells could, at least in part, be consistent with the ability to target TrxR. Actually, it is well known that the Trx system is overexpressed in cancer cells, and has been recognized as a tumour‐specific target for the development of selective anticancer agents. In summary, we suggest that a simple diiron platform might offer an arsenal of tools not available for widely investigated ferrocenes, paving the way for the development of iron‐based drugs with optimized features. Further biochemical and biophysical studies will be performed to clarify in more detail the mechanism of action of this new class of organometallic anticancer candidates.

## Experimental Section

**General experimental details**: [Fe_2_Cp_2_(CO)_4_] (99 %) was purchased from Strem Chemicals, other reactants and solvents were obtained from Alfa Aesar, Merck, Apollo Scientific or TCI Chemicals and were of the highest purity available. Isocyanides were stored at 4 °C or −20 °C and used as received; contaminated labware was treated with bleach. Methyl triflate was stored under N_2_ at 4 °C; allyl iodide and triethyloxonium tetrafluoroborate (1 M solution in CH_2_Cl_2_) were stored under N_2_ at −20 °C. Compounds [**1A**–**H**]^[19]^ and [**2D**–**F**]CF_3_SO_3_[^20^, ^21^] were prepared according to published procedures. Alkylation reactions were carried out under dry N_2_ using standard Schlenk techniques and solvents distilled over appropriate drying agents (MeCN from CaH_2_, CH_2_Cl_2_ from P_2_O_5_). The synthesis and chromatographic purification of **6** was carried out under N_2_ using deaerated solvents. Anion‐exchange reactions and all other operations were conducted under air with common laboratory glassware. Chromatography separations were carried out on alumina columns (Merck; neutral, 4 % w/w water content except where otherwise noted). Ion‐exchange chromatography was performed with an Amberlyst® 15 hydrogen form resin (Superlco/Merck), pre‐activated with a methanolic solution of NaOH. Once isolated, compounds [**2B**]X (X=CF_3_SO_3_, BF_4_), [**2C**]Cl, [**5H**]CF_3_SO_3_ (hygroscopic) and **6** were stored under N_2_; all other Fe compounds being air‐ and moisture‐stable in the solid state. NMR spectra were recorded at 25 °C on a Bruker Avance II DRX400 instrument equipped with a BBFO broadband probe. Chemical shifts (expressed in parts per million) are referenced to the residual solvent peaks^[45]^ (^1^H, ^13^C) or to external standards^46^ (^14^N to CH_3_NO_2_; ^19^F to CFCl_3_, ^35^Cl to 1 M NaCl in D_2_O, ^31^P to 85 % H_3_PO_4_). In [D_6_]DMSO/D_2_O mixtures, ^1^H chemical shifts are referenced to the [D_5_]DMSO signal as in pure [D_6_]DMSO (*δ*/ppm=2.50); in D_2_O/CD_3_OD mixtures, ^1^H chemical shifts are referenced to the CD_3_OD residual peak as CH_3_OH in pure D_2_O (*δ*/ppm=3.34). ^1^H and ^13^C spectra were assigned with the assistance of ^1^H{^31^P}, ^13^C DEPT 135, ^1^H,^1^H COSY, ^1^H‐^13^C *gs*‐HSQC and ^1^H‐^13^C *gs*‐HMBC experiments.^[47]^ CDCl_3_ stored in the dark over Na_2_CO_3_ was used for NMR analysis. IR spectra of solid samples (650–4000 cm^−1^) were recorded on a Perkin Elmer Spectrum One FTIR spectrometer, equipped with a UATR sampling accessory; IR spectra of solutions were recorded using a CaF_2_ liquid transmission cell (2300–1500 cm^−1^) on a Perkin Elmer Spectrum 100 FTIR spectrometer. UV/Vis spectra (250–800 nm) were recorded on a Ultraspec 2100 Pro spectrophotometer using PMMA cuvettes (1 cm path length). IR and UV/Vis spectra were processed with Spectragryph software.^[48]^ Carbon, hydrogen and nitrogen analyses were performed on a Vario MICRO cube instrument (Elementar). GC analysis was performed on a Clarus 500 instrument (PerkinElmer) equipped with a 5 Å MS packed column (Supelco) and a TCD detector. Samples were analysed by isothermal runs (110 °C, 4 min) using He as carrier gas. Raman analysis was conducted with a Renishaw Invia micro‐Raman instrument equipped with a Nd:YAG laser working at 532 nm and 0.1 mW, integration time 10 s. HPLC‐MS analyses were performed with a API3000 instrument (SCIEX) equipped with ESI(+) source and a quadrupole detector. HPLC separation was performed using an Agilent 1110 series instrument (Agilent Technologies Deutschland GmbH, Germany) equipped with a G1312A binary pump, a G1329A autosampler, a peltier column oven Series 200 (PerkinElmer) and a BioZen C4 intact column (3.6 μm, 150×2.1 mm, pore size 200 Å; Phenomenex, USA).

### Synthesis and characterization of compounds

**[Fe_2_Cp_2_(CO)_3_(CNR)], [1A**–**H]**.^[19]^ *General procedure*. In a 250 mL round‐bottom flask equipped with a reflux condenser under N_2_, the selected isocyanide, or its solution in MeCN (10 mL, for solid compounds), was added dropwise to a suspension of [Fe_2_Cp_2_(CO)_4_] (1.6 equiv) in anhydrous MeCN (80–100 mL). Alkyl isocyanides: the mixture was heated at reflux (T≥100 °C) for 8 h, then stirred at room temperature for additional 14 h. Aryl isocyanides: the mixture was stirred at room temperature for 14 h then heated at reflux (T≥100 °C) for 1–3 h. Next, conversion was checked by IR and the dark red‐brown suspension was dried under vacuum (40 °C). The resulting solid, consisting of a mixture of [Fe_2_Cp_2_(CO)_4_] and [Fe_2_Cp_2_(CO)_3_(CNR)], was directly used in the following alkylation procedure without any purification.

**[Fe_2_Cp_2_(CO)_2_(μ‐CO){μ‐CNMe(1H‐indol‐6‐yl)}]CF_3_SO_3_, [2A]CF_3_SO_3_**


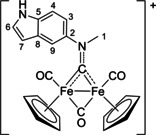


In a 150 mL Schlenk tube under N_2_, CF_3_SO_3_Me (0.11 mL, 1.0 mmol) was added dropwise to a dark red suspension of **1A** (0.975 mmol) in anhydrous CH_2_Cl_2_ (50 mL) under stirring. Therefore, the mixture was stirred at room temperature for 6 h and conversion was checked by IR (CH_2_Cl_2_). Next the suspension (red solution+solid) was moved on top of an alumina column (*h* 7, *d* 4.3 cm). Impurities were eluted with neat CH_2_Cl_2_ and THF, then a raspberry red band containing the title product was eluted with neat MeCN. Volatiles were removed under vacuum and residue was triturated in Et_2_O. The suspension was stirred at room temperature for a few hours and then filtered. The resulting red solid was washed with toluene, Et_2_O and dried under vacuum (40 °C). Yield: 358 mg, 71 %. Compound [**2A**]CF_3_SO_3_ is soluble in MeCN, THF, MeOH, CH_2_Cl_2_, less soluble in CH_2_Cl_2_, CHCl_3_, insoluble in Et_2_O, toluene and water. X‐ray quality crystals of [**2A**]CF_3_SO_3_ were obtained from an acetone solution layered with Et_2_O and settled aside at −20 °C. Anal. calcd. for C_25_H_21_F_3_Fe_2_N_2_O_6_S: C 46.47, H 3.28, N 4.34; found: C 46.09, H 3.28, N 4.34. IR (solid state): $\tilde{\nu }$ /cm^−1^=3330w‐br (*ν*_NH_), 3114w, 2004 s (*ν*_CO_), 1985 s (*ν*_CO_), 1833 s (*ν*_μ‐CO_), 1583w‐sh, 1555 m, 1540 m (*ν*_CN_), 1475w, 1458w, 1433w, 1420w, 1397 m, 1361w, 1345w, 1323w, 1290–1276 m, 1244 s (*ν*_SO3_), 1223 s (*ν*_SO3_), 1154 s (*ν*_SO3_), 1107 m‐sh, 1067w, 1028 s, 1016 s‐sh, 1002 m‐sh, 942w, 931w, 893w, 875w, 860 m, 845 m, 817w, 797 m, 772 s, 756w‐sh, 743 m‐sh, 728 m. IR (CH_2_Cl_2_): $\tilde{\nu }$ /cm^−1^=2022 s (*ν*_CO_), 1992 m‐sh (*ν*_CO_), 1836 s (*ν*_μ‐CO_), 1554 m, 1542 m (*ν*_CN_). IR (MeCN): $\tilde{\nu }$ /cm^−1^=2023 s (*ν*_CO_), 1991 m‐sh (*ν*_CO_), 1836 s (*ν*_μ‐CO_), 1554 m, 1542 m‐sh (*ν*_CN_). ^1^H NMR (CD_3_CN): *δ*/ppm=9.78 (s‐br, 1H, NH), 7.85 (s‐br, 1H, C9‐H), 7.70 (d, ^3^ *J*_HH_=8.6 Hz, 1H, C4‐H), 7.48 (t, ^3^ *J*_HH_=2.7 Hz, 1H, C6‐H), 7.37 (d‐br, ^3^ *J*_HH_=7.2 Hz, 1H, C3‐H), 6.67−6.62 (m, 1H, C7); 5.39, 4.65 (s, 10H, Cp), 4.53 (s, 3H, C1). ^13^C{^1^H} NMR (CD_3_CN): *δ*/ppm=324.9 (CN), 255.9 (μ‐CO); 209.8, 209.2 (CO); 144.6 (C2), 136.3 (C5), 129.1 (C8), 128.8 (C6), 118.8 (C3), 117.3 (C9), 113.8 (C4), 103.5 (C7); 91.2, 91.0 (Cp); 58.5 (C1). ^19^F{^1^H} NMR (CD_3_CN): *δ*/ppm=−79.3. ^1^H NMR (CDCl_3_): *δ*/ppm=9.57 (s‐br, 1H, NH), 8.0–7.0 (br, C3‐H+C9‐H), ( 7.65 (d, ^3^ *J*_HH_=8.4 Hz, 1H, C4‐H), 7.42–7.39 (m, 1H, C6‐H), 6.60 (s‐br, 1H, C7‐H); 5.41, 4.63 (s, 10H, Cp); 4.56 (s, 3H, C1‐H).

### [Fe_2_Cp_2_(CO)_2_(μ‐CO){μ‐CNMe(CH_2_PO_3_Et_2_)}]X, [2B]X (X=BF_4_, CF_3_SO_3_)


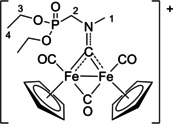


**[2B]CF_3_SO_3_**. In a 150 mL Schlenk tube under N_2_, CF_3_SO_3_Me (0.20 mL, 1.8 mmol) was added dropwise to a dark red solution of **1B** (1.38 mmol) in anhydrous CH_2_Cl_2_ (40 mL) under stirring. Therefore, the mixture was stirred at room temperature for 7 h and conversion was checked by IR (CH_2_Cl_2_). Next, the solution was moved on top of an alumina column (*h* 8, *d* 5.5 cm). Impurities were eluted with neat CH_2_Cl_2_, THF and MeCN, then a red band containing the title product was eluted with MeCN/MeOH (10 : 1, *v/v*). Volatiles were removed under vacuum (40 °C), affording a red, highly hygroscopic solid. Yield: quantitative. IR (CH_2_Cl_2_): $\tilde{\nu }$ /cm^−1^=2028 s (*ν*_CO_), 1997w‐sh (*ν*_CO_), 1836 m (*ν*_μ‐CO_). ^1^H NMR (CDCl_3_): *δ*/ppm=5.32 (s, 10H, Cp), 5.23 (dd, ^2^ *J*_HH_=14.9 Hz, ^2^ *J*_HP_=5.3 Hz, 1H, C2‐H), 4.93 (t, ^2^ *J*_HH_=^2^ *J*_HP_=15.5 Hz, 1H, C2‐H’), 4.38 (s, 3H, C1‐H), 4.23–4.10 (m, 4H, C3‐H), 1.30 (q, ^4^ *J*_HP_=^3^ *J*_HH_=7.1 Hz, 6H, C4‐H). ^19^F{^1^H} NMR (CDCl_3_): *δ*/ppm=−78.2. ^31^P{^1^H} NMR (CDCl_3_): *δ*/ppm=17.3.

**[2B]BF_4_**. In a 500 mL round bottom flask, [**2B**]CF_3_SO_3_ was suspended in water (100 mL) with vigorous stirring until completely dissolved then treated with NaBF_4_ (500 mg). The red solution was extracted with CH_2_Cl_2_ (3×20 mL) and the combined organic fractions were dried under vacuum. The residue was suspended in water and the ion exchange procedure with NaBF_4_ was repeated (×3); ^19^F{^1^H} NMR analysis of the final CH_2_Cl_2_ solution indicated the complete removal of CF_3_SO_3_^−^ anions. Therefore, volatiles were removed under vacuum; the residue was dissolved in CH_2_Cl_2_ and filtered over celite. A red foamy hygroscopic solid, obtained upon volatiles removal without heating, was dried under vacuum and stored under N_2_. Yield: 751 mg, 90 % (with respect to **1B**). Compound [**2B**]BF_4_ is soluble in MeOH, MeCN, CH_2_Cl_2_, CHCl_3_, water, insoluble in Et_2_O and hexane. Anal. calcd. for C_20_H_25_BF_4_Fe_2_NO_6_P: C 39.71, H 4.17, N 2.31; found: C 39.20, H 4.25, N 2.26. IR (solid state): $\tilde{\nu }$ /cm^−1^=3117w, 2987w, 2938w, 2913w, 2875w, 2012 s (*ν*_CO_), 1986 s‐sh (*ν*_CO_), 1823 s (*ν*_μ‐CO_), 1568 m (*ν*_CN_), 1479w, 1446w, 1435w, 1421w, 1399 m, 1370w‐sh, 1297–1286w, 1254 m, 1225 m‐sh, 1180w, 1163w, 1075 m‐sh, 1033 s‐sh (*ν*_BF4_), 1008 s, 973 s, 951 s‐sh, 904 m, 864 m‐sh, 851 m, 837 m‐sh, 780s, 745 s, 710 m‐sh. IR (CH_2_Cl_2_): $\tilde{\nu }$ /cm^−1^=2028 s (*ν*_CO_), 1996w (*ν*_CO_), 1836 m (*ν*_μ‐CO_), 1574w (*ν*_CN_). IR (MeCN): $\tilde{\nu }$ /cm^−1^=2026 s (*ν*_CO_), 1994w (*ν*_CO_), 1838 m (*ν*_μ‐CO_), 1569w (*ν*_CN_). ^1^H NMR (CDCl_3_): *δ*/ppm=5.29 (s, 10H, Cp), 5.15 (dd, ^2^ *J*_HH_=16.4 Hz, ^2^ *J*_HP_=4.0 Hz, 1H, C2‐H), 4.93 (t, ^2^ *J*_HH_=^2^ *J*_HP_=15.6 Hz, 1H, C2‐H’), 4.35 (s, 3H, C1‐H), 4.24–4.10 (m, 4H, C3‐H), 1.30 (dt, ^4^ *J*_HP_=9.8 Hz, ^3^ *J*_HH_=7.1 Hz, 6H, C4‐H). No changes were observed in the ^1^H spectrum after 14 h at room temperature. ^13^C{^1^H} NMR (CDCl_3_): *δ*/ppm=323.7 (d, ^3^ *J*_CP_=6.4 Hz, CN), 254.7 (μ‐CO); 207.4, 207.1 (CO); 90.3, 90.1 (Cp); 63.3 (C3), 62.5 (d, ^1^ *J*_CP_=146 Hz, C2), 53.2 (C1), 16.4 (t, ^3^ *J*_CP_=5.6 Hz, C4). ^19^F{^1^H} NMR (CDCl_3_): *δ*/ppm=‐151.9 (^10^BF_4_), −152.0 (^11^BF_4_). ^31^P{^1^H} NMR (CDCl_3_): *δ*/ppm=17.3.

### [Fe_2_Cp_2_(CO)_2_(μ‐CO){μ‐CNMe(C_6_H_11_)}]X, [2C]X (X=CF_3_SO_3_, Cl)


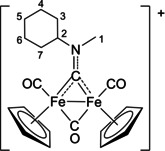


**[2C]CF_3_SO_3_**. In a 500 mL round bottom Schlenk flask under N_2_, CF_3_SO_3_Me (2.8 mL, 26 mmol) was added dropwise to a dark red suspension of **1C** (22.5 mmol) in anhydrous CH_2_Cl_2_ (100 mL) under stirring. Therefore, the mixture was stirred at room temperature for 4 h and the conversion was checked by IR (CH_2_Cl_2_). Next, the dark red solution was moved on top of an alumina column (*h* 13, *d* 6 cm). Impurities were eluted with neat CH_2_Cl_2_ and THF, then a red band containing [**2C**]CF_3_SO_3_ was eluted with MeCN/MeOH (20 : 1, *v/v*). A red band, containing a minor fraction of [**2C**]Cl, was collected using MeOH as eluent. Volatiles were removed under vacuum from the MeCN/MeOH solution and residue was triturated in Et_2_O. The suspension was stirred at room temperature for a few h then filtered. The resulting red solid was washed with toluene, Et_2_O and dried under vacuum (40 °C). Yield: 11.44 g, 85 %. Compound [**2C**]CF_3_SO_3_ is soluble in MeCN, MeOH, CH_2_Cl_2_, CHCl_3_, less soluble in water, poorly soluble in toluene, insoluble in Et_2_O, hexane. X‐ray‐quality crystals of [**2C**]CF_3_SO_3_ were obtained from a CH_2_Cl_2_ solution layered with heptane and settled aside at −20 °C. Anal. calcd. for C_22_H_24_F_3_Fe_2_NO_6_S: C 44.10, H 4.04, N, 2.34; found: C 44.47, H 4.04, N, 2.34. IR (solid state): $\tilde{\nu }$ /cm^−1^=3102w, 2937w, 2862w, 2185w, 2146w, 2006 s (*ν*_CO_), 1982 s‐sh (*ν*_CO_), 1822 s (*ν*_μ‐CO_), 1565 m (*ν*_CN_), 1544 m‐sh, 1452w, 1435w, 1421w, 1402w, 1366w, 1352w, 1321w, 1272 s‐sh, 1257 s (*ν*_SO3_), 1223 s‐sh (*ν*_SO3_), 1150s (*ν*_SO3_), 1056 m, 1029 s, 990 m, 864 m, 854 m, 796 s, 746 s. IR (CH_2_Cl_2_): $\tilde{\nu }$ /cm^−1^=2020s (*ν*_CO_), 1988w‐sh (*ν*_CO_), 1835 m (*ν*_μ‐CO_), 1567w (*ν*_CN_). IR (MeCN): $\tilde{\nu }$ /cm^−1^=2021 s (*ν*_CO_), 1988w‐sh (*ν*_CO_), 1835 m (*ν*_μ‐CO_), 1568w (*ν*_CN_). ^1^H NMR (CDCl_3_): *δ*/ppm=5.36, 5.27 (s, 10H, Cp); 4.68 (t, ^3^ *J*_HH_=11.7 Hz, 1H, C2‐H), 4.06 (s, 3H, C1‐H), 2.81 (d, *J*=11.8 Hz, 1H, C4‐H), 2.15 (d, *J*=14.6 Hz, 1H, C6‐H), 2.03–1.94 (m, 2H, C3‐H+C4‐H’), 1.86–1.72 (m, 3H, C7‐H+C5‐H), 1.53–1.35 (m, 3H, C3‐H’+C6‐H’), 1.34–1.20 (m, 1H, C7‐H’). No changes were observed in the ^1^H spectrum after 14 h at room temperature. ^13^C{^1^H} NMR (CDCl_3_): *δ*/ppm=316.4 (CN), 255.3 (μ‐CO); 208.5, 207.6 (CO); 90.3, 90.1 (Cp); 79.8 (C2), 46.8 (C1), 31.2 (C5), 30.7 (C4), 26.1 (C3), 26.0 (C6), 25.1 (C7). ^19^F{^1^H} NMR (CDCl_3_): *δ*/ppm=−78.2. ^1^H NMR (CD_3_OD): *δ*/ppm=5.40, 5.36 (s, 10H, Cp), 4.07 (s, 3H, C1‐H), 2.41 (d, *J*=11.1 Hz, 1H, C4‐H), 2.17–2.07, 2.03–1.91, 1.85–1.50, 1.42–1.32 (m, C_6_H_11_).

**[2C]Cl**. Compound [**2C**]CF_3_SO_3_ (110 mg, 0.184 mmol) was dissolved in MeOH (2 mL) then moved on top of an Amberlyst 15 column (Na^+^ form; *h* 7 cm, *d* 2.3 cm). NaCF_3_SO_3_ was eluted with neat MeOH then a red band containing [**2C**]^+^ was eluted with NaCl‐saturated MeOH. Volatiles were removed under vacuum and the residue was suspended in MeCN. The suspension was filtered over celite; the filtrate was checked for absence of CF_3_SO_3_^−^ by ^19^F NMR then dried under vacuum (40 °C). The resulting red, hygroscopic solid was stored under N_2_. Yield: 83 mg, 93 %. Compound [**2C**]Cl shows the same solubility pattern as the triflate salt. X‐ray quality crystals of [**2C**]Cl ⋅ 2.34H_2_O were obtained from a CH_2_Cl_2_ solution layered with hexane and settled aside at −20 °C. Anal. calcd. for C_21_H_24_ClFe_2_NO_3_: C 51.94, H 4.98, N 2.88; found: C 51.99, H 5.02, N 2.83. IR (solid state): $\tilde{\nu }$ /cm^−1^=3376 m‐br* (*ν*_OH_), 3098–3059w, 2933 m, 2858w, 2180–2160w, 1997 s (*ν*_CO_), 1969 s‐sh (*ν*_CO_), 1811 s (*ν*_μ‐CO_), 1662w, 1631w, 1565 s (*ν*_CN_), 1543 m, 1450 m, 1434w, 1419 m, 1400 m, 1350w, 1319w, 1263w‐sh, 1249w, 1173w, 1154w, 1054 m, 1026w, 1015w, 990 m, 924w‐sh, 893w‐sh, 852 m, 795 s, 746 s, 729 s‐sh. *Due to moisture. IR (CH_2_Cl_2_): $\tilde{\nu }$ /cm^−1^=2018 s (*ν*_CO_), 1985w‐sh (*ν*_CO_), 1833 m (*ν*_μ‐CO_), 1569w (*ν*_CN_). IR (MeCN): $\tilde{\nu }$ /cm^−1^=2020s (*ν*_CO_), 1987w‐sh (*ν*_CO_), 1834 m (*ν*_μ‐CO_), 1568w (*ν*_CN_). ^1^H NMR (CDCl_3_): *δ*/ppm=5.51, 5.39 (s, 10H, Cp); 4.75–4.66 (m, 1H, C2‐H), 4.14 (s, 3H, C1‐H), 3.16 (d, ^2^ *J*_HH_=13.4 Hz, 1H, C4‐H), 2.15 (d, ^2^ *J*_HH_=14.2 Hz, 1H, C6‐H), 2.03–1.93 (m, 2H, C3‐H+C4‐H’), 1.84–1.72 (m, 3H, C7‐H+C5‐H), 1.51–1.35 (m, 2H, C3‐H’+C6‐H’), 1.33–1.23 (m, 1H, C7‐H’). No changes were observed in the ^1^H spectrum after 24 h at room temperature. ^13^C{^1^H} NMR (CDCl_3_): *δ*/ppm=316.2 (CN), 255.8 (μ‐CO); 208.7, 207.8 (CO); 90.7, 90.3 (Cp); 79.7 (C2), 47.1 (C1), 31.3 (C5), 31.0 (C4), 26.1 (C3), 26.0 (C6), 25.2 (C7). ^35^Cl NMR (CDCl_3_): *δ*/ppm=7.61 (Δ*ν*_1/2_=266 Hz).

### [Fe_2_Cp_2_(CO)_2_(μ‐CO){μ‐CNMe(4‐C_6_H_4_OMe)}]CF_3_SO_3_, [2D]CF_3_SO_3_


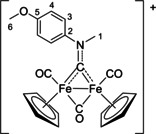


The title compound was prepared with minor modifications of the literature procedure.^[21]^ In a 250 mL Schlenk tube under N_2_, CF_3_SO_3_Me (1.0 mL, 5.5 mmol) was added dropwise to a dark red solution of **1B** (4.63 mmol) in anhydrous CH_2_Cl_2_ (40 mL) under stirring. The mixture was stirred at room temperature for 5.5 h then moved on top of an alumina column (Brockmann activity I; *h* 6.0, *d* 4.3 cm). Impurities were eluted with CH_2_Cl_2_ and CH_2_Cl_2_/THF (1 : 1, *v*/*v*), then a red band containing [**2D**]CF_3_SO_3_ was eluted with neat MeCN. Volatiles were removed under vacuum (40 °C) to afford a red foamy solid. Yield: 2.446 g, 85 %. Compound [**2D**]CF_3_SO_3_ is soluble in MeCN, MeOH, CH_2_Cl_2_, CHCl_3_, water, insoluble in Et_2_O and hexane. X‐ray quality crystals of [**2D**]CF_3_SO_3_ were obtained from a CHCl_3_ solution layered with hexane and settled aside at −20 °C. Anal. calcd. for C_23_H_20_F_3_Fe_2_NO_7_S: C 44.33, H 3.24, N 2.25; found: C 43.92, H 3.31, N 2.17. IR (solid state): $\tilde{\nu }$ /cm^−1^=3113w, 3091w, 2946w, 2849w, 2011 s (*ν*_CO_), 1978 s (*ν*_CO_), 1821 s (*ν*_μ‐CO_), 1608w, 1563 m, 1539 m (*ν*_CN_), 1506 s, 1468w, 1449w, 1433w, 1420w, 1395 m, 1304w‐sh, 1278 s, 1251 s (*ν*_SO3_), 1224 s‐sh (*ν*_SO3_), 1176 m‐sh, 1146 s (*ν*_SO3_), 1117 m‐sh, 1108 m, 1067w, 1028 s, 890w, 855 s, 825w‐sh, 773 s, 756 m‐sh, 736w, 702 s. IR (CH_2_Cl_2_): $\tilde{\nu }$ /cm^−1^=2021 s (*ν*_CO_), 1989w (*ν*_CO_), 1836 m (*ν*_μ‐CO_), 1607w, 1562w, 1540w (*ν*_CN_), 1507 m. IR (MeCN): $\tilde{\nu }$ /cm^−1^=2024 s (*ν*_CO_), 1991w (*ν*_CO_), 1837 m (*ν*_μ‐CO_), 1610w, 1563w, 1539w (*ν*_CN_), 1508w. ^1^H NMR (CDCl_3_): *δ*/ppm=8.3–7.6 (br, 2H, C3‐H), 7.10 (d, ^3^ *J*_HH_=7.4 Hz, 2H, C4‐H); 5.43, 4.78 (s, 10H, Cp); 4.54 (s, 3H, C1‐H), 3.90 (s, 3H, C6‐H). ^19^F{^1^H} NMR (CDCl_3_): *δ*/ppm=−78.1.

### [Fe_2_Cp_2_(CO)_2_(μ‐CO){μ‐CNMe(CH_2_CHCH_2_)}]X, [3F]X (X=I, CF_3_SO_3_)


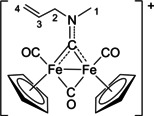


**[3F]I**. The preparation of the title compound was optimized with respect to the literature (69 % yield with 10–30 equiv. of allyl iodide).^[22]^ In a 150 mL Schlenk tube under N_2_, allyl iodide (0.39 mL, 4.3 mmol) was added dropwise to a dark red solution of **1F** (1.54 mmol) in anhydrous MeCN (30 mL) under vigorous stirring. The mixture was stirred for 4.5 h under protection from the light and conversion was checked by IR (MeCN). Next, the dark red solution was moved on top of an alumina column (Brockmann activity I; *h* 6.5, *d* 4.3 cm). Impurities were eluted with neat Et_2_O, THF and MeCN, then a red band containing the title product was eluted with MeCN/MeOH (10 : 1, *v/v*). Volatiles were removed under vacuum (40 °C) and the residue was triturated in Et_2_O. The suspension was stirred at room temperature then filtered. The resulting red solid was washed with Et_2_O and dried under vacuum (40 °C). Yield: 646 mg, 78 %. Compound [**3F**]I is soluble in MeOH, CH_2_Cl_2_, CHCl_3_, poorly soluble in water, insoluble in Et_2_O, hexane. IR (CH_2_Cl_2_): $\tilde{\nu }$ /cm^−1^=2021 s (*ν*_CO_), 1989w (*ν*_CO_), 1835 m (*ν*_μ‐CO_), 1585w (*ν*_CN_). ^1^H NMR (CDCl_3_): *δ*/ppm=6.09–5.97 (m, 1H, CH=C, C3‐H), 5.53–5.46 (m, 3H, C2‐H+C4‐H); 5.45, 5.53 (s, 10H, Cp); 5.17–5.11 (m, 1H, C2‐H’), 4.25 (s, 3H, C1‐H).

**[3F]CF_3_SO_3_**. In a 50 mL round bottom flask, a solution of Ag(CF_3_SO_3_) (468 mg, 1.79 mmol) in MeCN (5 mL) was added to a dark red suspension of [**3F**]I (950 mg, 1.78 mmol) in MeCN (6 mL) at 0 °C. The mixture stirred at 0 °C for 2.5 h while maintaining the system under protection from the light. The resulting suspension (dark red solution+yellow AgI precipitate) was filtered over celite. Volatiles were removed under vacuum from the filtrate solution and the residue was triturated in a Et_2_O/toluene mixture. The suspension was stirred at room temperature for a few h, then filtered. The resulting red solid was washed with toluene, Et_2_O and dried under vacuum (40 °C). Yield: 928 mg, 94 %. Compound [**3F**]CF_3_SO_3_ is soluble in MeCN, acetone, MeOH, water, less soluble in CH_2_Cl_2_ > CHCl_3_, poorly soluble in toluene, insoluble in Et_2_O. Anal. calcd. for C_19_H_18_F_3_Fe_2_NO_6_S: C 40.96, H, 3.26, N, 2.51; found: C 41.20, H, 3.20, N, 2.72. IR (solid state): $\tilde{\nu }$ /cm^−1^=3106w, 2213w, 2178w, 2012 s (*ν*_CO_), 1988 s (*ν*_CO_), 1813 s (*ν*_μ‐CO_), 1644w, 1581 m (*ν*_CN_), 1435w, 1416w, 1395w, 1261 s (*ν*_SO3_), 1243 s‐sh, 1222 m‐sh (*ν*_SO3_), 1169 s (*ν*_SO3_), 1050 m, 1028 s, 989w‐sh, 931 m, 888 m, 854 m, 758 s, 666 m. IR (CH_2_Cl_2_): $\tilde{\nu }$ /cm^−1^=2022 s (*ν*_CO_), 1990w (*ν*_CO_), 1836 m (*ν*_μ‐CO_), 1584 m (*ν*_CN_). IR (MeCN): $\tilde{\nu }$ /cm^−1^=2023 s (*ν*_CO_), 1990w (*ν*_CO_), 1836 m (*ν*_μ‐CO_), 1582 m (*ν*_CN_). ^1^H NMR (CDCl_3_): *δ*/ppm=5.98 (dddd, ^3^ *J*_HH_=17.4, 10.0, 7.5, 5.0 Hz, 1H, C3‐H), 5.50–5.45 (m, 2H, C4‐H+C4‐H’); 5.34, 5.32 (s, 10H, Cp); 5.30–5.23 (m, 1H, C2‐H), 5.15–5.08 (m, 1H, C2‐H’), 4.17 (s, 3H, C1‐H). No changes were observed in the ^1^H spectrum after 14 h at room temperature. ^13^C{^1^H} NMR (CDCl_3_): *δ*/ppm=318.1 (CN), 255.3 (μ‐CO); 208.1, 207.8 (CO), 130.2 (C3), 122.2 (C4), 121.0 (^1^ *J*_CF_=319 Hz, CF_3_); 90.20, 90.16 (Cp); 70.6 (C2), 51.3 (C1). ^19^F{^1^H} NMR (CDCl_3_): *δ*/ppm=−78.2. ^1^H NMR ([D_6_]acetone): *δ*/ppm=6.33–6.19 (m, 1H, C3‐H), 5.62 (d, ^3^ *J*_HH_=17.2 Hz, 1H, C4‐H); 5.58, 5.54 (s, 10H, Cp); 5.41–5.16 (m, 3H, C2‐H+C2‐H’+C4‐H’), 4.28 (s, 3H, C1‐H).

### [Fe_2_Cp_2_(CO)_2_(μ‐CO){μ‐CNMe(2‐naphthyl)}]I, [3G]I


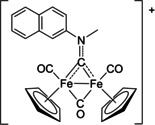


In a 25 mL Schlenk tube under N_2_, methyl iodide (110 mL, 1.8 mmol) was added to a dark red solution of **1G** (0.48 mmol) in anhydrous MeCN (8 mL). The mixture was stirred for 2.5 h and conversion was checked by IR (MeCN). Next, the dark red solution was moved on top of an alumina column (*h* 10, *d* 2 cm). Impurities were eluted with neat CH_2_Cl_2_ and THF, then a red band containing the title product was eluted with THF/MeOH (4 : 1, *v/v*). Next, volatiles were removed under vacuum and the residue was dissolved in CH_2_Cl_2_. The suspension was filtered over celite and the filtrate was taken to dryness under vacuum (40 °C), affording a dark red solid. Yield: 208 mg, 69 %. A considerable decrease in yield and purity was observed for reactions carried out in refluxing CHCl_3_ or in MeCN at lower temperatures. Anal. calcd. for C_25_H_20_Fe_2_INO_3_: C 48.35, H 3.25, N 2.56; found: C 48.20, H 3.15, N 2.49. Compound [**3G**]I is soluble in MeOH, CH_2_Cl_2_, CHCl_3_, sparingly soluble in water, insoluble in hexane. IR (CH_2_Cl_2_): $\tilde{\nu }$ /cm^−1^=2021 s (*ν*_CO_), 1989 m‐sh (*ν*_CO_), 1836 m (*ν*_μ‐CO_), 1564w, 1545w, 1538w (*ν*_CN_). ^1^H NMR (CDCl_3_): *δ*/ppm=8.10 (d+m, 2H), 7.98−7.95 (m, 1H), 7.94−7.77 (m, 1H), 7.66−7.52 (m, 2H), 7.55−7.47 (m, 1H) (C_10_H_7_); 5.62, 4.82 (s, 10H, Cp); 4.75 (s, 3H, NCMe).

### [Fe_2_Cp_2_(CO)_2_(μ‐CO){μ‐CNEt(2‐naphthyl)}]BF_4_, [4G]BF_4_


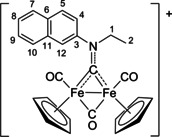


In a 150 mL Schlenk tube under N_2_, [Et_3_O]BF_4_ (1 M solution in CH_2_Cl_2_; 0.71 mL, 0.71 mmol), was added dropwise to a dark red solution of **1G** (0.724 mmol) in anhydrous MeCN (30 mL) at 0 °C under vigorous stirring. Therefore, the mixture was stirred for 3 h, while allowing to reach room temperature. Next, conversion was checked by IR (MeCN) then volatiles were removed under vacuum. The residue was dissolved in a small volume of THF then moved on top of an alumina column (*h* 6.5, *d* 4.3 cm). Impurities were eluted with neat Et_2_O and THF, then a red band containing the title product was eluted with MeCN/MeOH (10 : 1, *v/v*). Volatiles were removed under vacuum (40 °C) and the residue was triturated in Et_2_O. The suspension was stirred at room temperature for a few h then filtered. The resulting dark red solid was washed with Et_2_O and dried under vacuum (40 °C). Yield: 284 mg, 66 %. A considerable decrease in yield and purity was observed for reactions carried out in CH_2_Cl_2_ or in MeCN at room temperature. Compound [**4G**]BF_4_ is soluble in MeOH, MeCN, CH_2_Cl_2_, CHCl_3_, poorly soluble in Et_2_O, insoluble in water. Anal. calcd. for C_26_H_22_BF_4_Fe_2_NO_3_: C 52.49, H 3.72, N 2.35; found: C 52.11, H 3.67, N 2.30. IR (solid state): $\tilde{\nu }$ /cm^−1^=3116w, 3060w, 2979w, 2937w, 2160w‐br, 2007 s (*ν*_CO_), 1937 s‐sh (*ν*_CO_), 1836 s (*ν*_μ‐CO_), 1596w, 1532 s (*ν*_CN_), 1506 m‐sh, 1458w, 1434w, 1421w, 1381w, 1360w, 1341w, 1275w, 1247w, 1211w, 1179w, 1134w, 1075 s‐sh, 1049 s‐br, 1033 s (*ν*_BF4_), 1014 s‐sh, 963w, 935w, 865w, 850w, 825w, 808 m, 745 s, 707 m, 657 m. IR (CH_2_Cl_2_): $\tilde{\nu }$ /cm^−1^=2021 s (*ν*_CO_), 1989w (*ν*_CO_), 1836 m (*ν*_μ‐CO_), 1538w (*ν*_CN_). IR (MeCN): $\tilde{\nu }$ /cm^−1^=2023 s (*ν*_CO_), 1990w (*ν*_CO_), 1837 m (*ν*_μ‐CO_), 1537w (*ν*_CN_). ^1^H NMR ([D_6_]acetone): *δ*/ppm=8.7–8.3 (br, 1H, C4‐H/C12‐H), 8.30 (d, ^3^ *J*_HH_=8.6 Hz, 1H, C5‐H), 8.16–8.09 (m, 2H, C7‐H+C10‐H), 8.1–7.7 (br, 1H, C4‐H/C12‐H), 7.75–7.70 (m, 2H, C8‐H+C9‐H), 5.68 (s, 5H, Cp), 5.19 (q, ^3^ *J*_HH_=7.0 Hz, 2H, C1‐H), 4.87 (s, 5H, Cp), 1.51 (t, ^3^ *J*_HH_=7.2 Hz, 3H, C2‐H). No changes were observed in the ^1^H spectrum after 14 h at room temperature. ^13^C{^1^H} ([D_6_]acetone): *δ*/ppm=325.2 (CN), 255.2 (μ‐CO); 210.1, 209.8 (CO); 146.1 (C3); 134.3, 133.9 (C6+C11); 131.4 (C5); 129.5, 129.0 (C7+C10); 128.8, 128.6 (C8+C9); 126.3, 125.1 (C4+C12); 91.7, 91.2 (Cp); 66.8 (C1), 14.4 (C2). ^19^F{^1^H} NMR ([D_6_]acetone): *δ*/ppm=−151.0 (^10^BF_4_), −151.1 (^11^BF_4_). ^1^H NMR (CDCl_3_): *δ*/ppm=8.5–7.8 (br, 4H), 7.70–7.60 (m, 2H), 7.60–7.48 (m‐br, 1H), 5.44 (s, 5H), 5.20–5.05 (s‐br, 1H), 4.95 (td, *J*=14.5, 7.3 Hz, 1H), 4.65 (s, 5H), 1.44 (t, *J*=7.3 Hz, 3H). ^13^C{^1^H} NMR (CDCl_3_): *δ*/ppm=323.2 (br), 254.9, 209.2 (br), 208.4, ca. 145 (br); 133 (br), 132.9 (br); 130.1 (m), 128.1, 127.9, 126–125 (m‐br), 124.0, 120.5, 117.1, 90.6, 90.3, 66 (br), 14.0. ^19^F{^1^H} NMR (CDCl_3_): *δ*/ppm=−150.9, −151.0. The *cis* orientation of the Cp ligands in [**4G**]BF_4_ was ascertained by ^1^H NOE experiment in CDCl_3_. Thus, irradiation of one Cp resonance (*δ*_H_ 5.44 or 4.65 ppm) evidenced a NOE interaction with the other.

### [Fe_2_Cp_2_(CO)_2_(μ‐CO){μ‐CNBn_2_}]X, [5H]X (X=Br, CF_3_SO_3_)


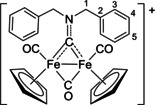


**[5H]Br**. In a 100 mL Schlenk tube under N_2_, benzyl bromide (1.0 mL, 8.4 mmol) was added dropwise to a dark red solution of **1H** (1.58 mmol) in anhydrous MeCN (50 mL) under vigorous stirring at 60 °C. The mixture was stirred at reflux for 1.5 h and conversion was checked by IR (MeCN). Next, the dark red solution was moved on top of an alumina column (*h* 6.5, *d* 5.5 cm). Impurities were eluted with neat CH_2_Cl_2_, THF and MeCN, then a red band containing the title product was eluted with MeCN/MeOH (10 : 1, *v/v*). Volatiles were removed under vacuum (40 °C), affording a red solid. Yield: 892 mg, 92 %. Compound [**5H**]Br is soluble in CH_2_Cl_2_, CHCl_3_, THF, *i*PrOH, poorly soluble in toluene, EtOAc, insoluble in Et_2_O. IR (CH_2_Cl_2_): $\tilde{\nu }$ /cm^−1^=2020s (*ν*_CO_), 1988w (*ν*_CO_), 1837 m (*ν*_μ‐CO_), 1550w (*ν*_CN_), 1534w. ^1^H NMR (CDCl_3_): *δ*/ppm=7.48 (t, ^3^ *J*_HH_=7.4 Hz, 4H, C4‐H), 7.40 (t, ^3^ *J*_HH_=7.3 Hz, 2H, C5‐H), 7.30–7.26 (m, 4H, C3‐H), 5.69 (d, ^2^ *J*_HH_=14.7 Hz, 2H, C1‐H), 5.56 (s, 10H, Cp), 5.52 (d, ^2^ *J*_HH_=14.7 Hz, 2H, C1‐H’).

**[5H]CF_3_SO_3_**. In a 50 mL round bottom flask, a solution of Ag(CF_3_SO_3_) (440 mg, 1.71 mmol) in MeCN (5 mL) was added to a dark red solution of [**5H**]I (1045 mg, 1.70 mmol) in MeCN (40 mL) at 0 °C. The mixture was stirred at 0 °C for 1.5 h while maintaining the system under protection from the light. The resulting suspension (dark red solution+pale yellow AgBr precipitate) was filtered over celite and the filtrate solution was dried under vacuum. The residue was treated with hexane (10 mL) then Et_2_O (50 mL) and settled aside for 30 min. Therefore, the solution was discarded and the washing procedure was repeated (x 2). The resulting red solid was dried under vacuum (40 °C) and stored under N_2_ (slightly hygroscopic). Yield: 1076 mg, 93 %. Compound [**5H**]CF_3_SO_3_ is soluble in MeCN, acetone, MeOH, CH_2_Cl_2_, poorly soluble in Et_2_O, toluene, water and insoluble in hexane. Anal. calcd. for C_29_H_24_F_3_Fe_2_NO_6_S: C 50.98, H, 3.54, N, 2.05; found: C 50.59, H, 3.58, N, 2.26. IR (solid state): $\tilde{\nu }$ /cm^−1^=3106w, 3070–3230w, 2166w, 2013 s (*ν*_CO_), 1985 s‐sh (*ν*_CO_), 1827 s (*ν*_μ‐CO_), 1588w, 1549 m (*ν*_CN_), 1533 m, 1497w, 1454w, 1433w, 1420w, 1352w, 1260s (*ν*_SO3_), 1223 s‐sh (*ν*_SO3_), 1153 s (*ν*_SO3_), 1096w, 1079w, 1029 s, 858 m, 767 s, 737 m‐sh, 698 s. IR (CH_2_Cl_2_): $\tilde{\nu }$ /cm^−1^=2023 s (*ν*_CO_), 1991 m (*ν*_CO_), 1840 m (*ν*_μ‐CO_), 1550w (*ν*_CN_), 1534w. IR (MeCN): $\tilde{\nu }$ /cm^−1^=2023 s (*ν*_CO_), 1990w (*ν*_CO_), 1839 m (*ν*_μ‐CO_), 1552w (*ν*_CN_), 1538w. ^1^H NMR (CDCl_3_): *δ*/ppm=7.50–7.44 (m, 4H, C4‐H), 7.44–7.39 (m, 2H, C5‐H), 7.20 (d, ^3^ *J*_HH_=7.3 Hz, 4H, C3‐H), 5.66 (d, ^2^ *J*_HH_=14.6 Hz, 2H, C1‐H), 5.50 (d, ^2^ *J*_HH_=14.7 Hz, 2H, C1‐H’), 5.40 (s, 10H, Cp). No changes were observed in the ^1^H spectrum after 2 days at room temperature. ^13^C{^1^H} NMR (CDCl_3_): *δ*/ppm=324.4 (CN), 253.7 (μ‐CO), 208.2 (CO), 132.1 (C2), 129.9 (C4), 129.4 (C5), 127.8 (C3), 121.1 (^1^ *J*_CF_=313 Hz, CF_3_), 90.6 (Cp), 68.9 (C1). ^19^F{^1^H} NMR (CDCl_3_): *δ*/ppm=−78.1 ppm.

### [Fe_2_Cp_2_Cl(CO)(μ‐CO){μ‐CNMe(C_6_H_11_)}], 6


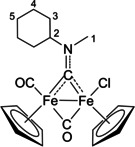


In a 25 mL Schlenk tube under N_2_, a red suspension of [**2C**]CF_3_SO_3_ (103 mg, 0.172), Me_3_NO ⋅ 2H_2_O (35 mg, 0.32 mmol) and LiCl (23 mg, 0.54 mmol) in acetone (8 mL) was stirred at reflux for 5 h. Conversion was checked by IR (acetone) then volatiles were removed under vacuum. The brown residue was suspended in CH_2_Cl_2_ and moved on top of an alumina column (*h* 2.5, *d* 4.3 cm), under N_2_. Impurities were eluted with CH_2_Cl_2_ then a brown band containing the title product was eluted with THF. The eluate was dried under vacuum and the residue was suspended hexane. The suspension was filtered; the resulting brown solid was washed with hexane and dried under vacuum (40 °C). Yield: 55 mg, 70 %. A related one‐pot reaction with [**2C**]CF_3_SO_3_/Me_3_NO ⋅ 2H_2_O (1.6 equiv)/NaCl (3.6 equiv) in deaerated MeOH (8 mL) at 40 °C overnight gave **6** in 43 % yield. A two‐step procedure comprising the [**2C**]CF_3_SO_3_/Me_3_NO ⋅ 2H_2_O (1 equiv) reaction in MeCN at room temperature followed by LiCl (3.0 equiv) in refluxing acetone for 3 h gave **6** in 54 % yield. Compound **6** is soluble in CH_2_Cl_2_, CHCl_3_, scarcely soluble in MeOH, insoluble in Et_2_O and hydrocarbons. Crystals of **6** for X‐ray analysis were obtained from a CHCl_3_ solution layered with pentane and settled aside at −20 °C. IR (CH_2_Cl_2_): $\tilde{\nu }$ /cm^−1^=1977 s (*ν*_CO_), 1798 s (*ν*_μ‐CO_), 1537 m (*ν*_CN_). IR (MeCN): $\tilde{\nu }$ /cm^−1^=1971 s (*ν*_CO_), 1795 m (*ν*_μ‐CO_), 1539w (*ν*_CN_). IR (solid state): $\tilde{\nu }$ /cm^−1^=3104w, 3070w, 1939 s (*ν*_CO_), 1782 s (*ν*_μ‐CO_), 1558w‐sh, 1536 s (*ν*_CN_), 1456w, 1447w, 1419w, 1394 m, 1360w, 1346w, 1317w, 1251w, 1182w, 1153w, 1116w, 1062 m, 1016w, 1001w, 993 m, 857w‐sh, 838 m, 814 m, 800s, 757 s. ^1^H/^13^C NMR: mixture of isomers in 3 : 2 ratio (^1^H CDCl_3_). Signals attributable to the minor isomer are italicized. ^1^H NMR (CDCl_3_): *δ*/ppm=*6.09*, 5.01 (tt, ^3^ *J*_HH_=12.0, 3.2 Hz, 1H, C2‐H); *4.72*, 4.70 (s, 5H, Cp); 4.64, *4.62* (s, 5H, Cp’); 4.53, *4.05* (s, 3H, C1‐H); *2.59*, 2.37 (d, *J*=12.0 Hz, 1H, C3‐H); 2.28–2.06, 1.98–1.69, 1.42–1.24 (m, 9H, C3‐H’+C4‐H+C5‐H). No change in the ^1^H NMR spectrum was observed after 14 h at room temperature. ^13^C{^1^H} NMR (CDCl_3_): *δ*/ppm=334.8, *334.5* (CN); *268.1*, 267.5 (μ‐CO); 212.5, *211.9* (CO); 86.8, *86.6* (Cp); 86.4, *86.3* (Cp’); *76.6*, 75.4 (C2‐H); *44.4*, 43.6 (C1‐H), 32.5, *32.1* (C3‐H); *32.3*, 30.6, 26.3, 26.2, 26.0, 25.6, 25.6, 25.5 (C3’‐H+C4‐H+C5‐H).

### X‐ray crystallography

Crystal data and collection details for [**2A**]CF_3_SO_3_, [**2C**]Cl ⋅ 2.34H_2_O, [**2C**]CF_3_SO_3_, [**2D**]CF_3_SO_3_ and **6** are reported in Table 7. Data were recorded on a Bruker APEX II diffractometer equipped with a PHOTON2 detector using Mo_Kα_ radiation. The structures were solved by direct methods and refined by full‐matrix least‐squares based on all data using *F* ^2^.^[49]^ Hydrogen atoms were fixed at calculated positions and refined using a riding model, except H(2) in [**2A**]CF_3_SO_3_ which was located in the Fourier map and refined isotropically using the 1.2‐fold *U_iso_* of the parent N(2) atom. The H_2_O molecules of [**2C**]Cl ⋅ 2.34H_2_O are disordered and it was not possible to locate the hydrogen atoms. All non‐hydrogen atoms were refined with anisotropic displacement parameters. The crystals of **6** display a low quality, allowing the full determination of the overall connectivity and geometry of the complex, while the bonding parameters cannot be discussed in detail.

**Table 7:** Crystal data and measurement details for [**2A**]CF_3_SO_3_, [**2C**]Cl ⋅ 2.34H_2_O, [**2C**]CF_3_SO_3_, [**2D**]CF_3_SO_3_ and **6**.

	[**2C**]Cl ⋅ 2.34H_2_O	[**2C**]CF_3_SO_3_	[**2A**]CF_3_SO_3_	[**2D**]CF_3_SO_3_	**6**
Formula	C_21_H_24_ClFe_2_NO_5.34_	C_22_H_24_F_3_Fe_2_NO_6_S	C_24_H_19_F_3_Fe_2_N_2_O_6_S	C_232_H_20_F_3_Fe_2_NO_7_S	C_20_H_24_ClFe_2_NO_2_
FW	523.04	599.18	632.17	623.16	457.55
T, K	100(2)	100(2)	100(2)	100(2)	100(2)
*λ*, Å	0.71073	0.71073	0.71073	0.71073	0.71073
Crystal system	triclinic	orthorhombic	monoclinic	monoclinic	monoclinic
Space group	*P* $\bar{1}$	*Pna*2_1_	P*2_1_/*c	P*2_1_/*c	P*2_1_/n*
*a*, Å	10.8828(6)	21.468(3)	9.3327(8)	9.9264(9)	6.9490(15)
*b*, Å	15.1007(8)	11.9137(18)	20.1635(17)	14.5214(12)	22.695(5)
*c*, Å	15.1537(8)	8.9585(16)	12.8705(10)	17.1821(15)	12.227(2)
*α*,*°*	77.457(3)	90	90	90	90
*β*,°	87.276(3)	90	100.145(4)	102.180(3)	97.775(6)
*γ*,*°*	69.367(3)	90	90	90	90
Cell Volume, Å^3^	2273.8(2)	2291.3(6)	2384.1(3)	2421.0(4)	1910.7(7)
Z	4	4	4	4	4
*D_c_*, g cm^−3^	1.528	1.737	1.761	1.710	1.591
μ, mm^−1^	1.426	1.423	1.374	1.353	1.672
F(000)	1075	1224	1280	1264	944
Crystal size, mm	0.21×0.18×0.15	0.22×0.19×0.12	0.21×0.16×0.13	0.24×0.21×0.12	0.22×0.16×0.13
*θ* limits,°	1.790–26.000	1.897–25.488	1.898–25.050	1.854–25.097	1.795–23.999
Reflections collected	28243	20143	38770	54774	22519
Independent reflections	8885 [*R_int_* =0.0496]	4040 [*R_int_* =0.1419]	4173 [*R_int_* =0.0569]	4267 [*R_int_* =0.0875]	2933 [*R_int_* =0.1406]
Data/restraints /parameters	8885/0/567	4040/193/318	4173/1/347	4267/0/336	2933/264/235
Goodness on fit on F^2^	1.246	1.278	1.243	1.181	1.238
*R*_1_ (*I* >2σ(*I*))	0.0871	0.0939	0.0729	0.0614	0.1692
*wR*_2_ (all data)	0.2056	0.1657	0.1521	0.1426	0.4021
Largest diff. peak and hole, e Å^−3^	0.901/−0.810	1.090/−0.698	0.905/−0.752	1.346/−0.936	2.986/−2.257

### Computational studies

All geometries were optimized with ORCA 4.0.1.2,^[50]^ using the B97 functional in conjunction with a triple‐*ζ* quality basis set (def2‐TZVP). The dispersion corrections were introduced using the Grimme D3‐parametrized correction and the Becke−Johnson damping to the DFT energy.^[51]^ Most of the structures were confirmed to be local energy minima (no imaginary frequencies), but in some case a small, unavoidable negative frequency relative to the Cp rotation around the M−Cp axis was observed. The solvent was considered through the continuum‐like polarizable continuum model (C‐PCM, dichloromethane).

### Behaviour in aqueous media

*Solubility in water*. A suspension of the selected Fe compound (3–5 mg) in a D_2_O solution (0.7 mL) containing Me_2_SO_2_ as internal standard^52^ (3.36 ⋅ 10^−3^ M) was vigorously stirred at 21 °C for 10 h. The resulting saturated solution was filtered over celite, transferred into an NMR tube and analysed by ^1^H NMR spectroscopy (delay time=3 s; number of scans=20). For the most soluble compounds (S≥0.05 M), a saturated solution was prepared in a smaller volume of D_2_O/Me_2_SO_2_ (0.2–0.3 mL), then filtered and diluted with pure D_2_O. The concentration (solubility) was calculated by the relative integral with respect to Me_2_SO_2_ (*δ*/ppm=3.14 (s, 6H)). Results are compiled in Table 3.

*Octanol/water partition coefficients (log* P_*ow*_ *)*. Partition coefficients (*P*_ow_; IUPAC: *K*_D_ partition constant^[53]^), defined as *P*_ow_=c_org_/c_aq_, where c_org_ and c_aq_ are molar concentrations of the selected compound in the organic and aqueous phase, respectively, were determined by the shake‐flask method and UV/Vis measurements.[^16a^, ^54^] Deionized water and octan‐1‐ol were vigorously stirred for 24 h, to enable saturation of both phases, then separated by centrifugation. A stock solution of the selected Fe compound (ca. 2 mg) was prepared by first adding acetone (50 μL, to help solubilization), followed by water‐saturated octanol (2.5 mL). The solution was diluted with water‐saturated octanol (ca. 1 : 3 *v/v* ratio, c_Fe2_ ≈10^−4^ M, so that 1.5≤A≤2.0 at *λ*_max_) and its UV/Vis spectrum was recorded (A0org ). An aliquot of the solution (*V*_org_=1.2 mL) was transferred into a test tube and octanol‐saturated water (*V*_org_=*V*_aq_=1.2 mL) was added. The mixture was vigorously stirred for 30 min at 21 °C then centrifuged (RCF 805, 5 min; Hettich EBA 8G). The UV/Vis spectrum of the organic phase was recorded (Aforg ) and the partition coefficient was calculated as *P*_ow_=Aforg /(A0org_g_−Aforg ) where A0org and Aforg are the absorbance in the organic phase before and after partition with the aqueous phase, respectively.^[53]^ For [**2B**]BF_4_, [**2C**]Cl and [**3F**]CF_3_SO_3_ an inverse procedure was followed, starting from a solution of the compound in octanol‐saturated water. The partition coefficient was calculated as *P*_ow_=(A0aq −Afaq )/Afaq where A0aq and Afaq are the absorbance in the aqueous phase before and after partition with the organic phase, respectively. The wavelength of the maximum absorption of each compound (320–390 nm range) was used for UV/Vis quantitation. The procedure was repeated three times for each sample (from the same stock solution); results are given as mean ± standard deviation (Table 3). Naphthoquinone was used as a reference compound (log *P*=1.8±0.2; literature:^[55]^ 1.71).

*Stability in water (D_2_O) and cell culture medium*. The procedures are described in the Supporting Information, and data are collected in Table S3.

*Carbon monoxide release*. A stock solution was prepared by dissolving the selected Fe compound (ca. 10 mg) in MeOH (0.50 mL) then diluting with H_2_O up to 10.0 mL total volume (5 % *v/v* MeOH). Next, 3.20 mL of the solution (*n*_Fe_ ca. 6 ⋅ 10^−3^ mmol) and a 7x2 mm stir bar were transferred into a 4‐mL screw top vial (1.72 mL neat gas phase volume). The mixture was sealed with a screw cap with a PTFE/silicone septum and heated at 37 °C for 24 h under stirring. Afterwards, the headspace was sampled with a gas tight microsyringe (250 mL) and analysed by GC‐TCD. Therefore, the vial was vented, sealed and heated for further 24 h, and GC‐TCD analysis was repeated. The amount of released CO (ν_CO_, mmol) was calculated based on a calibration curve obtained from analyses of known air/CO mixtures (1–10 % *v/v*), and the number of equivalents released over a 24 h period refers to the initial amount of compound (eq_CO_=*n*_CO_/*n*_Fe_). The residual amount of starting diiron complex after 48 h was calculated by assuming the release of 3 equivalents of CO per mole and the total amount of CO released. The procedure was repeated three times for each sample (from the same stock solution); results are given as mean ± standard deviation (Table S4).

### Reactivity with model protein

Lysozyme from chicken egg white (M=14 300 Da, 62971 Sigma‐Aldrich) was stored at 4 °C as received. A stock solution was prepared by dissolving the powder protein (125 μM) and NH_4_OAc (1.25×10^−2^ M)^[56]^ in HPLC water and was immediately used. Stock solutions of diiron complexes (*c* ${}_{\mathrm{Fe}{}_{2}}$ =1×10^−3^ M) were prepared by dissolving the selected compound in DMSO (0.25 mL) or MeOH (0.7 mL; used only for [**4G**]BF_4_), then diluting with HPLC water up to 5.0 mL total volume. Next, aliquots of the Fe (0.50 mL) and lysozyme (2.0 mL) stock solutions were mixed and the final solution (Fe_2_ 200 μM, lysozyme 100 μM, NH_4_OAc 10 mM; 1 % DMSO) was kept at 37 °C for 24 h. The resulting suspensions were centrifuged (RCF 805, 5 min; Hettich EBA 8G) to separate the orange‐brown precipitate. A blank solution (lysozyme only) was prepared and treated by the same procedure. Next, samples were diluted 1 : 20 (*v/v*) with a water/MeCN (1 : 1, *v/v*) solution containing 1 % formic acid and analysed by HPLC‐MS and flow injection MS. For HPLC analyses, elution was conducted with a MeCN/water linear gradient (90 : 10 to 5 : 95 *v/v* over 60 min), containing 0.05 % trifluoroacetic acid. The following compounds were identified by their MS pattern: [**2A**]^+^ (*t*_R_=18.0 min), [**2B**]^+^ (*t*_R_=13.8 min), [**2C**]^+^ (*t*_R_=18.8 min), [**2D**]^+^ (*t*_R_=17.0 min), [**2E**]^+^ (*t*_R_=18.3 min), [**2F**]^+^ (*t*_R_=9.7 min), [**3F**]^+^ (*t*_R_=13.1 min), [**4G**]^+^ (*t*_R_=23.0 min), [**5H**]^+^ (*t*_R_=24.3 min), lysozyme‐CH_3_ (*t*_R_=21.0 min), lysozyme (*t*_R_=21.6 min). In all cases, the starting organometallic cation was identified as the major Fe‐containing compound in solution. Protein peaks pattern were obtained following peak reconstruction. Peak integrals for lysozyme and methyl lysozyme were calculated from the extracted ion chromatograms from the flow injection MS analysis (Table 4). MS patterns are displayed in Figures S33–S41.

### Biological studies

Compounds [**2B**]BF_4_, [**2C**]CF_3_SO_3_, [**2D**]CF_3_SO_3_ and [**3F**]CF_3_SO_3_ were dissolved in water while the other compounds were dissolved in the minimum DMSO amount prior to cell culture testing. A calculated amount of the stock drug DMSO solution was added to the cell culture media to reach a final maximum DMSO concentration of 0.5 %, which had no effects on cell viability.

Cisplatin was dissolved in 0.9 % sodium chloride solution. MTT [3‐(4,5‐dimethylthiazol‐2‐yl)‐2,5‐diphenyltetrazolium bromide], cisplatin and ImmunoPure *p*‐nitrophenyl phosphate (APH) were obtained from Sigma‐Aldrich.

*Cell cultures*. Human colon (HCT‐15), pancreatic (PSN‐1) and breast (MCF‐7) carcinoma cell lines along with human melanoma cells (A375) were obtained from American Type Culture Collection (ATCC, Rockville, MD). Embryonic kidney HEK293 cells were obtained from the European Collection of Cell Cultures (ECACC, Salisbury, UK). Human ovarian 2008 cancer cells were kindly provided by Prof. G. Marverti (Dept. of Biomedical Science of Modena University, Italy). MCF‐7 ADR cells were kindly provided by Prof. N. Colabufo (Dept. Pharmacy, University of Bari, Italy). Cell lines were maintained in the logarithmic phase at 37 °C in a 5 % CO_2_ atmosphere using the following culture media containing 10 % fetal calf serum (Euroclone, Milan, Italy), antibiotics (50 units/mL penicillin and 50 μg/mL streptomycin) and 2 mM l‐glutamine: i) RPMI‐1640 medium (Euroclone, Milan, Italy) for PSN‐1, 2008, HCT‐15, MCF‐7 and MCF‐7 ADR cells; ii) DMEM for A375 and HEK293 cells.

*Spheroid cultures*. Spheroid cultures were obtained by seeding 2.5×10^3^ cells/well in round bottom non‐tissue culture treated 96 well‐plate (Greiner Bio‐one, Kremsmünster, Austria) in phenol red free RPMI‐1640 medium (Sigma Chemical Co.), containing 10 % FCS and supplemented with 20 % methyl cellulose stock solution.

*MTT assay*. The growth inhibitory effect towards tumour cells was evaluated by means of MTT assay as previously described.^[57]^ Briefly, 3–8×10^3^ cells/well, dependent upon the growth characteristics of the cell line, were seeded in 96‐well microplates in growth medium (100 μL). After 24 h, the medium was removed and replaced with a fresh one containing the compound to be studied at the appropriate concentration. Triplicate cultures were established for each treatment. After 24 or 72 h, each well was treated with 10 μL of a 5 mg/mL MTT saline solution, and following 5 h of incubation, 100 μL of a sodium dodecyl sulfate (SDS) solution in HCl 0.01 M were added. After an overnight incubation, cell growth inhibition was detected by measuring the absorbance of each well at 570 nm using a Bio‐Rad 680 microplate reader. Mean absorbance for each drug dose was expressed as a percentage of the control untreated well absorbance and plotted against drug concentration. IC_50_ values, the drug concentrations that reduce the mean absorbance at 570 nm to 50 % of those in the untreated control wells, were calculated by the four‐parameter logistic (4‐PL) model. Evaluation was based on means from at least four independent experiments.

*Acid phosphatase (APH) assay*. An APH modified assay was used for determining cell viability in 3D spheroids, as previously described.^[58]^ IC_50_ values were calculated with a four‐parameter logistic (4‐PL) model. All the values are the means ± SD of not less than four independent experiments.

*Mitochondrial membrane potential (Δ*Ψ*)*. The Δ*Ψ* was assayed using the Mito‐ID® Membrane Potential Kit according to the manufacturer's instructions (Enzo Life Sciences, Farmingdale, NY) as previously described.^[59]^ Briefly, PSN‐1 cells (8×10^3^ per well) were seeded in 96‐well plates; after 24 h, cells were washed with PBS and loaded with Mito‐ID detection reagent for 30 min at 37 °C in the dark. Afterwards, cells were incubated with increasing concentrations of tested complexes. Fluorescence intensity was estimated using a VICTOR X3 (PerkinElmer, USA) plate reader at *λ*_ex_=490 and *λ*_em_=590 nm. Antimycin (3 μM) was used as positive control.

*ROS production*. The production of ROS was measured in PSN‐1 cells (10^4^ per well) grown for 24 h in a 96‐well plate in RPMI medium without phenol red (Merck). Cells were then washed with PBS and loaded with 10 μM 5‐(and‐6)‐chloromethyl‐2′,7′‐dichlorodihydrofluorescein diacetate acetyl ester (CM‐H_2_DCFDA; Molecular Probes‐Invitrogen) for 25 min, in the dark. Afterwards, cells were washed with PBS and incubated with increasing concentrations of tested compounds. Fluorescence increase was estimated by using *λ*_ex_=485 nm and *λ*_em_=527 nm in a VICTOR X3 (PerkinElmer) plate reader. Antimycin (3 μM, Merck), a potent inhibitor of Complex III in the electron transport chain, was used as positive control.

*Intracellular CO release*. PSN‐1 cell (1×10^4^) were seeded in 96‐well plates. After 24 h, cells were treated with 20 μM of tested complexes or CORM‐401 (Merck) for 15 min at 37 °C and followed by 30 min incubation with a fluorescent probe BioTracker Carbon Monoxide Probe 1 Live Cell Dye® (Merck). The intracellular fluorescence, indicative of CO accumulation in cells, was quantified using a VICTOR X3 (PerkinElmer) plate reader.

*Cellular uptake*. PSN‐1 and HEK293 cells (3×10^6^) were seeded in 75 cm^2^ flasks in growth medium (20 mL). After overnight incubation, the medium was replaced and the cells were treated with tested compounds for 24 h. Cell monolayers were washed twice with cold PBS, harvested and counted. Samples were than subjected to three freezing/thawing cycles at −80 °C, and then vigorously vortexed. The samples were treated with highly pure nitric acid (Fe:≤0.01 μg kg^−1^, TraceSELECT ® Ultra, Sigma) and transferred into a microwave Teflon vessel. Subsequently, samples were submitted to standard procedures using a speed wave MWS‐3 Berghof instrument (Eningen, Germany). After cooling, each mineralized sample was analysed for iron using a Varian AA Duo graphite furnace atomic absorption spectrometer (Varian) at the wavelength of 248.3 nm. The calibration curve was obtained using known concentrations of standard solutions purchased from Sigma Chemical Co.

*Statistical analysis*. All values are the means ± SD of no less than three measurements starting from three different cell cultures. Multiple comparisons were made by ANOVA followed by the Tukey‐Kramer multiple comparison test (* *p*<0.05, ** *p*<0.01), using GraphPad software.

## Supporting Information

IR and NMR spectra of products and comparison of selected spectroscopic data; DTF optimized structures; stability studies in water and cell culture media; carbon monoxide release studies; IC_50_ values after 24 h of incubation; MS spectra from lysozyme interaction experiments. Deposition numbers 2045779 (for [**2A**]CF_3_SO_3_), 2045778 (for [**2C**]CF_3_SO_3_), 2045777 (for [**2C**]Cl), 2045780 (for [**2D**]CF_3_SO_3_) and 2045781 (for **6**) contain the supplementary crystallographic data for this paper. These data are provided free of charge by the joint Cambridge Crystallographic Data Centre and Fachinformationszentrum Karlsruhe Access Structures service.

## Conflict of interest

The authors declare no conflict of interest.

## Supporting information

As a service to our authors and readers, this journal provides supporting information supplied by the authors. Such materials are peer reviewed and may be re‐organized for online delivery, but are not copy‐edited or typeset. Technical support issues arising from supporting information (other than missing files) should be addressed to the authors.

Supplementary
