# Supplementary material for: Easily Available, Amphiphilic Diiron Cyclopentadienyl Complexes Exhibit in Vitro Anticancer Activity in 2D and 3D Human Cancer Cells through Redox Modulation Triggered by CO Release
Source: Chemistry. 2021 Jun 9;27(39):10169–85. doi: 10.1002/chem.202101048 (PMC8362065; doi:10.1002/chem.202101048)
Supplement: Supplementary file 1 — Supplementary [file CHEM-27-10169-s001.pdf]

Chemistry–A European Journal

Supporting Information

Easily Available, Amphiphilic Diiron Cyclopentadienyl Complexes Exhibit in Vitro Anticancer Activity in 2D and 3D Human Cancer Cells through Redox Modulation Triggered by CO Release

Lorenzo Biancalana, Michele De Franco, Gianluca Ciancaleoni, Stefano Zacchini, Guido Pampaloni, Valentina Gandin,* and Fabio Marchetti*

Supporting Information

**Table of contents**	**Pages**
1. Synthesis, X-Ray and DFT characterization (**Scheme S1, Figures S1-S2, Table S1**)	S2-S4
2. IR and NMR spectra and selected spectroscopic data (**Table S2; Figures S3-S32**).	S5-S24
3. DFT-optimized geometries (data)	S25-S30
4. Stability studies in aqueous media (**Scheme S2**; **Tables S3-S4**).	S31-S36
5. IC_{50} values of complexes on PSN-1 cells after 24 hours (**Table S5**).	S37
6. Lysozyme interaction studies (**Figures S33-S41**).	S38-S40
7. Notes and references.	S41

1. Synthesis, X-Ray and DFT characterization.

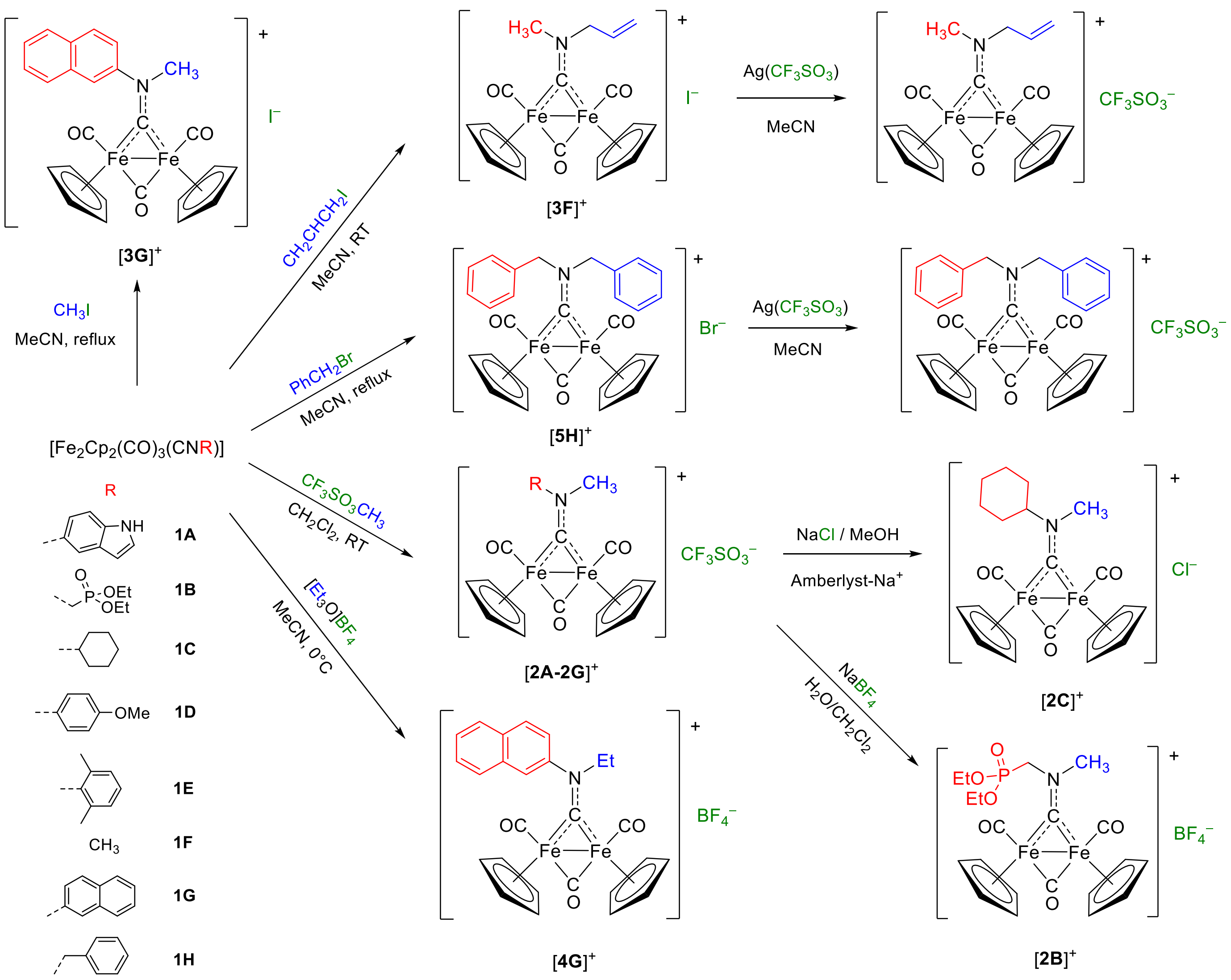

Scheme S1. Synthetic routes for the preparation of diiron bis-cyclopentadienyl aminocarbyne complexes from $[Fe_2Cp_2(CO)_4]$, employing different isocyanides, alkylating agents and anion exchange procedures. RT = room temperature.

Figure S1, Table S1. DFT-optimized geometries of selected diiron complexes, and relevant calculated bond distances (Å).

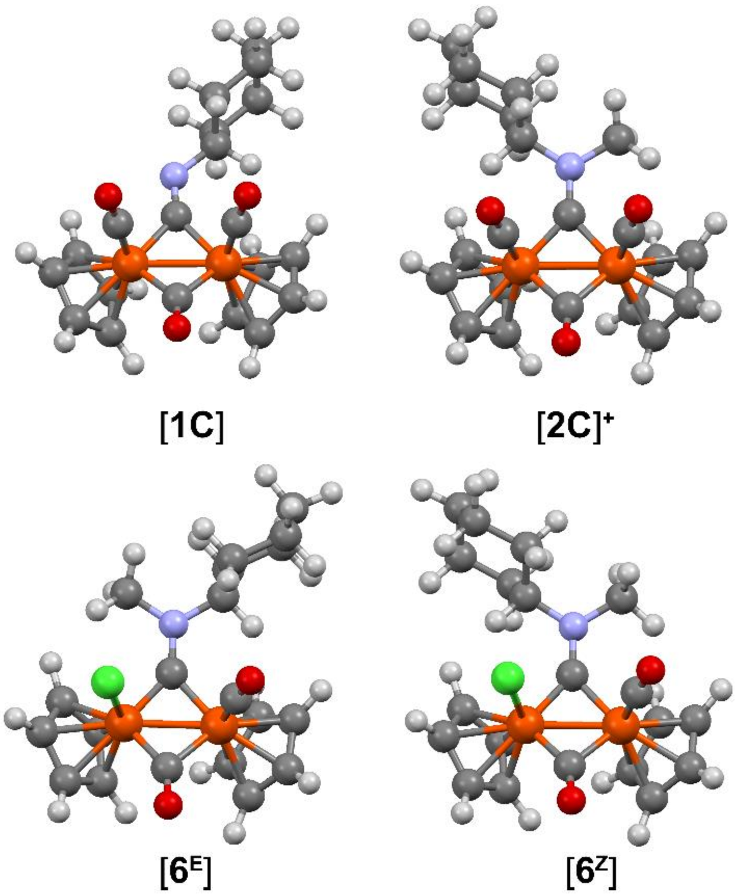

	**1C**	**[2C]**$^{+}$	**6**^{E}	**6**^{Z}
Fe-Fe	2.522	2.509	2.489	2.488
(CO)Fe-$C_{carbyne}$	1.943	1.870	1.875	1.872
(Cl)Fe-$C_{carbyne}$	=	=	1.827	1.832
$C_{carbyne}$-N	1.233	1.296	1.304	1.304
μ-C-O	1.188	1.178	1.188	1.187
t-C-O	1.164	1.156	1.162	1.162

Figure S2. Molecular structure of **6**. Displacement ellipsoids are at the 30% probability level. H-atoms have been omitted for clarity.

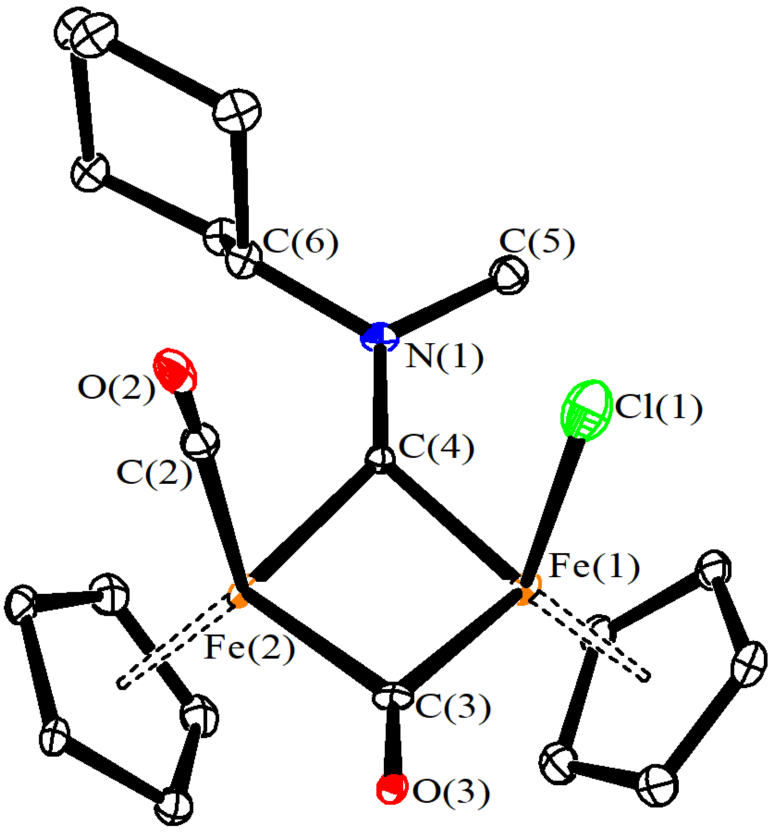

2. IR and NMR spectra and selected spectroscopic data.

Table S2. Selected IR and NMR data for $[Fe_2Cp_2(CO)_2(\mu\text{-}CO)\{\mu\text{-}CNRR'\}]^+$ and $[Fe_2Cp_2Cl(CO)(\mu\text{-}CO)\{\mu\text{-}CNMe(C_6H_{11})\}]^+$ (**6**) complexes.

Compound	R	R'	IR (CH_2Cl_2): ũ / cm^{-1}			^{13}C NMR ($CDCl_3$): δ / ppm				^{1}H NMR ($CDCl_3$): δ / ppm		
			ν(t-CO)	ν(μ-CO)	ν(μ-CN)	t-CO	μ-CO	μ-CN	Cp	Cp	NMe	NCH
[**2A**]CF_3SO_3 [a]	Me	Ind	2023s 1991m-sh	1836s	1542m	209.8, 209.2	255.9	324.9	91.2, 91.0	5.39, 4.65	4.53	
[**2B**]BF_4	Me	Phos	2028s 1996w	1836m	1574w	207.4, 207.1	254.7	323.7	90.3, 90.1	5.29	4.35	5.15, 4.93 (CH_2P)
[**2C**]CF_3SO_3	Me	Cy	2020s 1988w-sh	1835m	1567w	208.5, 207.6	255.3	316.4	90.3, 90.1	5.36, 5.27	4.06	4.68 (C_6H_{11})
[**2C**]Cl	Me	Cy	2018s 1985w-sh	1833m	1569w	208.7, 207.8	255.8	316.2	90.7, 90.3	5.51, 5.39	4.14	4.70 (C_6H_{11})
[**2D**]CF_3SO_3 [c]	Me	*p*Anis	2021s 1989w	1836m	1540w	208.6, 207.6	254.5	324.3	90.3, 90.0	5.47, 4.82	4.57	
[**2E**]CF_3SO_3 [c]	Me	Xyl	2023s 1992w	1840m	1530w	208.6	253.9	327.8	91.3, 91.1	5.48, 4.83	4.44	
[**2F**]CF_3SO_3 [c]	Me	Me	2022s 1990w	1853m	1602w-m	209.3	257.6	315.5	90.8	5.32	4.29	
[**3F**]CF_3SO_3	Me	All	2022s 1990w	1836m	1584m	208.1, 207.8	255.3	318.1	90.*20*, 90.*16*	5.34, 5.32	4.17	5.26, 5.12 (CH_2)
[**4G**]BF_4	Et	Naph	2021s 1989w	1836m	1538w	209.2, 208.4	254.9	323.2	90.6, 90.3	5.44, 4.65	-	5.12, 4.95 (CH_2Me)
[**5H**]CF_3SO_3	Bn	Bn	2023s 1991m	1840m	1550w	208.2	253.7	324.4	90.6	5.40	-	5.66, 5.50 (CH_2Ph)
**6** [c]	Me	Cy	1977s	1798s	1537m	212.5, *211.9*	267.5, *268.1*	334.8, *334.5*	86.8, *86.6* 86.4, *86.3*	4.70, *4.72* 4.64, *4.62*	4.53, *4.05*	5.01, *6.09* (C_6H_{11})

Abbreviation list. Ind = 1H-indol-6-yl; Phos = $CH_2P(O)(OEt)_2$; Cy = C6H11; Xyl = 2,6-$C_6H_3Me_2$; All = CH_2CHCH_2; Naph = 2-naphthyl; Bn = CH_2Ph. [a] NMR data in CD_3CN. [b] [**2C**]$^+$ ^1H NMR signals in CD_3OD: 5.40, 5.36, 4.07; D_2O: 5.37, 5.35, 4.08 (identical for both salts). [c] ^{13}C NMR data taken from the literature (DMSO-d_6 for [**2F**]CF_3SO_3).[1,2] [c] NMR data given as major isomer, *minor isomer*.

Solid-state IR spectra of diiron compounds.

Figure S3. Solid-state IR spectrum (650-4000 cm^{-1}) of $[Fe_2Cp_2(CO)_2(\mu\text{-}CO)\{\mu\text{-}CNMe(1H\text{-}indol\text{-}6\text{-}yl)\}]CF_3SO_3$, [**2A**]$CF_3SO_3$.

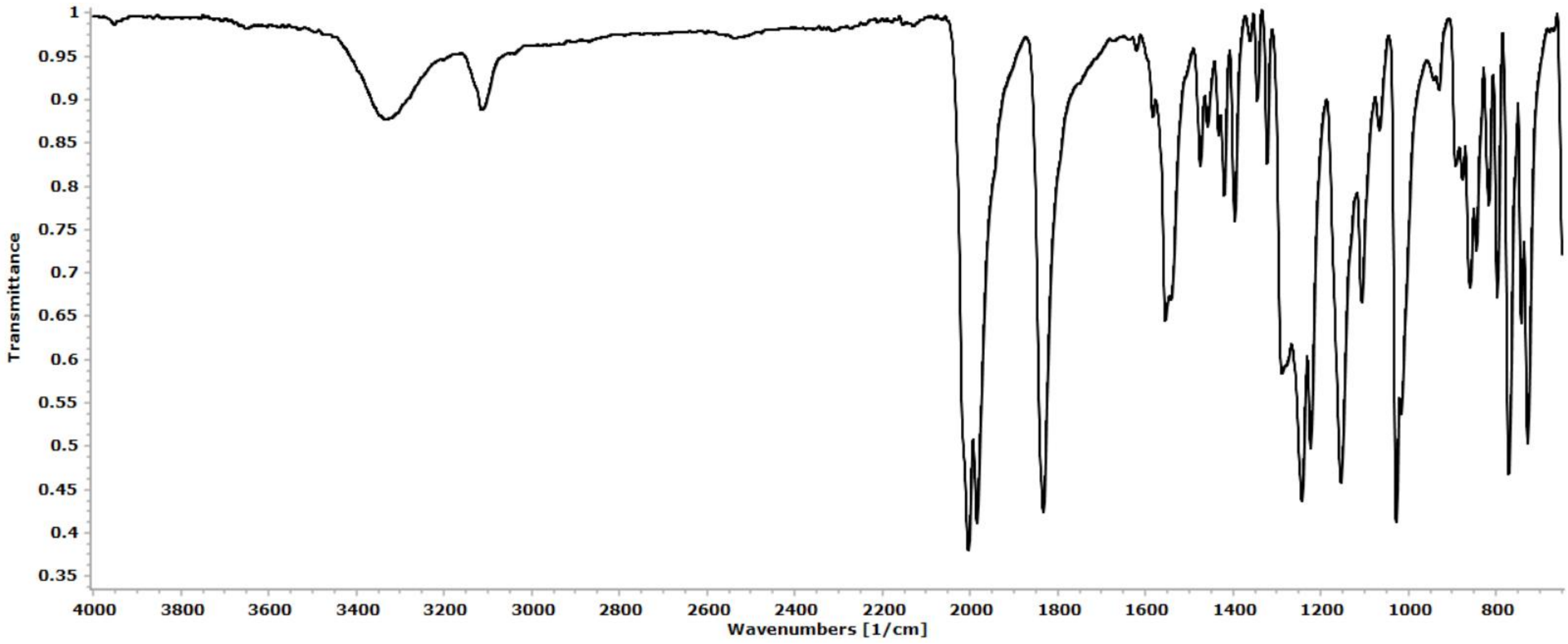

Figure S4. Solid-state IR spectrum (650-4000 cm^{-1}) of $[Fe_2Cp_2(CO)_2(\mu\text{-}CO)\{\mu\text{-}CNMe(CH_2PO_3Et_2)\}]BF_4$, [**2B**]$BF_4$.

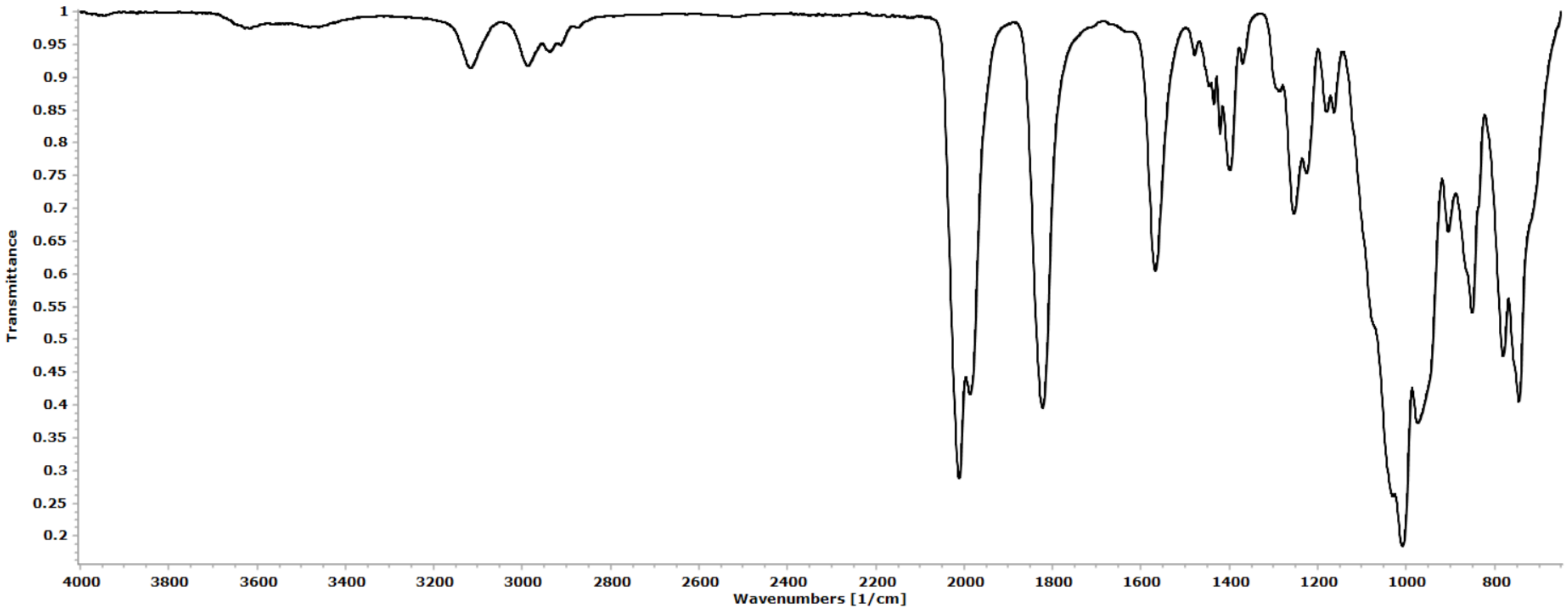

Figure S5. Solid-state IR spectra (650-4000 cm^{-1}) of $[Fe_2Cp_2(CO)_2(\mu\text{-}CO)\{\mu\text{-}CNMe(C_6H_{11})\}]^+$, [**2C**]$^+$, as $CF_3SO_3^-$ (blue line) or Cl^- salt. The broad absorption at 3500 cm^{-1} is due to moisture.

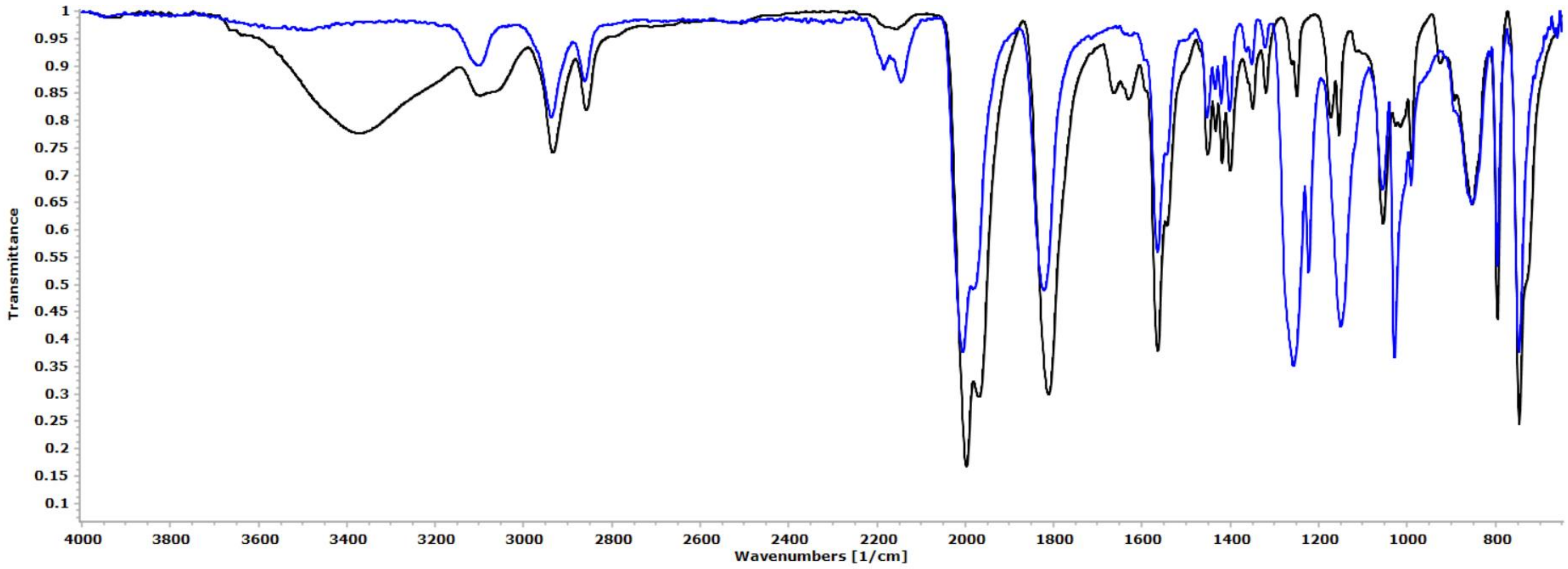

Figure S6. Solid-state IR spectrum (650-4000 cm^{-1}) of [$Fe_2Cp_2(CO)_2$(μ-CO){μ-CNMe(4-C_6H_4OMe)}]CF_3SO_3, [**2D**]CF_3SO_3.

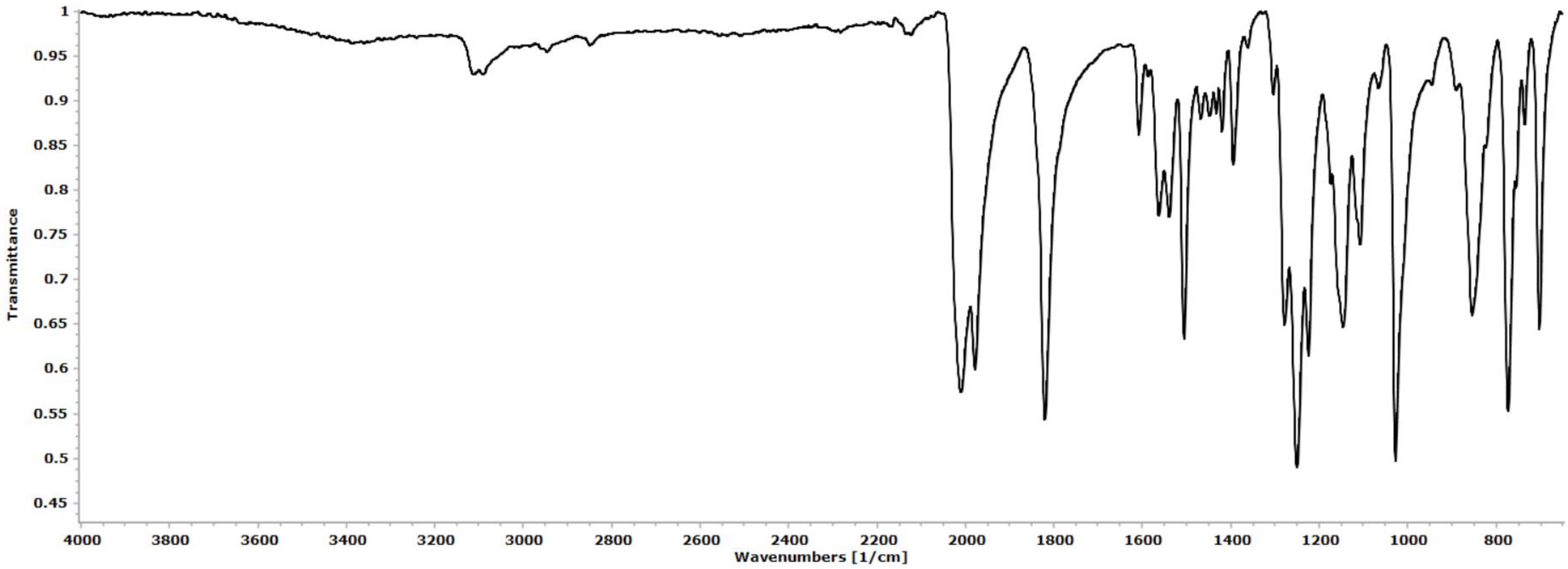

Figure S7. Solid-state IR spectrum (650-4000 cm^{-1}) of [$Fe_2Cp_2(CO)_2$(μ-CO){μ-CNMe(CH_2CHCH_2)}] CF_3SO_3, [**3F**]CF_3SO_3.

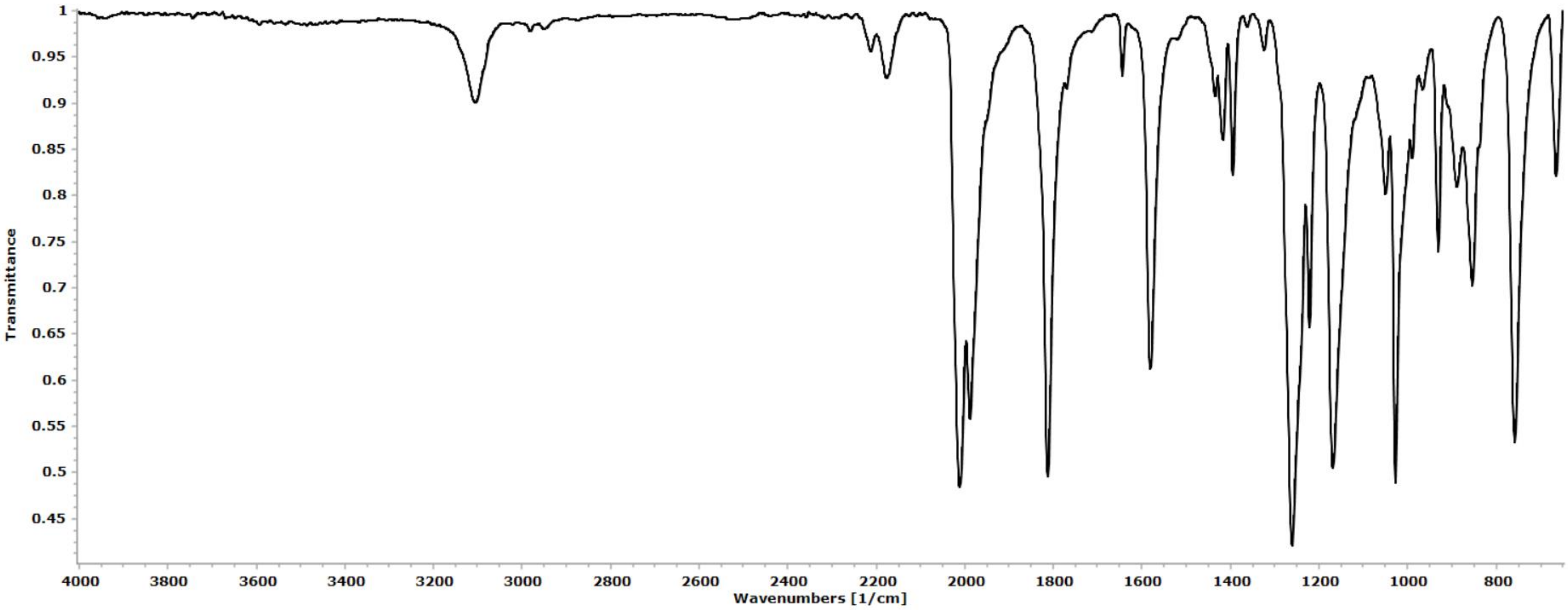

Figure S8. Solid-state IR spectrum (650-4000 cm^{-1}) of [$Fe_2Cp_2(CO)_2$(μ-CO){μ-CNEt(2-naphthyl)}]BF_4, [**4G**]BF_4.

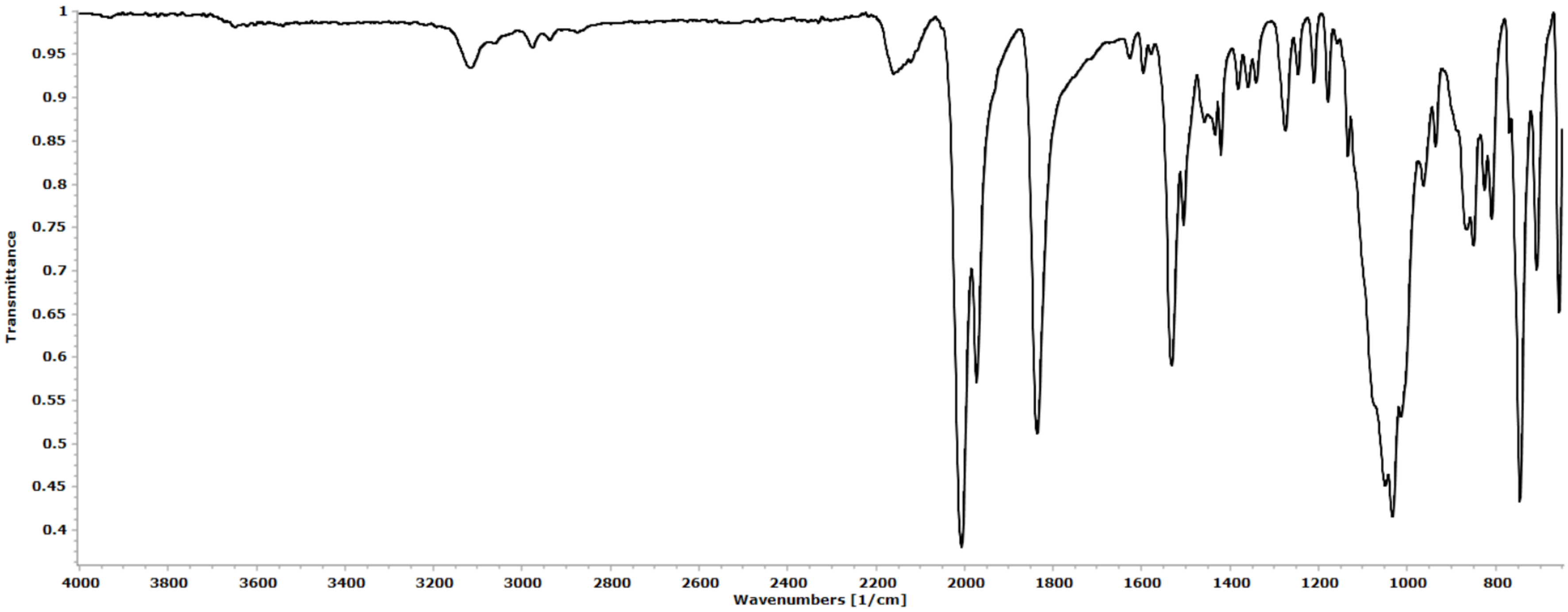

Figure S9. Solid-state IR spectrum (650-4000 cm^{-1}) of $[Fe_2Cp_2(CO)_2(\mu\text{-}CO)\{\mu\text{-}CNBn_2\}]CF_3SO_3$, [**5H**]$CF_3SO_3$.

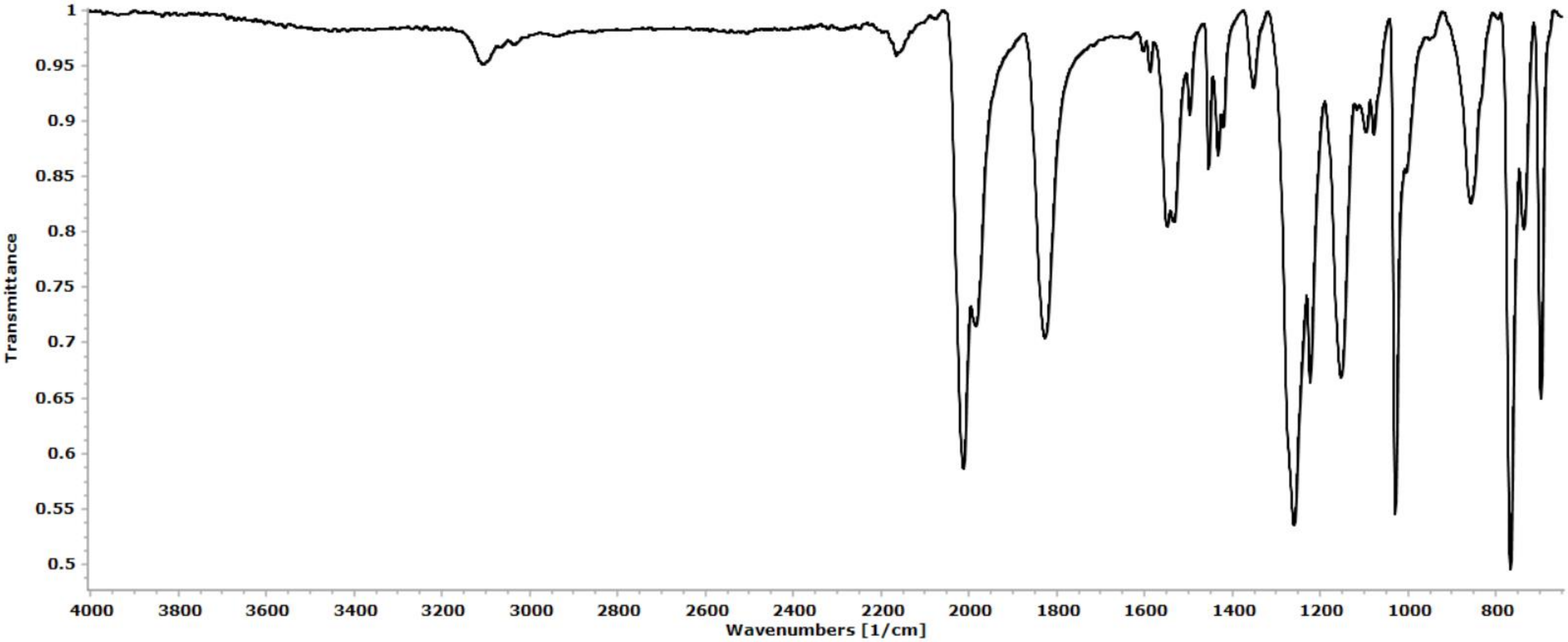

Figure S10. Solid-state IR spectrum (650-4000 cm^{-1}) of [$Fe_2Cp_2Cl(CO)(\mu\text{-}CO)\{\mu\text{-}CNMe(C_6H_{11})\}$], **6.**

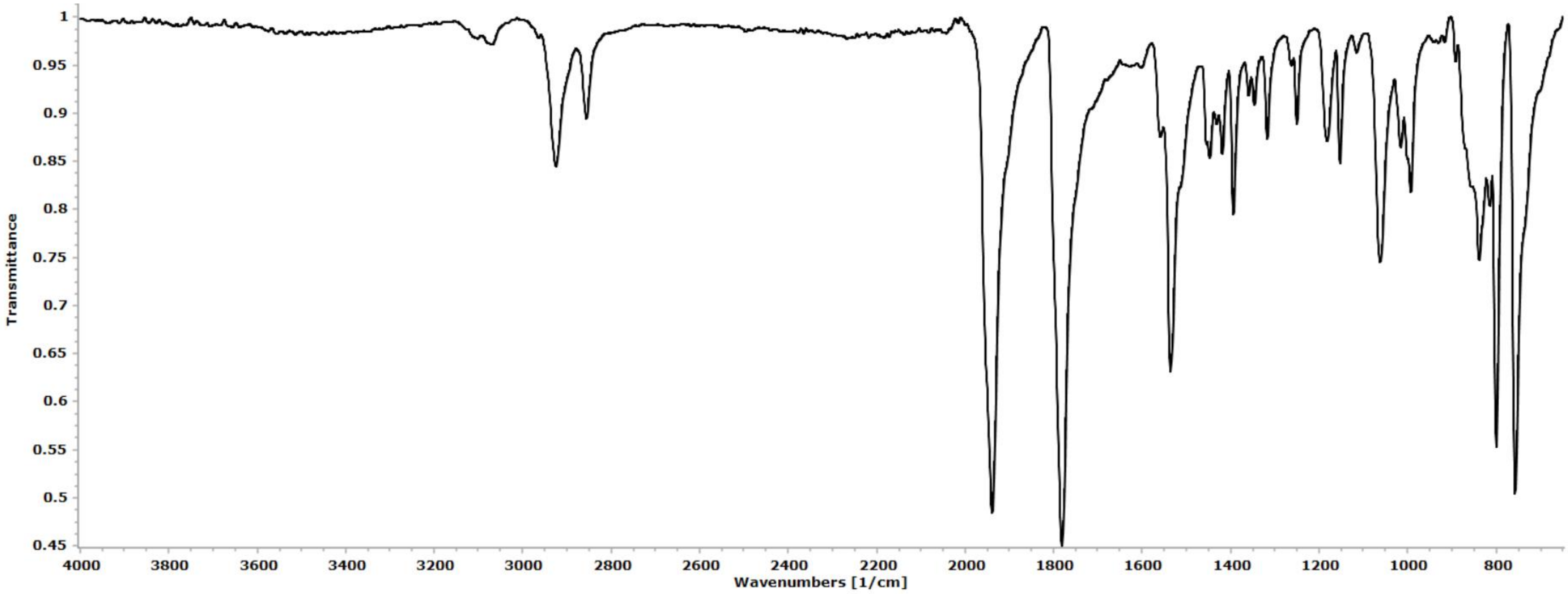

Solution NMR spectra of diiron compounds.

Figure S11. ^{1}H NMR spectrum (401 MHz, CD_3CN) of [**2A**]CF_3SO_3.

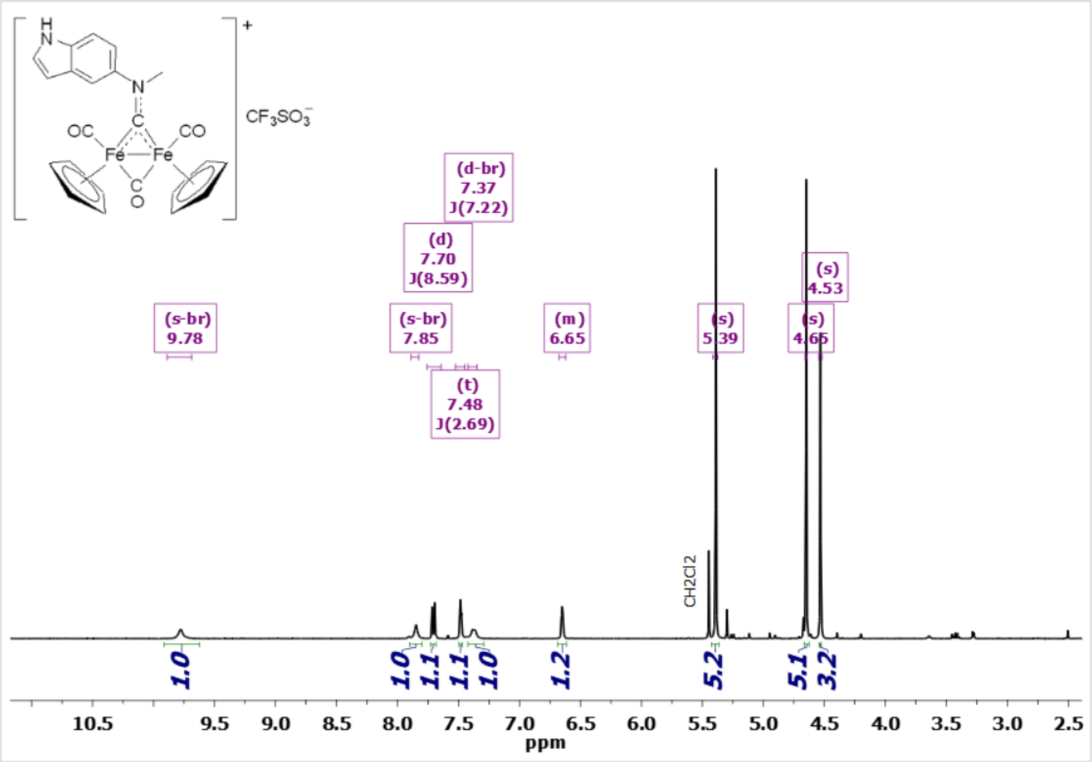

Figure S12. $^{13}C\{^1H\}$ NMR spectrum (101 MHz, CD_3CN) of [**2A**]CF_3SO_3.

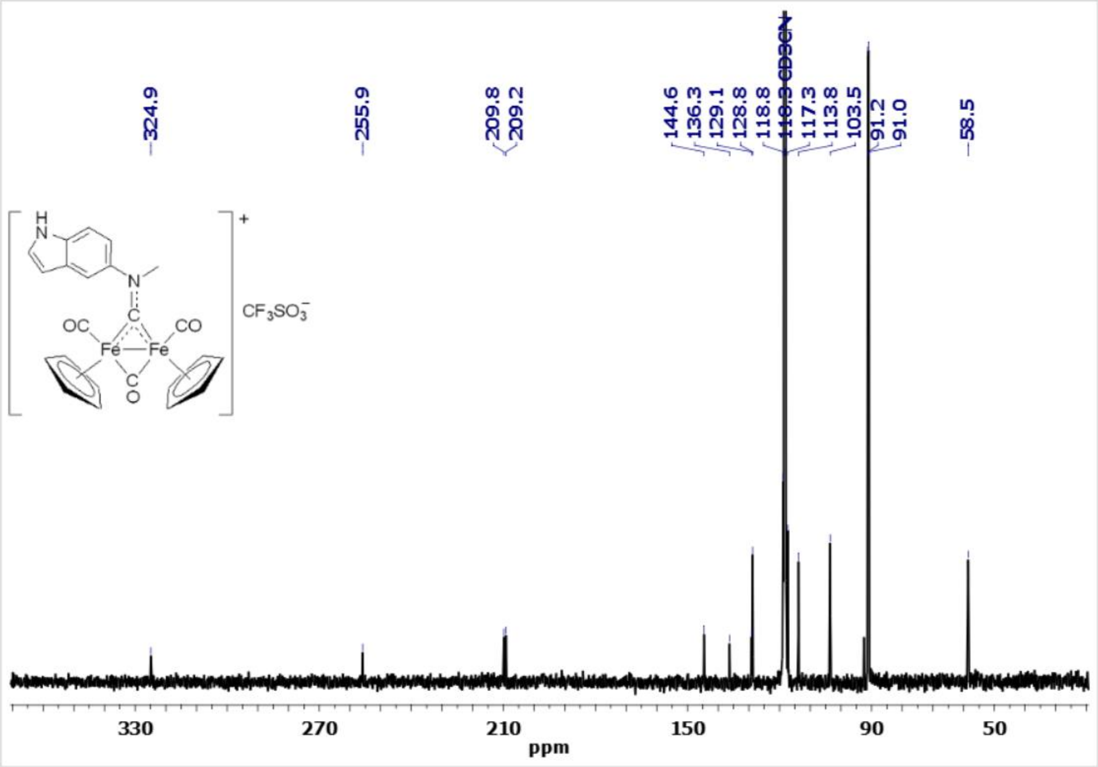

Figure S13. ^{1}H NMR spectrum (401 MHz, $CDCl_3$) of [**2B**]BF_4.

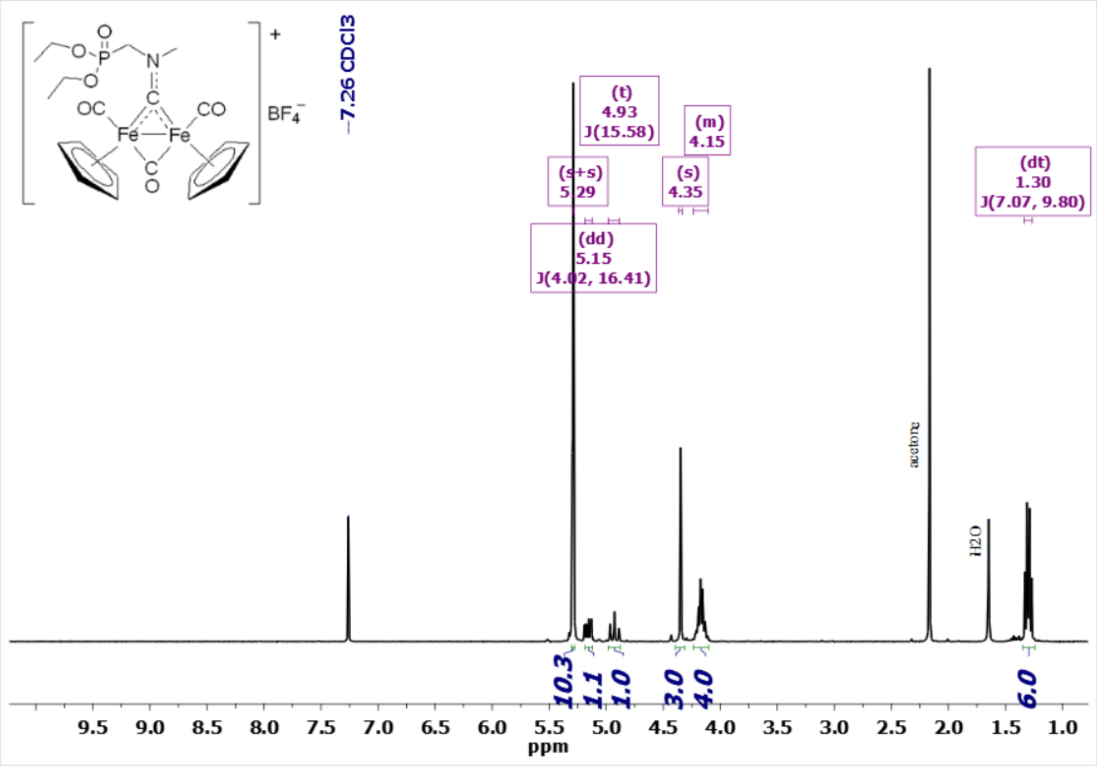

Figure S14. $^{13}C\{^{1}H\}$ NMR spectrum (101 MHz, $CDCl_3$) of [**2B**]BF_4.

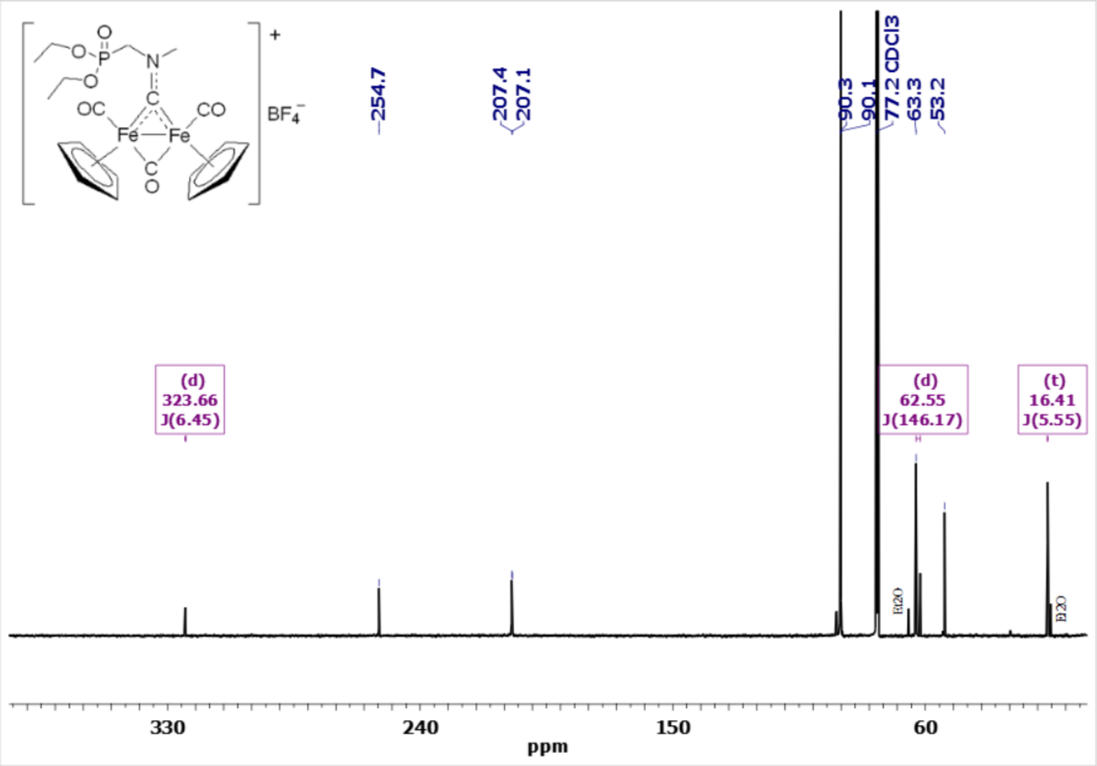

Figure S15. $^{31}P\{^1H\}$ NMR spectrum (162 MHz, $CDCl_3$) of [**2B**]BF_4.

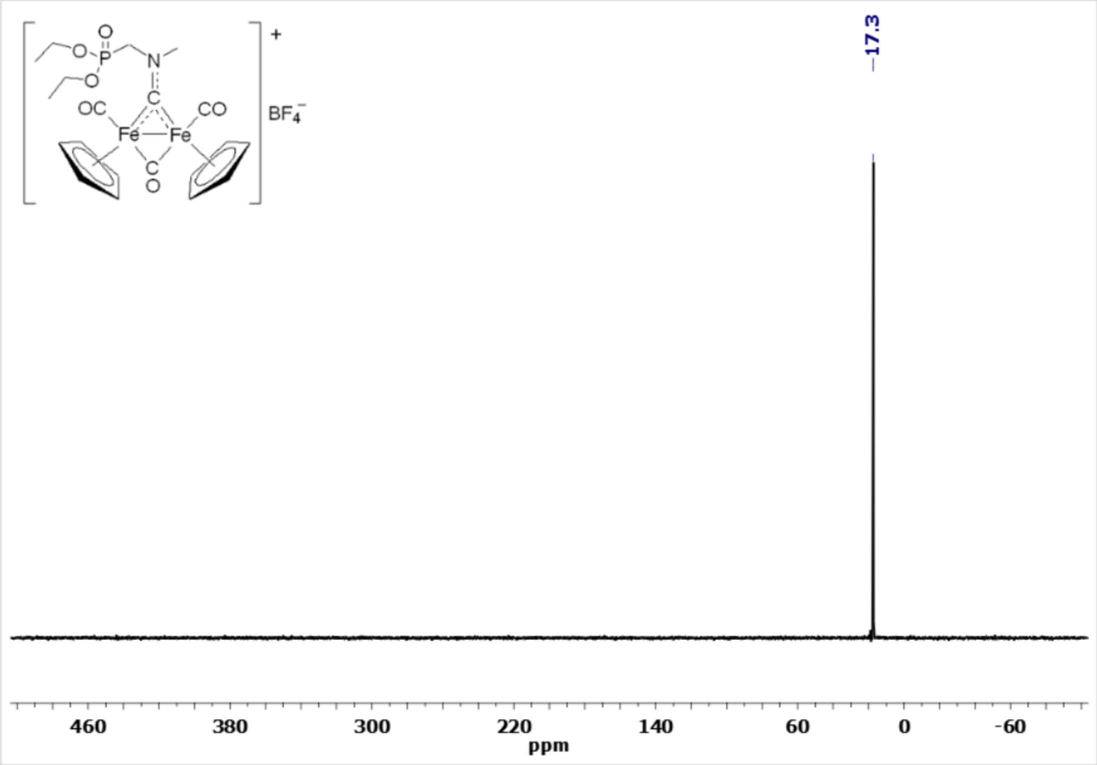

Figure S16. $^{19}F\{^1H\}$ NMR spectrum (378 MHz, $CDCl_3$) of [**2B**]BF_4.

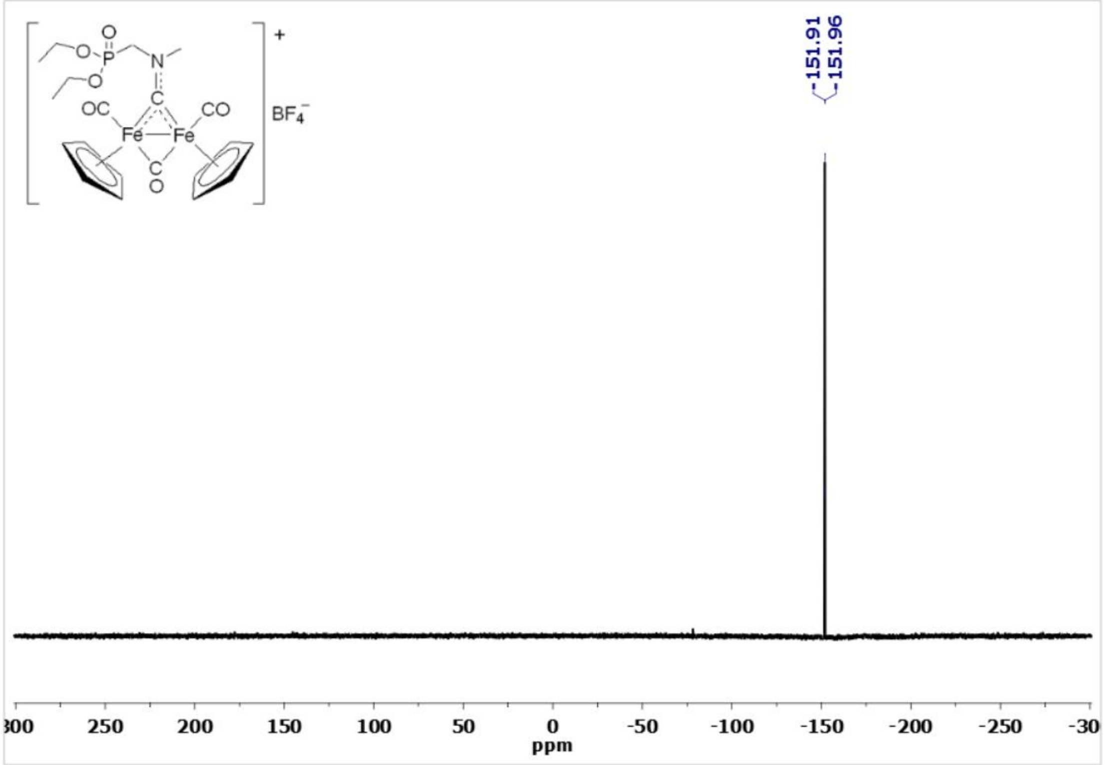

Figure S17. ^{1}H NMR spectrum (401 MHz, $CDCl_3$) of [**2C**]CF_3SO_3.

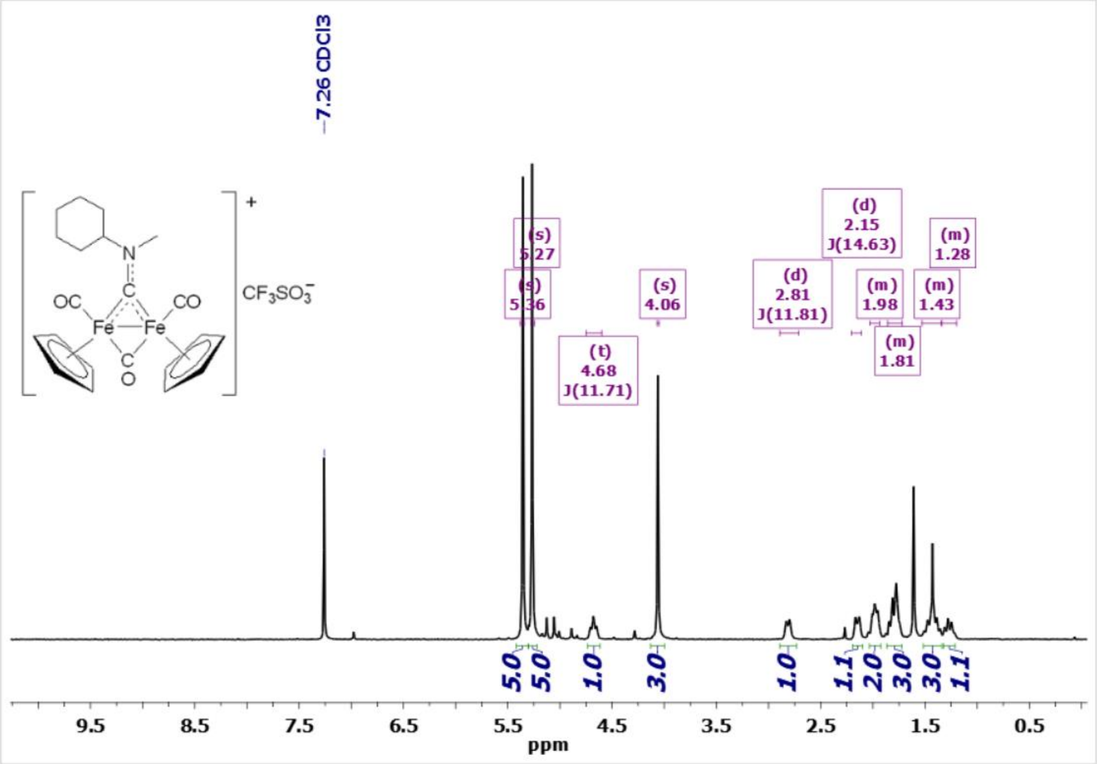

Figure S18. $^{13}C\{^{1}H\}$ NMR spectrum (101 MHz, $CDCl_3$) of [**2C**]CF_3SO_3.

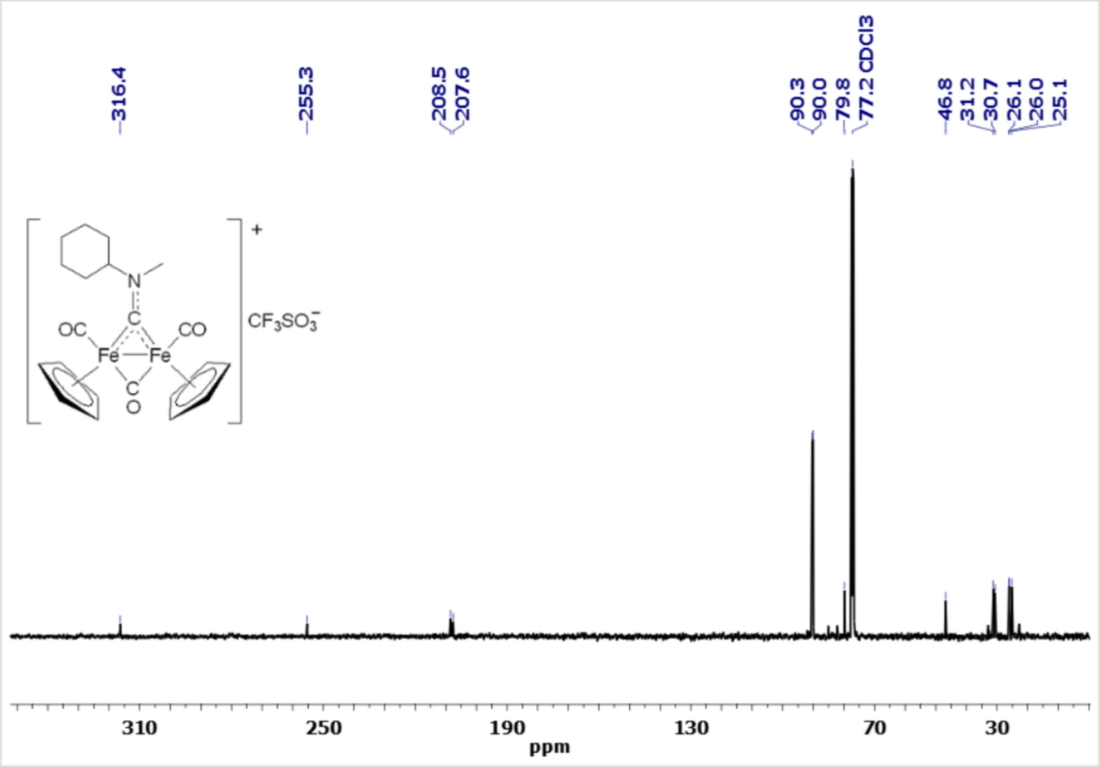

Figure S19. ^{1}H NMR spectrum (401 MHz, $CDCl_3$) of [**2C**]Cl.

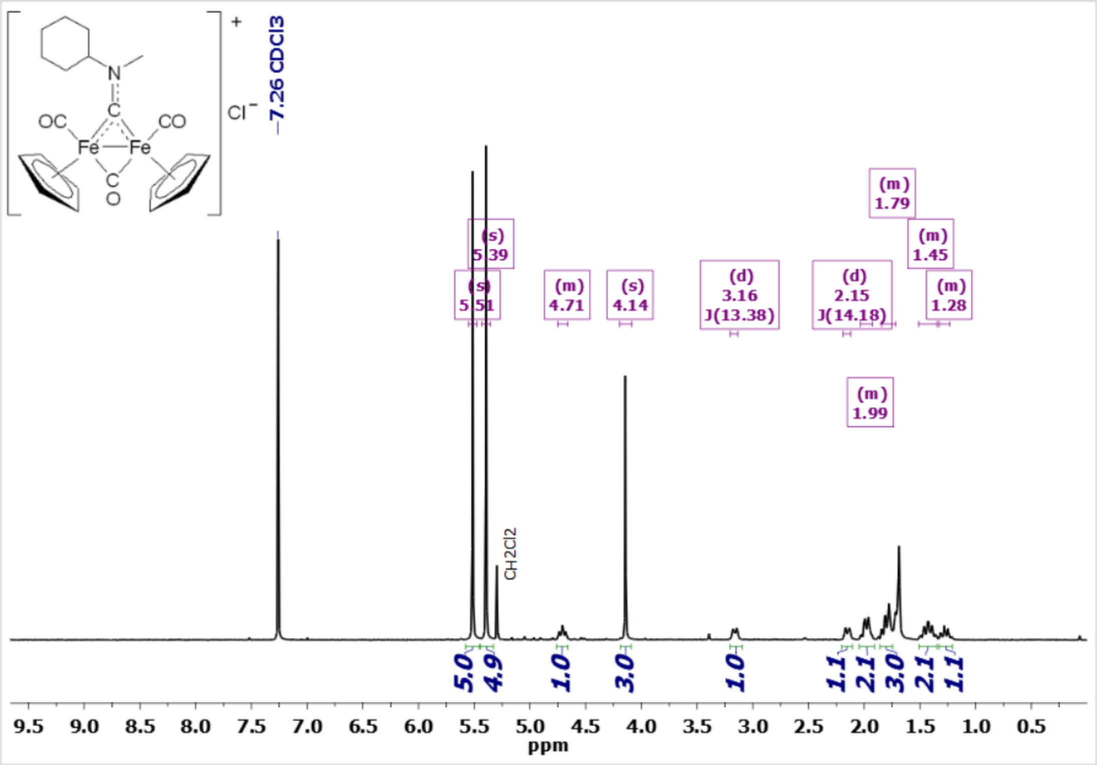

Figure S20. ^{13}C{^{1}H} NMR spectrum (101 MHz, $CDCl_3$) of [**2C**]Cl.

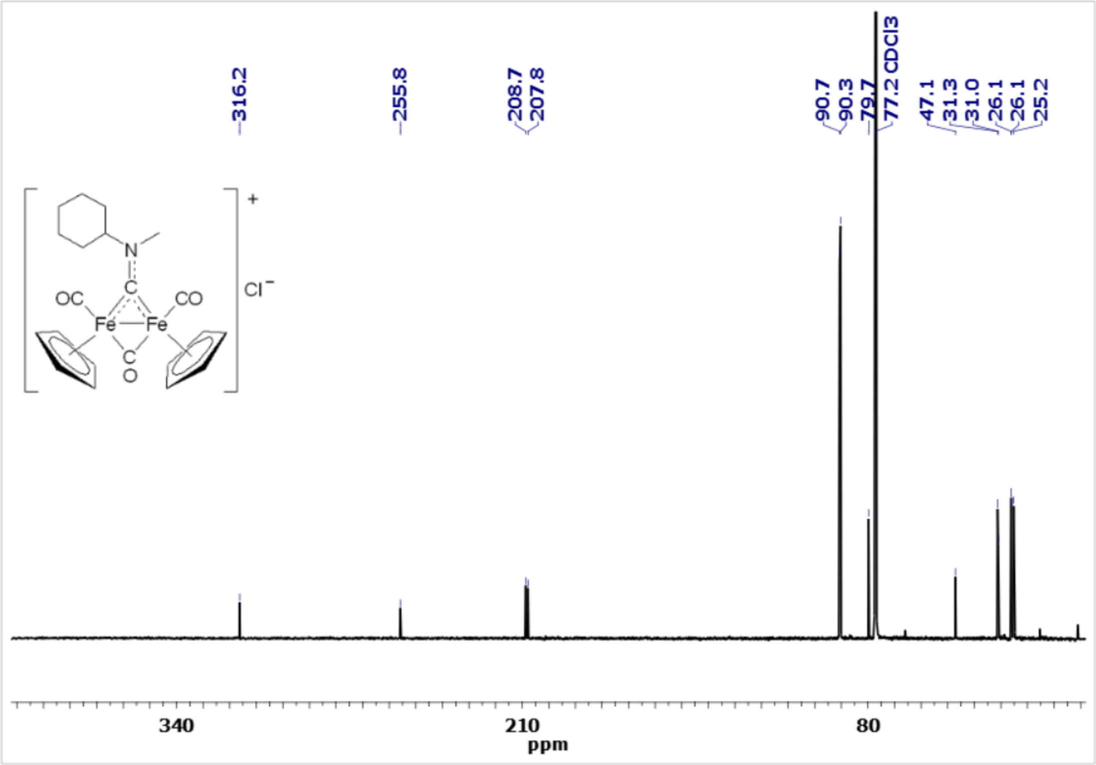

Figure S21. ^{35}Cl NMR spectrum (39 MHz, $CDCl_3$) of [**2C**]Cl.

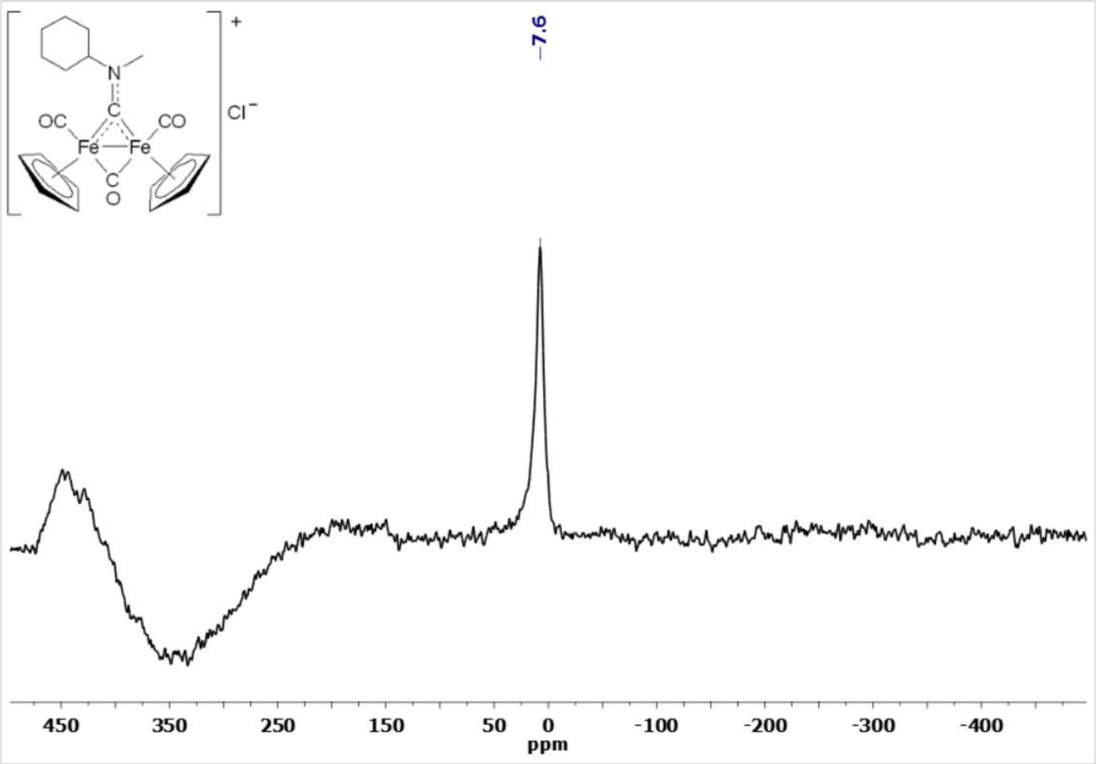

Figure S22. ^{1}H NMR spectrum (401 MHz, $CDCl_3$) of [**2D**]CF_3SO_3. Inset shows the broad aromatic resonance.

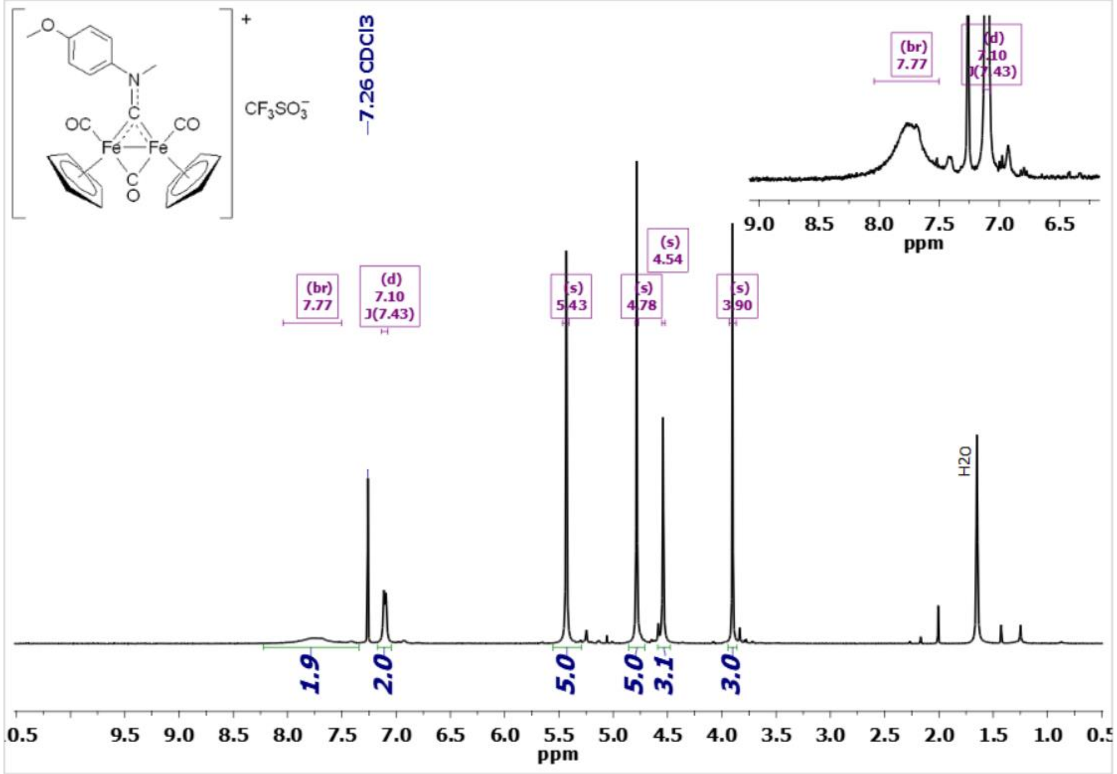

Figure S23. ^{1}H NMR spectrum (401 MHz, $CDCl_3$) of [**3F**]CF_3SO_3.

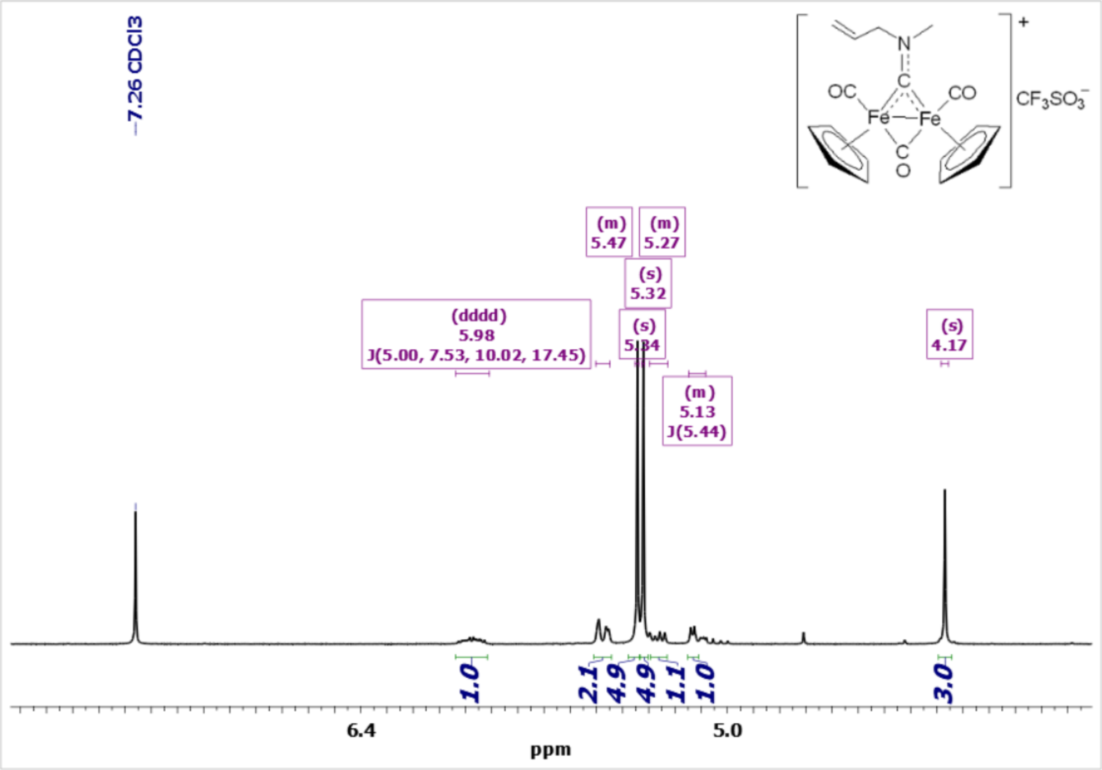

Figure S24. $^{13}C\{^{1}H\}$ NMR spectrum (101 MHz, $CDCl_3$) of [**3F**]CF_3SO_3.

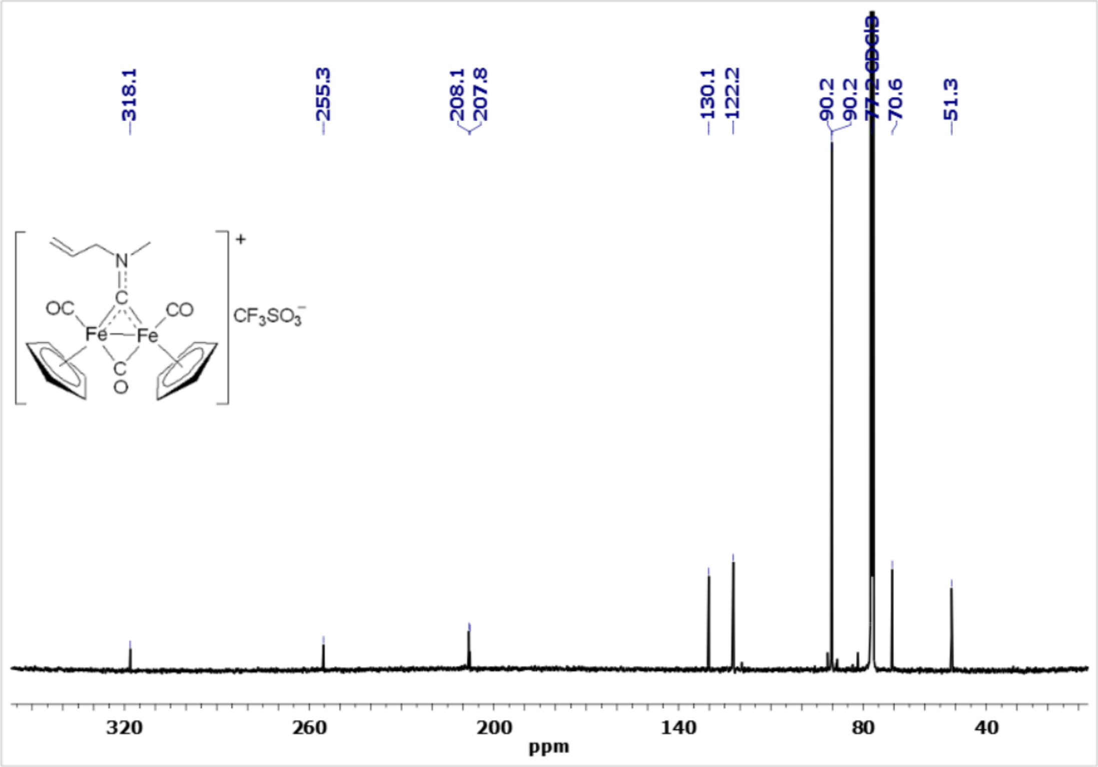

Figure S25. ^{1}H NMR spectrum (401 MHz, acetone-d_6) of [**4G**]BF_4.

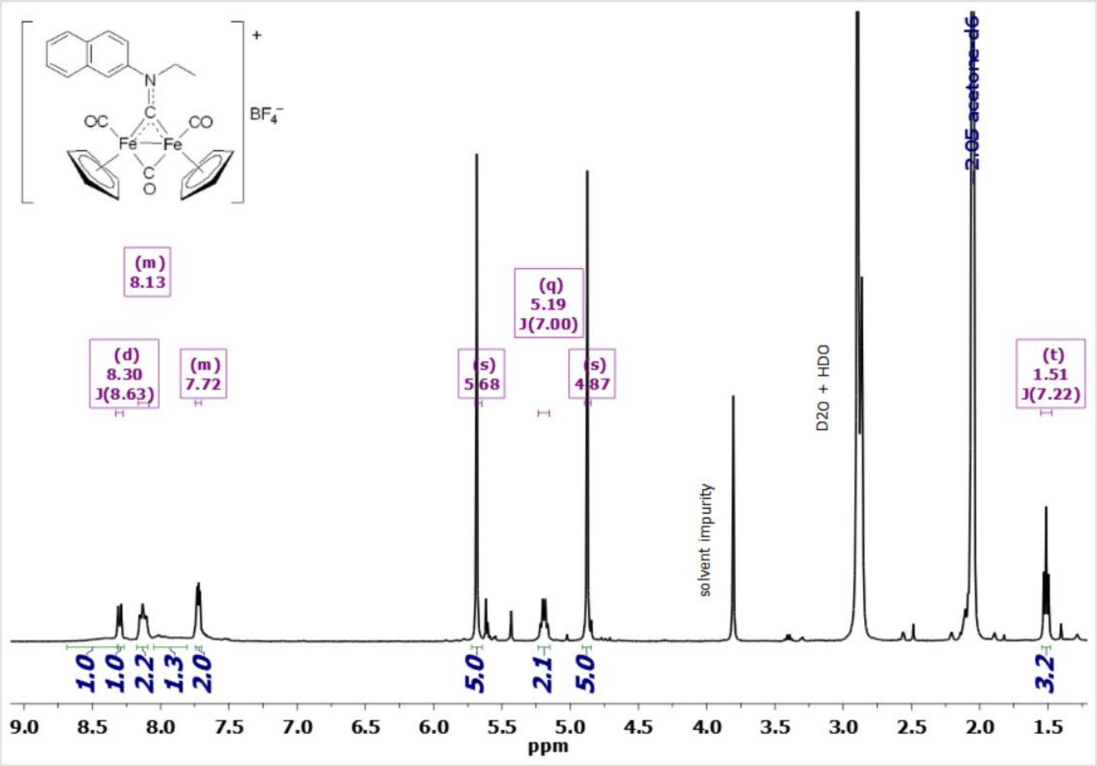

Figure S26. $^{13}C\{^{1}H\}$ NMR spectrum (101 MHz, acetone-d_6) of [**4G**]BF_4.

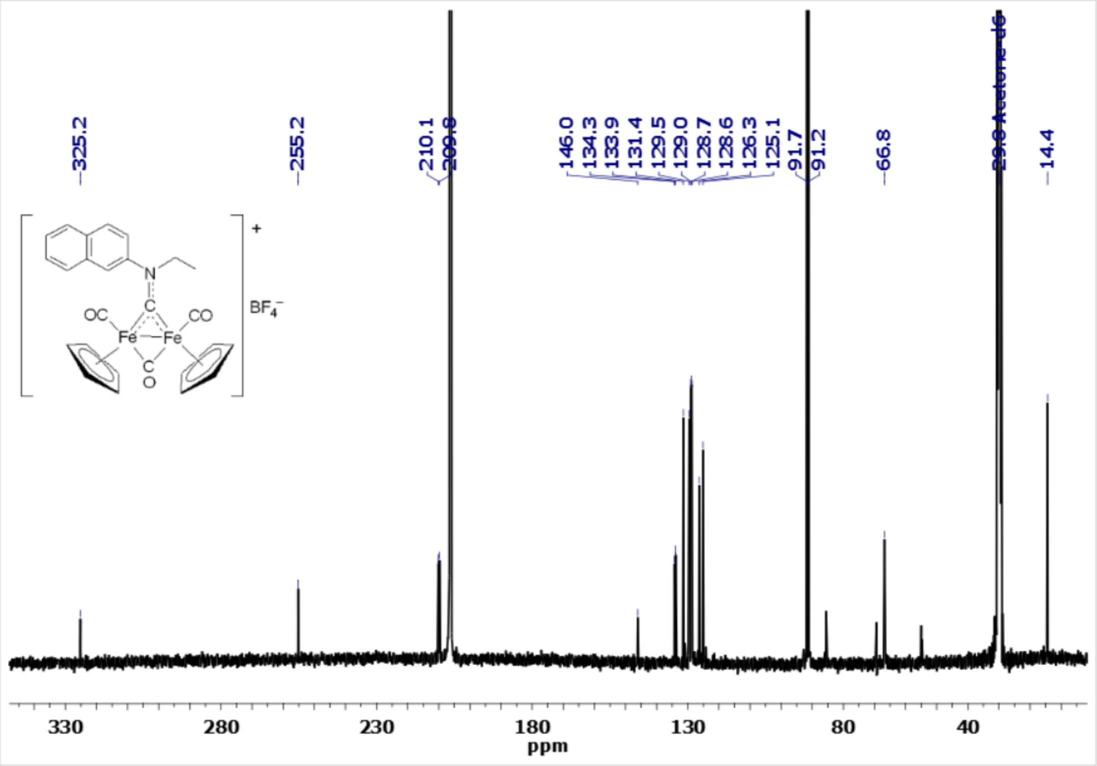

Figure S27. ^{1}H (black line) and ^{1}H-NOE (blue line: irradiation at 4.65 ppm; red line: irradiation at 5.44 ppm) NMR spectra (401 MHz, $CDCl_3$) of [**4G**]BF_4; showing mutual NOE effect for the Cp rings.

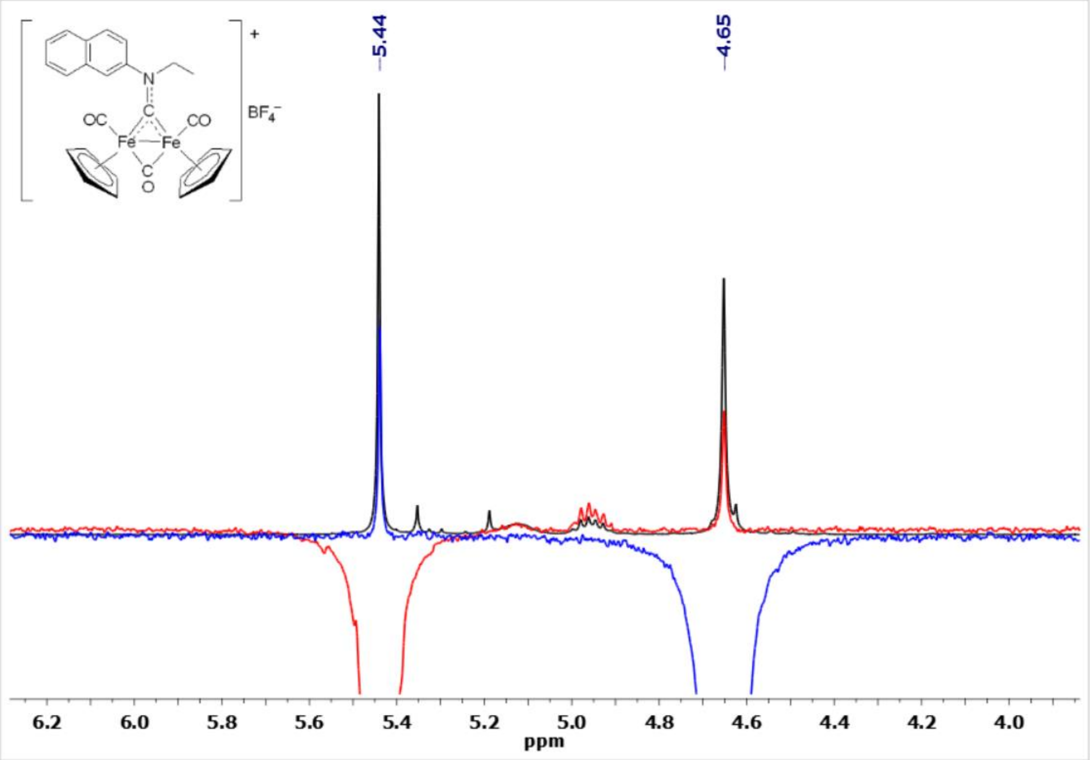

Figure S28. ^{1}H NMR spectrum (401 MHz, $CDCl_3$) of [**5H**]CF_3SO_3.

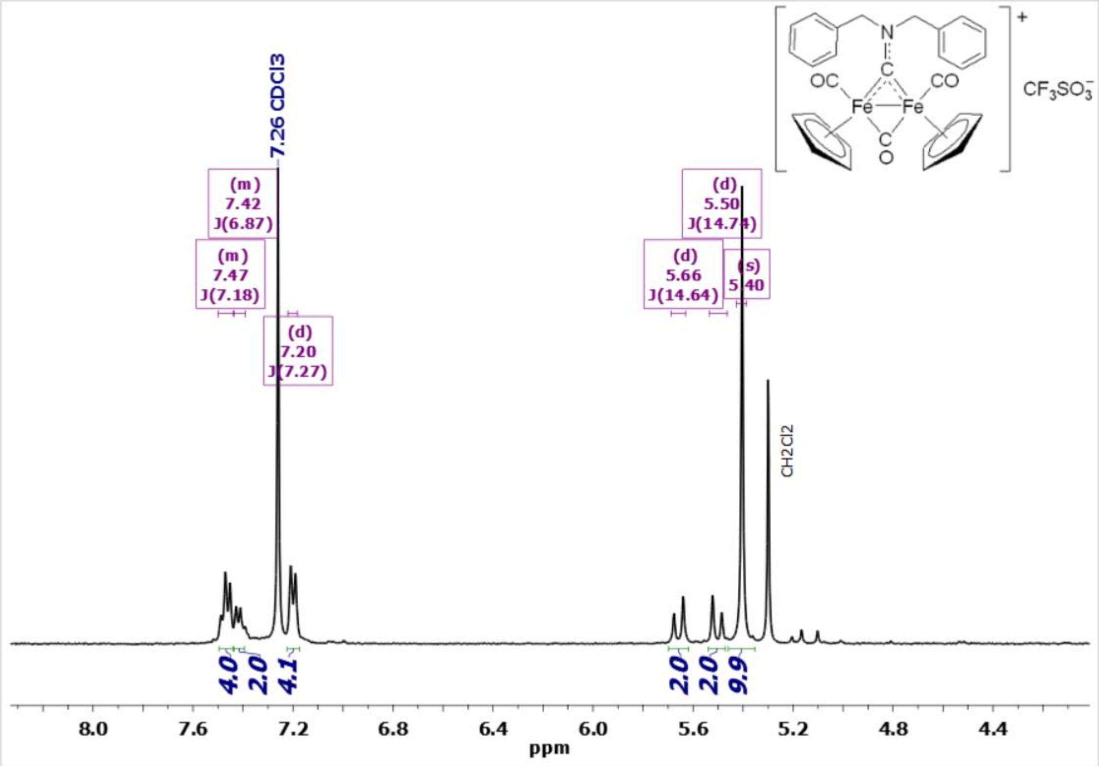

Figure S29. $^{13}C\{^1H\}$ NMR spectrum (101 MHz, $CDCl_3$) of [**5H**]CF_3SO_3.

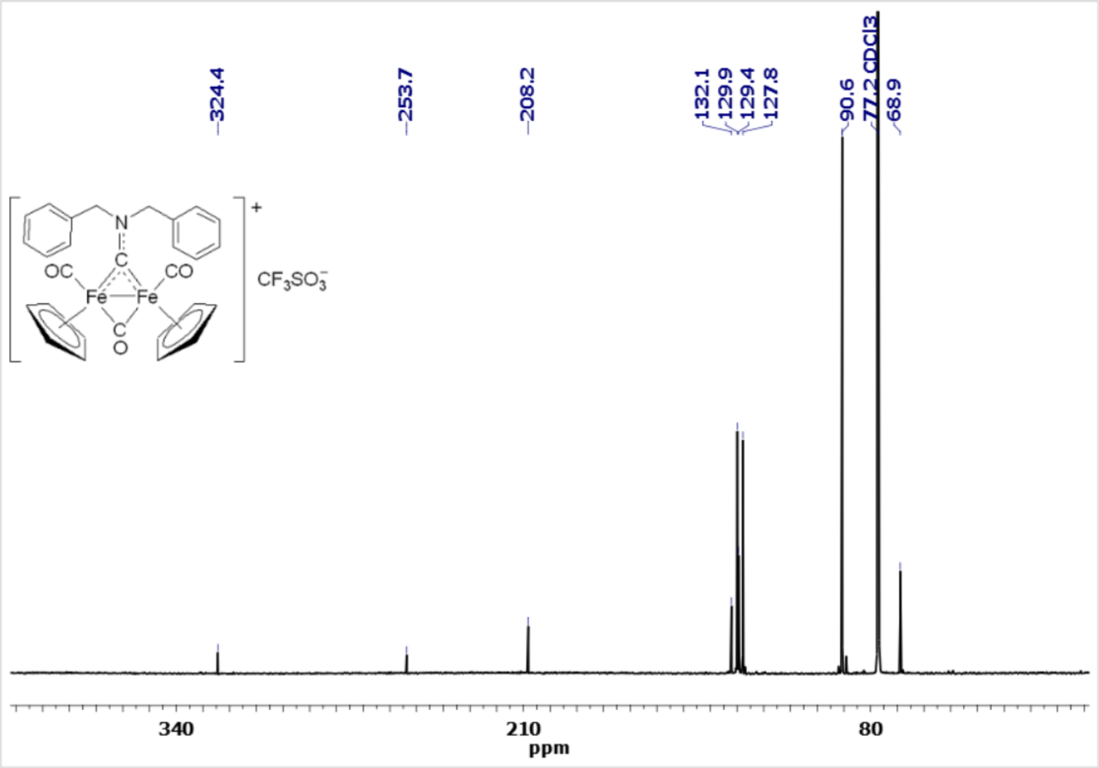

Figure S30. ^1H NMR spectrum (401 MHz, $CDCl_3$) of **6**.

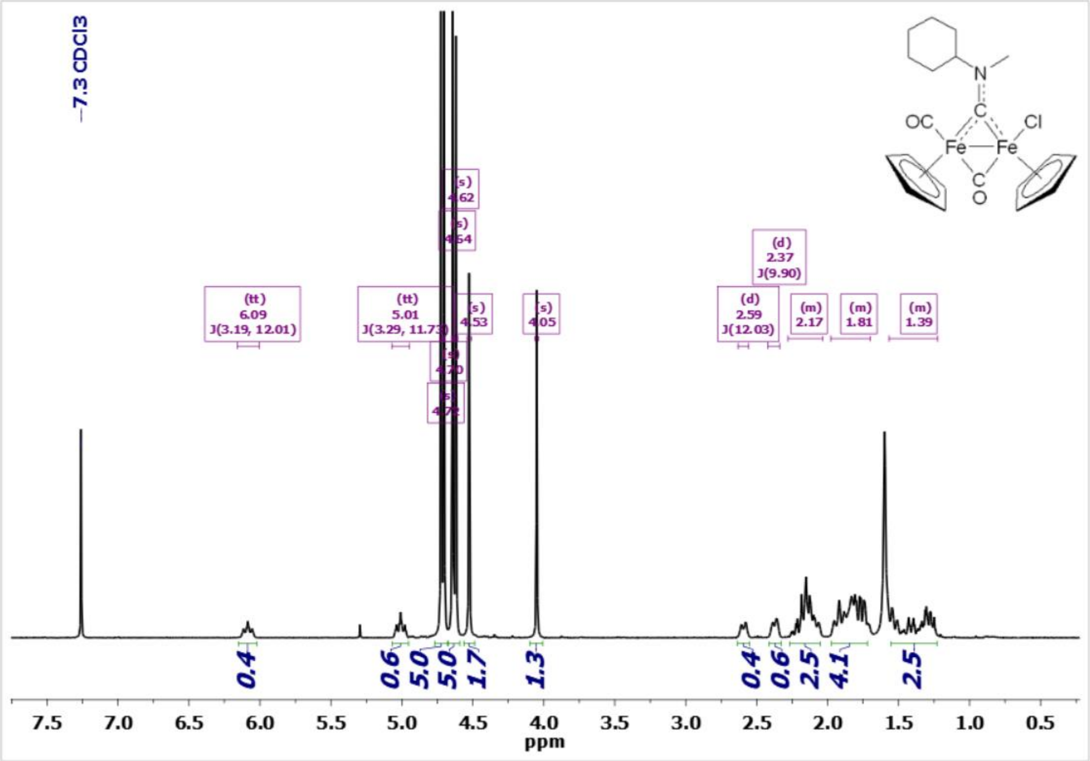

Figure S31. $^{13}C\{^1H\}$ NMR spectrum (101 MHz, $CDCl_3$) of **6**.

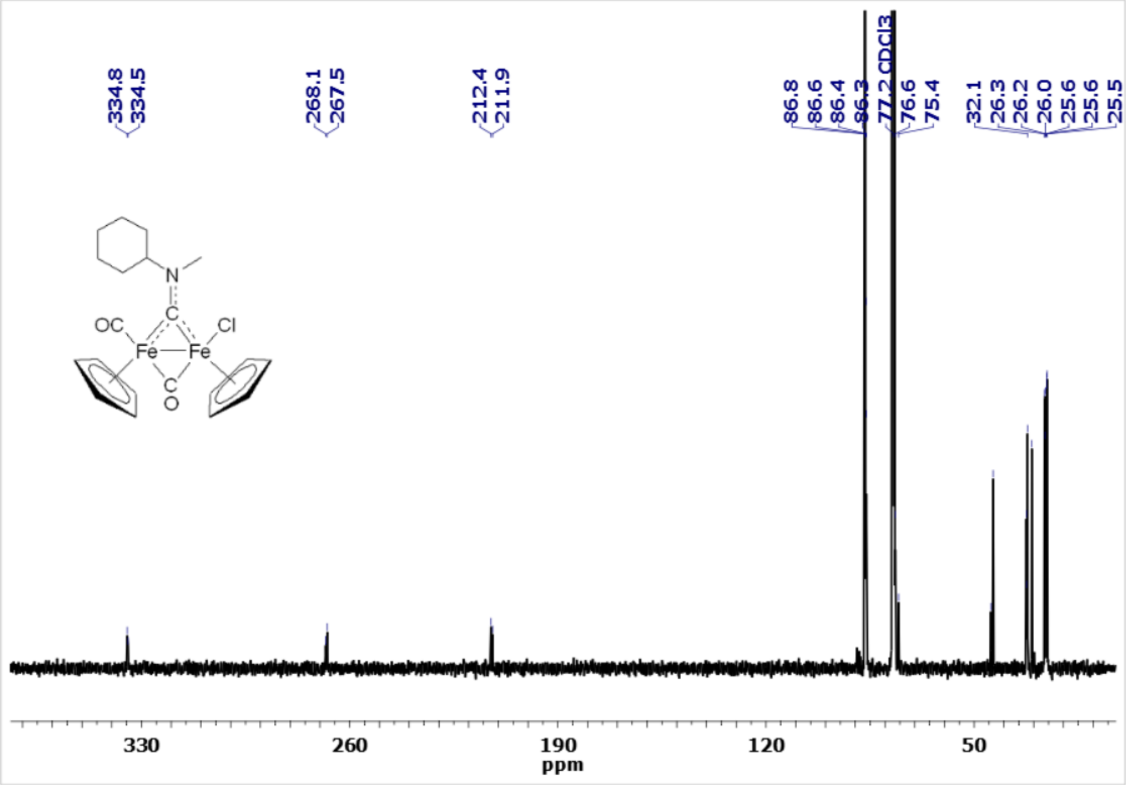

Figure S32. ^1H (black line) and ^1H-NOE (blue line: irradiation at 4.70 ppm; blue line: irradiation at 4.65 ppm) NMR spectra (401 MHz, $CDCl_3$) of **6**, showing mutual NOE effect for the Cp rings (co-irradiation for the two isomers).

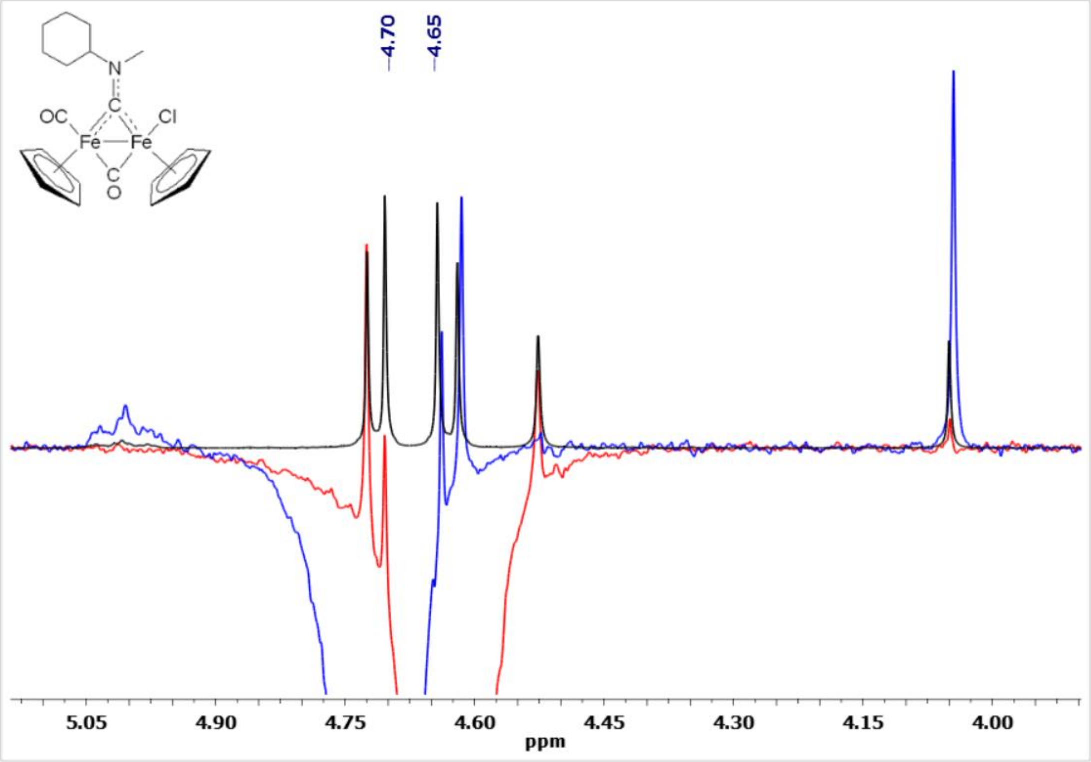

3. DFT-optimized geometries (data).

Compound **1C**

Fe	-2.254121	-2.477913	0.119554
Fe	-1.686951	-1.643968	-2.192534
C	-0.971463	-1.149056	-0.484094
C	-3.235820	-2.543747	-1.514854
O	-4.262692	-2.970861	-1.932563
C	-3.260842	-1.206072	0.723079
O	-3.937405	-0.373301	1.173798
C	-2.562579	-0.151513	-2.235104
O	-3.124614	0.865759	-2.283582
C	-1.785787	-3.256454	2.032191
C	-0.686764	-3.536106	1.182447
C	-1.156749	-4.356347	0.117875
C	-2.545244	-4.611242	0.324225
C	-2.944352	-3.929933	1.497821
C	0.070617	-2.787845	-2.708296
C	-1.078996	-3.416132	-3.276603
C	-1.698128	-2.494477	-4.160239
C	-0.929078	-1.288239	-4.130632
C	0.171769	-1.476240	-3.243512
H	-1.765985	-2.654878	2.930699
H	0.322945	-3.170214	1.299170
H	-0.561383	-4.735210	-0.699238
H	-3.186457	-5.191840	-0.324323
H	0.738618	-3.220470	-1.977194
H	-1.436430	-4.411368	-3.050617
H	-2.603764	-2.658593	-4.727432
H	-1.145901	-0.386891	-4.688457
H	0.916562	-0.737158	-2.984388
H	-3.938433	-3.911257	1.923466
N	-0.129122	-0.359579	-0.052156
C	0.185614	-0.104955	1.356424
C	2.036969	0.005619	3.070900
C	0.101625	1.629764	3.192602
C	1.596045	1.420523	3.464169
C	-0.251294	1.316264	1.734037
C	1.682575	-0.304294	1.611772
H	1.537374	-0.722887	3.726403
H	-0.480945	0.972488	3.854695
H	2.173998	2.152380	2.881080
H	0.254780	2.026093	1.065123
H	2.241516	0.362802	0.940813
H	-0.376910	-0.812742	1.983422
H	3.115833	-0.117250	3.226087
H	-0.191367	2.659412	3.431733
H	1.819425	1.607135	4.521991

H	-1.329079	1.420493	1.563189
H	1.967324	-1.331738	1.353393

Compound [**2A**]$^{+}$

Fe	4.26660131890632	11.82050541092293	3.40771965512903
Fe	6.69486688127077	11.83835114403971	4.04327008242886
N	4.93173473019962	13.43369158078674	5.79813770669982
N	-0.26491961340199	15.22967095642780	6.58514123034587
H	-0.75932428048956	16.08042259003132	6.36250597149380
O	3.12598728623823	9.90564461064224	5.26782130400827
O	6.79534304200172	9.95154230240497	6.24733817843118
O	5.85974045263656	9.59605538186753	2.36016151838261
C	3.59803763490481	10.66672683277300	4.53713295632822
C	6.74035222377735	10.68998168549737	5.35992621000550
C	5.67801112072427	10.58104682817343	2.97965104328506
C	5.20368520054789	12.66495457110873	4.78552315151615
C	5.92793589922694	13.84721961917113	6.80963264955816
H	6.85048433696748	13.29267424716208	6.65650286240734
H	5.51750170605359	13.62866977709548	7.79833104625706
H	6.10956097423129	14.92183880444881	6.72325253991988
C	3.58882505824756	13.95434781261275	6.00757075362095
C	3.30566680465824	15.25557661536361	5.55458499948801
H	4.08809452553212	15.83681851186731	5.07681906707788
C	2.02808090530017	15.78535011968473	5.70301700238824
H	1.79351293864095	16.78470289924739	5.34908425690543
C	1.06010468259119	14.98367045923600	6.31434387569964
C	1.34862726871950	13.66473754075894	6.79548365617206
C	2.64726655463075	13.15533968534956	6.63717406944519
H	2.91084963423507	12.16299015519128	6.98643220490094
C	0.13478939483213	13.15176623425818	7.35980772871904
H	-0.01226659598830	12.18264117378391	7.81598516448043
C	-0.82123365045570	14.13215102001545	7.21212990513645
H	-1.86245011064640	14.14263122946145	7.50313256209453
C	4.31373994555732	12.76676909035276	1.46830598874988
H	5.20376746066459	12.90955706240193	0.87265303090475
C	3.79187742905839	13.67262258249934	2.43785692083617
H	4.22147495404992	14.62394054181794	2.71544811271865
C	2.62220368267430	13.09089761629318	2.99574185034639
H	1.99743686290352	13.51828813771042	3.76552218722092
C	2.42349468520336	11.82044529468723	2.36018395886043
H	1.61923395747341	11.12873713460242	2.57196759552522
C	3.46404054747009	11.62522176646389	1.41535919111958
H	3.60691226755053	10.75215871249872	0.79444864726678
C	7.45061830449803	13.34871682461572	2.68700224144769
H	6.85162482665897	13.99057304004299	2.05889302826015
C	7.98168554758547	12.07123153996466	2.32742477501176
H	7.82885374149848	11.56516459167466	1.38440749615890
C	8.71233250508874	11.57092001432842	3.43001716230629

H	9.22517099470939	10.62070800299406	3.48378461635056
C	8.62996684246606	12.54673959398239	4.48697644794515
H	9.08785444545442	12.45995530390683	5.46283710852819
C	7.86419849915478	13.64521470073461	4.01234046599185
H	7.62296517618766	14.54241564904401	4.56396282212509

Compound [**2C**]$^{+}$

Fe	3.37495317234996	7.78878523058026	10.61664244838150
Fe	4.70039475512489	8.99008985228605	12.37650994566338
C	3.05928787442209	6.23834463451662	11.35441057624161
C	4.78122549031126	7.81769997824191	13.66812449439889
C	2.79557128339912	8.67537190829742	12.22017015910803
O	2.85532947337824	5.19497900345880	11.80810904932277
O	4.86115919654994	7.05265684210402	14.53151413911204
O	1.80547495841830	8.94526802041673	12.79773366591443
N	6.19074653626141	6.91781083900090	10.86437873757142
C	5.16539033979376	7.65767859606137	11.14905309440699
C	6.17121309421278	5.94915507507961	9.74907550980771
H	5.14272589083847	5.67001430339510	9.53687210491978
H	6.73622353105267	5.06226747976998	10.03333503734318
H	6.62421627222428	6.39827304030486	8.86219696805998
C	7.46842712929385	7.01262649371841	11.64187876231725
H	7.26786415364940	7.77478784281196	12.39436498309087
C	8.62937404431573	7.47642285842525	10.76221086349583
H	8.82520965611905	6.72876722446566	9.98475865718217
H	8.36089836729348	8.41224960146357	10.25664381442824
C	9.88800153083326	7.66083857712110	11.62115707556628
H	9.72375982713852	8.48348536174590	12.33160471395043
H	10.72633503135157	7.95720491995009	10.98048917813582
C	10.22808069952727	6.38320984093321	12.39773173721934
H	11.10180924679613	6.55408720146479	13.03744683173026
H	10.50003070195701	5.59174082476877	11.68561100034751
C	9.04012883731751	5.91057108920023	13.24336876640971
H	9.28247992135803	4.97237981731096	13.75558397365606
H	8.82595852450987	6.65428595825401	14.02347151059613
C	7.78687201530778	5.70924380419395	12.37955084270809
H	6.93096496836358	5.41477997998896	12.99677512649149
H	7.97561859896778	4.90067440739859	11.66450784244796
C	2.93778656034339	7.11590689605493	8.66941193719246
H	3.08536204933390	6.09714629147425	8.34018518146542
C	1.73834790508870	7.63744576442796	9.27531564282809
H	0.84293098340447	7.07236981470380	9.49485782556119
C	1.95170136835746	9.00886140024100	9.54256499636553
H	1.24978568562401	9.68084189217880	10.01583225497643
C	3.27071452922187	9.34461408045331	9.11051674051070
H	3.72531442617270	10.32111443533143	9.17489303765337
C	3.87171220578319	8.18010166659045	8.56069273802603
H	4.86879613533799	8.11621400736452	8.14924074685369

C	4.44851007223575	10.89699550607756	13.32419773790590
H	3.68108085942111	11.09090221481155	14.05978084875680
C	5.75292352436033	10.38024941541469	13.58469771557458
H	6.15555492245780	10.13549355687424	14.55815982869323
C	6.44032215585299	10.24941774593649	12.34046887821858
H	7.45855555025320	9.91448412706208	12.21134153605352
C	5.55646851337026	10.65958340030152	11.31017031502549
H	5.77953476750458	10.66242680624014	10.25278415638787
C	4.32836502924565	11.06078293280615	11.91882569974709
H	3.44721463419364	11.41247843892600	11.40063057217889

Compound **6** (E isomer)

Cl	1.52770965126137	13.50768165199517	5.78377890545806
Fe	3.54965964496935	14.41904679385791	6.56732177714066
Fe	4.06035650642669	16.09164380648336	4.79685742187595
N	4.55524800888543	13.27375315350440	4.05139080540568
O	1.84320309348211	15.93288004547722	2.94519090165462
O	2.09506706636255	16.91097090084683	6.81756252766878
C	2.70734332745656	15.95955784640397	3.72231634079512
C	2.82769850112100	16.10725222483469	6.34246588274700
C	4.21481927373499	14.22429689078015	4.87661325240674
C	4.49291978501603	11.85402867682388	4.44725607318915
H	4.27557981140354	11.24437956508770	3.56975511680655
H	5.44873723814672	11.53660619261461	4.87374713834345
H	3.69444462822050	11.73336747839224	5.17733033338965
C	4.98219430101693	13.56071698127368	2.65362533991403
H	5.02117632268412	14.64888111319130	2.59840609954042
C	6.37795251347464	13.00994505545838	2.35238687375570
H	7.09070650181930	13.37382015247722	3.10246547588301
H	6.36864260263200	11.91437230983605	2.41854374126991
C	6.80975640239331	13.43199434047023	0.94119174617928
H	7.79677971982949	13.01128469663799	0.71581939415121
H	6.91534826195364	14.52594027107586	0.91043069119268
C	5.78659114462760	12.99200005571735	-0.11263530231679
H	5.76319660287629	11.89363935020751	-0.15458199934222
H	6.09373129680854	13.34380992144480	-1.10483480998338
C	4.38238510043677	13.51133338415776	0.21985606344089
H	4.37700944642668	14.60989103004225	0.15823168411528
H	3.65471352968854	13.14930447036419	-0.51575473611076
C	3.94803842832290	13.08378246483664	1.62876847542032
H	3.85887843052390	11.99101610992778	1.65932964618602
H	2.96747584332000	13.50098427087660	1.87941653866315
C	5.34491674257676	14.02276108589139	7.57346594901914
H	6.31708914730928	14.10625724332639	7.10807791854846
C	4.54617539419091	12.84492181042301	7.64132706851189
H	4.79741906115000	11.87518417580747	7.24056202187023
C	3.34434483995877	13.17972706180511	8.32720590118963
H	2.50164621600695	12.51768306165864	8.47095240100548

C	3.40136037087420	14.53870398623377	8.72306517972905
H	2.62590689160611	15.08863923008873	9.23768951421099
C	4.63434216507804	15.07118893448989	8.24186643850893
H	4.97144638194392	16.09058525935928	8.36726522786352
C	6.12098705457607	16.43913376741097	4.25657690418599
H	6.80511743986082	15.66619352650829	3.93637448819915
C	5.24089993673031	17.18148335389727	3.42596490689090
H	5.14295648388180	17.09361588716377	2.35211081056433
C	4.50944528780361	18.09932349652207	4.26209530578490
H	3.75918771822312	18.80157391622613	3.92514374761176
C	4.93665107656201	17.90189999308764	5.59539420261237
H	4.55798289710378	18.41692162017010	6.46688638560099
C	5.92541737666294	16.87145569043253	5.59611303894098
H	6.43914353257912	16.48730269439973	6.46434319031114

Compound **6** (Z isomer)

Cl	1.58246675232065	13.45666515288726	5.80892370036800
Fe	3.59481993805352	14.40454857366358	6.57600486268605
Fe	4.08516159698514	16.08758146665192	4.80983636160339
N	4.57786797378862	13.28114665359618	4.03592418752511
O	1.89707507770019	15.85171827864091	2.93191588371996
O	2.09000643175344	16.86803126001982	6.81449941606916
C	2.74922789657583	15.91771449093597	3.71967499831235
C	2.84198005796165	16.08084859115108	6.34192390529506
C	4.25537965511809	14.22432499747925	4.87661394304902
C	4.98601484938099	13.55956310070336	2.64901261366176
H	5.85968484671835	12.95547427595773	2.39235164354105
H	4.16805630779540	13.31951296156938	1.96435693143001
H	5.23194348277674	14.61266258869622	2.55530999987362
C	4.45449157228294	11.84211643994747	4.41334340852963
H	3.90078098137387	11.85893521914055	5.35195000554674
C	3.62485945648550	11.04504493450883	3.40461011386368
H	2.65235572387930	11.53102425623089	3.27043870166134
H	4.12878124424538	11.02036897339133	2.43103865584591
C	3.44926084056532	9.60711265227054	3.91249355702739
H	2.86615120906612	9.02958891053598	3.18545904627300
H	2.86969053019350	9.62438957090867	4.84627154907546
C	4.80443453026333	8.93574648318457	4.16515681377461
H	5.33857828264159	8.83418650726695	3.20950005850069
H	4.65863081456610	7.92239133793158	4.55859083488366
C	5.66107189505010	9.75895709489836	5.13404880863683
H	5.17959154911135	9.76334527806906	6.12270113516410
H	6.64702112146040	9.29810441881025	5.26546358604197
C	5.82618155305387	11.20518151335302	4.64593546933371
H	6.40005472495225	11.20687965803759	3.71036864054997
H	6.38905043648257	11.80041848097273	5.37333112739830
C	5.39568337136720	14.05772761651653	7.59258591800547
H	6.36784927846022	14.16222069355990	7.13202004214170

C	4.62726857716430	12.86092873525086	7.66708085226911
H	4.90852441464260	11.89221297503980	7.28482345130010
C	3.41426332528201	13.16912948825817	8.34393011566139
H	2.58881965957113	12.48588591743288	8.48870857554709
C	3.43459988868032	14.53221379028050	8.73056548394823
H	2.64302622862311	15.06591119103663	9.23759749058109
C	4.65601982791653	15.09200003446258	8.25164377214650
H	4.96683869305221	16.12023772060046	8.37169766822498
C	6.14637436511493	16.46170669033470	4.29467402748594
H	6.84365291659390	15.69268237492084	3.99158470876674
C	5.26571814514988	17.18204657386657	3.44464530954153
H	5.18233237987057	17.08015066804972	2.37086124455475
C	4.50901407351212	18.09671775104502	4.26162587514516
H	3.75185557029069	18.78318193019487	3.90791775216217
C	4.92170741871584	17.92037413740827	5.60241208547836
H	4.52368659401473	18.43834491694813	6.46348618593816
C	5.92712737827115	16.90638249364078	5.62661811112278
H	6.43596656110441	16.54036017974169	6.50547137073724

4. Stability studies in aqueous media.

Stability in water.

A mixture of the selected Fe compound (*ca*. 4 mg) and a D_2O solution (0.9 mL) containing Me_2SO_2 ($3.36 \cdot 10^{-3}$ M) was stirred for 30 minutes then filtered over celite and transferred into an NMR tube. The orange-red solution was analyzed by ^1H, $^{19}F\{^1H\}$, $^{31}P\{^1H\}$, ^{35}Cl NMR then heated at 37 °C for 72 h. After cooling to room temperature, the final solution was separated from an orange-brown solid by filtration over celite, and the ^1H NMR spectrum was recorded (delay time = 3 s; number of scans = 20). In each case, no new {FeCp} species was identified. The amount of starting material in solution (% with respect to the initial spectrum) was calculated by the relative integral with respect to Me_2SO_2 as internal standard [3] (δ/ppm = 3.14 (s, 6H)) (Table S2). The aqueous solution was then extracted with $CDCl_3$ (3 x 0.25 mL) and analyzed by ^1H and ^{19}F NMR, in order to confirm the identity of the compounds.

[2A]CF_3SO_3. ^1H NMR (D_2O): δ/ppm = 8.47, 7.96, 7.79, 7.60, 6.76, 5.47, 4.62. ^{19}F NMR (D_2O): δ/ppm = – 78.8.

[2B]BF_4. ^1H NMR (D_2O): δ/ppm = 5.41, 5.39 (s, 10H); 5.27–5.21 (dd, $^2J_{HP}$ = 12.7 Hz, $^2J_{HH}$ = 15.5 Hz, 1H), 5.13–5.03 (dd, $^2J_{HP}$ = 13.1 Hz, $^2J_{HH}$ = 15.7 Hz,, 1H), 4.38 (app. quint, $^3J_{HH}$ = $^3J_{HP}$ = 7.5 Hz, 4H), 4.30 (s, 3H), 1.43 (t, *J* = 7.0 Hz, 6H). ^{19}F NMR (D_2O): δ/ppm = –150.4, –150.5. ^{31}P NMR (D_2O): δ/ppm = 19.6.

[2C]CF_3SO_3. ^1H NMR (D_2O): δ/ppm = 5.37, 5.35 (s, 10H); 4.08 (s, 3H), 2.36 (d, *J* = 10.7 Hz, 1H); 2.19–2.03 (m, 2H), 1.99–1.74 (m, 4H), 1.69–1.45 (m, 3H), 1.38–1.27 (m, 1H). ^{19}F NMR (D_2O): δ/ppm = –78.8.

[2C]Cl. ^1H NMR (D_2O): δ/ppm = 5.37, 5.35 (s, 10 H); 4.07 (s, 3H), 2.35 (d, *J* = 12.1 Hz, 1H); 2.19–2.04 (m 2H), 1.98–1.75 (m, 4H), 1.70–1.46 (m, 2H), 1.38–1.27 (m, 2H). ^{35}Cl NMR (D_2O): δ/ppm = 0.35 ($\Delta\nu_{1/2}$ = 17 Hz).

[2D]CF_3SO_3. ^1H NMR (D_2O): δ/ppm = 7.65 (d, *J* = 8.2 Hz, 2H), 7.27 (d, *J* = 8.3 Hz, 2H); 5.45, 4.83 (s, 10H); 4.54 (s, 3H), 3.96 (s, 3H). ^{19}F NMR (D_2O): δ/ppm = – 78.9.

[3F]I. ^1H NMR (D_2O): δ/ppm = 6.28–6.17 (m, 1H), 5.58 (app. d, *J* = 12.4 Hz, 2H); 5.39, 5.36 (s, 10H); 5.19 (dd, *J* = 15.5, 4.8 Hz, 1H), 5.07 (dd, *J* = 15.1, 5.1 Hz, 1H), 4.16 (s, 3H).

[5H]CF_3SO_3. ^1H NMR (D_2O): δ/ppm = 7.57–7.47 (m, 6H), 7.35–7.29 (m, 4H), 5.77 (d, *J* = 15.8 Hz, 2H), 5.65 (d, *J* = 14.8 Hz, 2H), 5.43 (s, 10H). ^{19}F NMR (D_2O): δ/ppm = – 78.8.

Isolation and identification of the precipitate from H_2O.

A suspension of [**2C**]CF_3SO_3 (57 mg, 0.095 mmol) in H_2O (20 mL) was vigorously stirred at room temperature for 30 minutes then filtered over celite. The resulting red solution was stirred at 37 °C for 72 h, then cooled to room temperature and filtered (G4 porous filter). The resulting brown solid was washed with water and dried under vacuum (50 °C). Yield: 6 mg. The sample was analyzed by Raman spectroscopy and identified as γ-Fe_2O_3 (maghemite, Raman shift: 711 cm^{-1}).

Stability in water/methanol.

A solution of the selected Fe compound in a D_2O/CD_3OD mixture (2:1 *v/v* for [**2A**]CF_3SO_3, [**2B**]BF_4; 5:4 *v/v* for [**5H**]CF_3SO_3, [**4G**]BF_4) was prepared and treated as described above. The residual amount of starting material in solution after 72 h at 37 °C was calculated with respect to Me_2SO_2 as internal standard (Table S2).

[2A]CF_3SO_3. ^1H NMR (D_2O/CD_3OD 2:1): δ/ppm = 7.89 (s, 1H), 7.76 (d, *J* = 8.4 Hz, 1H), 7.58 (d, *J* = 3.1 Hz, 1H), 7.48–7.39 (m, 1H), 6.73 (d, *J* = 2.8 Hz, 1H); 5.48, 4.73 (s, 10H), 4.63 (s, 3H).

[2B]BF_4. ^1H NMR: δ/ppm (D_2O/CD_3OD 2:1): δ/ppm = 5.41, 5.40 (s, 10H); 5.27–5.18 (m, 1H), 5.12–5.03 (m, 1H), 4.37 (dt, *J* = 13.9, 6.9 Hz, 1H), 4.30 (s, 3H), 1.43 (td, *J* = 7.0, 3.2 Hz, 6H). $^{31}P\{^1H\}$ NMR: δ/ppm (D_2O/CD_3OD 2:1): δ/ppm = 20.5.

[4G]BF$_4$. ^{1}H NMR (D_2O/CD_3OD 5:4): δ/ppm = 8.29 (d, *J* = 8.6 Hz, 1H), 8.19–7.98 (m, 3H), 7.80–7.73 (m, 3H), 5.52 (s, 5H), 5.10–5.01* (m), 4.72 (s, 5H), 1.51 (t, *J* = 7.2 Hz, 3H). *Over OH residual peak.

[5H]CF$_3$SO$_3$. ^{1}H NMR (D_2O/CD_3OD 5:4): δ/ppm = 7.56–7.49 (m, 6H), 7.29 (d, *J* = 7.2 Hz, 4H), 5.76 (d, *J* = 15.3 Hz, 2H), 5.62 (d, *J* = 15.3 Hz, 2H), 5.44 (s, 10H).

Stability in DMSO/water.

A solution of the selected Fe compound in a DMSO-d$_6$/D_2O mixture (1:1 *v/v* for [**2A**]CF_3SO_3, [**5H**]CF_3CO_3; 5:3 *v/v* for [**4G**]BF_4) was prepared and treated as described above. In this case, formation of an orange-brown precipitate was not observed. However, after 72 h at 37 °C, a new set of signals in the ^{1}H spectrum was detected, attributed to the DMSO adduct $[Fe_2Cp_2(CO)(\mu\text{-}CO)\{\mu\text{-}CNMeR\}(DMSO)]^+$ ([**2A^S**]$^+$, [**4G^S**]$^+$, [**5H^S**]$^+$; Scheme S2).[1] The residual amount of starting material in solution was calculated with respect to Me_2SO_2 as internal standard (Table S2).

[2A]CF$_3$SO$_3$. ^{1}H NMR (DMSO-d$_6$/D_2O 1:1): δ/ppm = 7.78 (s, 1H), 7.62 (d, *J* = 8.4 Hz, 1H), 7.45 (s, 1H), 7.28 (s, 1H), 6.60 (s, 1H); 5.37, 4.61 (s, 10H). ^{19}F NMR (DMSO-d$_6$/D_2O 1:1): δ/ppm = – 77.8.

[2A^S]$^+$. ^{1}H NMR (DMSO-d$_6$/D_2O 1:1): δ/ppm = 6.56 (s); 5.25, 5.06 (s, 5H); 4.70 (s, 3H); 4.50 (s).

[4G]BF$_4$. ^{1}H (DMSO-d$_6$/D_2O 5:3): δ/ppm = 8.14 (d, *J* = 8.7 Hz, 1H), 8.10–7.85 (m, 3H), 7.64–7.60 (m, 3H), 5.36 (s, 5H), 4.93–4.80 (m, 2H), 4.57* (s), 1.32 (t, *J* = 7.2 Hz, 3H). ^{19}F NMR (DMSO-d$_6$/D_2O 5:3): δ/ppm = – 148.69, – 148.74.

[4G^S]$^+$. ^{1}H NMR (DMSO-d$_6$/D_2O 5:3): δ/ppm = 5.25, 5.06 (s, 5H); 4.47* (s), 1.35 (m, 3H). *Superimposed on HDO peak.

[5H]CF$_3$SO$_3$. ^{1}H (DMSO-d$_6$/D_2O 1:1): δ/ppm = 7.44–7.34 (m, 6H), 7.16 (d, *J* = 6.7 Hz, 4H), 5.61 (d, *J* = 15.5 Hz, 2H), 5.47 (d, *J* = 15.4 Hz, 2H), 5.30 (s, 10H). ^{19}F NMR (DMSO-d$_6$/D_2O 1:1): δ/ppm = – 78.1.

[5H^S]$^+$. ^{1}H NMR (DMSO-d$_6$/D_2O 1:1): δ/ppm = 6.22 (d, *J* = 15.7 Hz, 1H), 5.70 (d, *J* = 15.1 Hz, 1H); 5.26, 4.97 (s, 10H).

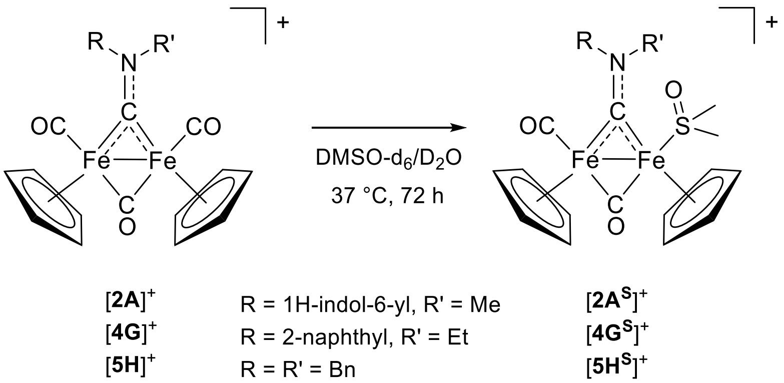

Scheme S2. Formation of aminocarbyne-DMSO complexes in DMSO/water solutions of [**2A**]$^+$, [**4G**]$^+$ and [**5H**]$^+$ at 37 °C.

Stability in aqueous solution (isocyanide complexes).

A solution of the selected Fe compound in an organic solvent/D_2O 2:1 *v*/*v* mixture (CD_3OD for **1B**; acetone-d_6 for **1C**) was prepared as described above and maintained at 37 °C for 24 h. After cooling to room temperature, the final solution was separated from an orange-brown solid by filtration over celite and $^1H/^{31}P$ NMR analyses were repeated. Next, volatiles were removed under vacuum and the residue was analyzed by IR (CH_2Cl_2). In both cases, the starting material was identified as the major species in the final solution, along with other products.

1B. Red-violet solution. ^1H NMR (CD_3OD/D_2O 2:1): δ/ppm = 5.0 (br, 10H), 4.5 (br, 2H), 4.3 (br, 4H), 1.4 (br, 6H). Signal for CH hidden by HDO peak. $^{31}P\{^1H\}$ NMR (CD_3OD/D_2O 2:1): δ/ppm = 24.6.

1C. Red-brown solution. ^1H NMR (acetone-d_6/D_2O 2:1): δ/ppm = 4.7, 4.6 (br, 10H); 3.4 (br, 1H), 1.5 (br, 4H), 1.2 (br, 6H). *Other species* (24 h). ^1H NMR (acetone-d_6;D_2O 2:1): δ/ppm = 6.5, 6.4, 2.6 (br, CpH); 5.3, 4.9, 2.8, 1.8, 1.7 (br). IR (CH_2Cl_2): $\tilde{\nu}$/cm^{-1} = 2201m, 2179m, 2025m, 1995m, 1771m-sh.

Stability in cell culture medium.

Method A. Powdered DMEM cell culture medium (1000 mg/L glucose and L-glutamine, without sodium bicarbonate and phenol red; D2902 - Sigma Aldrich) was dissolved in D_2O (10 mg/mL), according to the manufacturer's instructions. The solution of deuterated cell culture medium ("DMEM-d") was treated with Me_2SO_2 ($6.6 \cdot 10^{-3}$ M) and NaH_2PO_4 / Na_2HPO_4 (0.15 M, pD = 7.5 [4]), then stored at 4 °C under N_2. The selected Fe compound (2-3 mg) was dissolved in CD_3OD (0.05 mL; 0.2 mL for [**2A**]CF_3SO_3; 0.3 mL for [**4G**]BF_4 and [**5H**]CF_3SO_3) then diluted with DMEM-d up to 0.75 mL total volume (c_{Fe2} *ca*. $6 \cdot 10^{-3}$ M; *ca*. 6.6% MeOH). The mixture was stirred for 30 minutes then filtered over celite and transferred into an NMR tube. The resulting red-orange solution was analyzed by ^1H and ^{31}P NMR (delay time = 3 s; number of scans = 20) then heated at 37 °C for 24 h. After cooling to room temperature, the solution was filtered over celite and NMR analyses were repeated. The amount of starting material (% with respect to the initial spectrum) was calculated by the relative integral with respect to Me_2SO_2 as internal standard[3] (δ/ppm = 2.95 (s, 6H)) (Table S2).

Method B. The selected Fe compound (*ca*. 6 mg) was dissolved in DMSO (0.1 mL; 0.5 mL for [**2A**]CF_3SO_3, [**4G**]BF_4 and [**5H**]CF_3SO_3) then diluted with RPMI-1640 cell culture medium (5.0 mL; Merck; modified with sodium bicarbonate, without L-glutamine and phenol red). The orange-red solution was heated at 37 °C for 72 h then allowed to cool to room temperature. The resulting suspension was diluted with water (5 mL) and extracted with CH_2Cl_2 (3 x 10 mL). The combined organic extracts were dried under vacuum (40 °C) and the residue was analyzed by IR (CH_2Cl_2), ^1H and $^{31}P\{^1H\}$ NMR ($CDCl_3$; CD_3CN for [**2A**]CF_3SO_3). In all cases, the starting material was identified as the major species. Its relative amount (%) with respect to other {FeCp} compounds, calculated by ^1H NMR on the basis of Cp and NMe resonances (δ_H: 6.0–3.5 ppm), resulted ≥ 90 % for all the compounds except [**4G**]BF_4 and [**5H**]CF_3SO_3 (*ca*. 75%).

Table S3. Stability of diiron complexes in D_2O, D_2O/CD_3OD, DMSO-d_6/D_2O and cell culture medium (DMEM-d/CD_3OD) solution at 37 °C calculated by ^{1}H NMR (Me_2SO_2 internal standard).

Compound	% starting material (72 h)			% starting material (24 h)
	D_2O	D_2O/CD_3OD	DMSO-d_6/D_2O	DMEM-d (6.6 % CD_3OD)
[**2A**]CF_3SO_3		55 (2:1 *v*/*v*)	89 (1:1 *v*/*v*)	86
[**2B**]BF_4	85	81 (2:1 *v*/*v*)		88
[**2C**]CF_3SO_3	75			85
[**2C**]Cl	79			88
[**2D**]CF_3SO_3	86			77
[**2E**]CF_3SO_3 [b]	51	80 (5:2 *v*/*v*)		70
[**2F**]CF_3SO_3 [b]	70	83 (5:2 *v*/*v*)		76
[**3F**]X [a]	60			84
[**4G**]BF_4		51 (5:4 *v*/*v*)	42 (5:3 *v*/*v*)	85
[**5H**]CF_3SO_3	*ca.* 50	65 (5:4 *v*/*v*)	89 (1:1 *v*/*v*)	75

[a] X = I for the stability in D_2O, X = CF_3SO_3 for the stability in cell culture medium. [b] Stability in D_2O and D_2O/CD_3OD taken from the literature.[1]

Table S4. Release of CO (number of equivalents) over a 24 h period as determined by GC-TCD analysis and stability of selected Fe compounds after 48 h in a 5 % MeOH aqueous solution at 37 °C.

Compound	eq. CO released [a]		% residual complex[b]
	0-24 h	24-48 h	48 h
[**2C**]CF_3SO_3	0.25 ± 0.02	0.23 ± 0.04	83-86
[**2E**]CF_3SO_3	0.31 ± 0.07	0.29 ± 0.04	72-78
[**2F**]CF_3SO_3	0.32 ± 0.03	0.28 ± 0.06	78-82
[**3F**]CF_3SO_3	0.22 ± 0.06	0.15 ± 0.01	87-88
[**5H**]CF_3SO_3	0.29 ± 0.06	0.27 ± 0.02	80-84

[a] $eq_{CO} = n_{CO}/n_{Fe}$. [b] Calculated as $[1 - \Sigma(eq_{CO})/3]\cdot 100$.

Table S5. In vitro antitumor activity of diiron complexes in PSN-1 cancer cells after 24 hours. Values after 72 h are also provided for sake of comparison.

**Compound**	**IC_{50} (μM) ± SD 24 h**	**IC_{50} (μM) ± SD 72 h**
[**2A**]CF_3SO_3	144 ± 16	54.3 ± 6.2
[**2B**]BF_4	> 200	> 100
[**2C**]CF_3SO_3	94.2 ± 3.8	15.3 ± 2.7
[**2D**]CF_3SO_3	35.0 ± 2.0	16.2 ± 2.9
[**2E**]CF_3SO_3	156.0 ± 4.0	41.7 ± 6.3
[**3F**]CF_3SO_3	> 200	> 100
[**3G**]CF_3SO_3	136.4 ± 3.2	26.3 ± 2.8
[**4G**]BF_4	69.5 ± 2.1	25.6 ± 4.5
[**5H**]CF_3SO_3	112.4 ± 2.4	33.7 ± 4.2

Cells (3 x 10^3 mL^{-1}) were treated for 24 h with increasing concentrations of the tested compounds. The cytotoxicity was assessed by the MTT test. IC_{50} values were calculated by a four-parameter logistic model 4-PL ($P < 0.05$).

6. Lysozyme interaction studies (MS spectra)

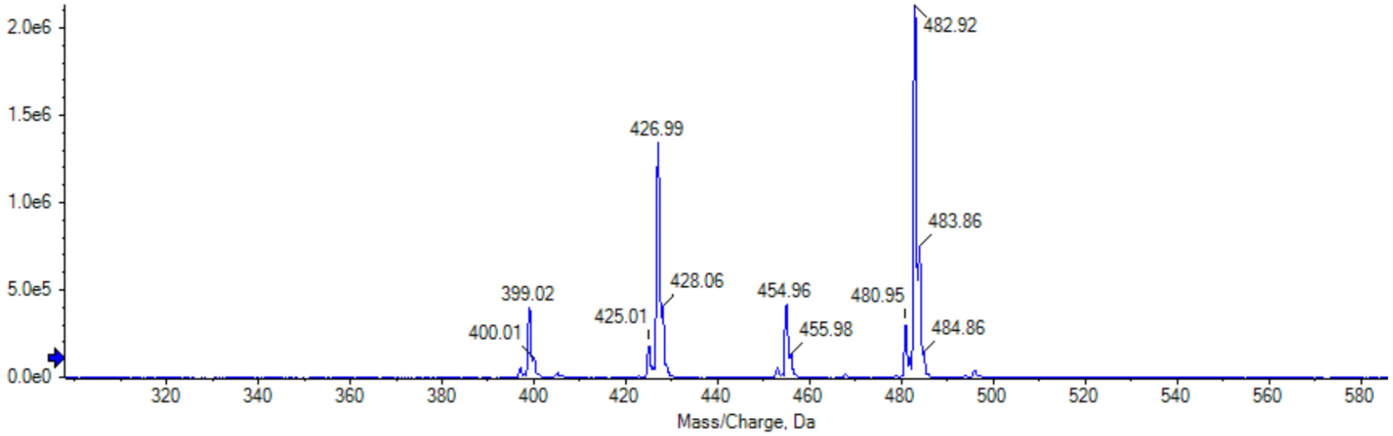

Figure S33. MS spectrum for [**2A**]$^+$ ($C_{23}H_{18}Fe_2N_2O_3$). Calcd. base peak for [**2A**]$^+$: 483.018 Da; [**2A**]$^+$−CO: 455.023 Da; [**2A**]$^+$−2CO: 427.028 Da; [**2A**]$^+$−3CO: 399.034 Da.

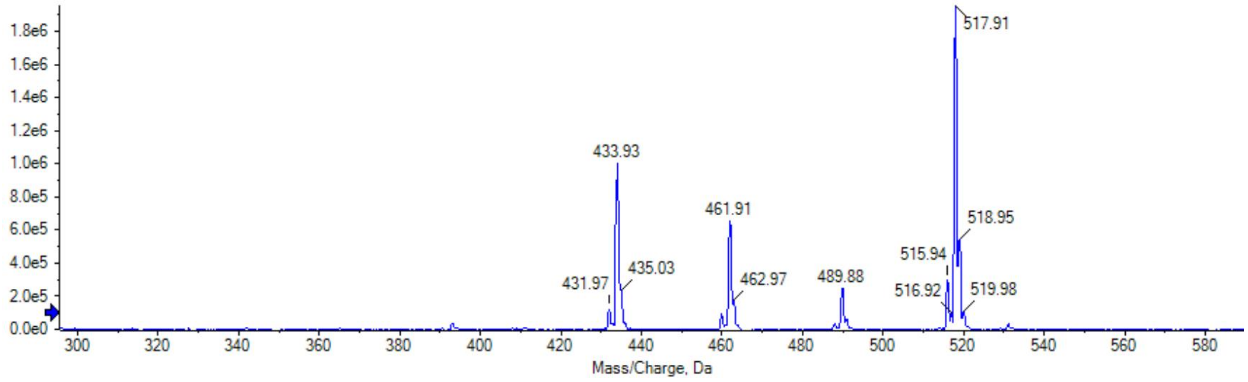

Figure S34. MS spectrum for [**2B**]$^+$ ($C_{20}H_{25}Fe_2NO_6P$). Calcd. base peak for [**2B**]$^+$: 450.054 Da; [**2B**]$^+$−CO: 422.060 Da; [**2B**]$^+$−2CO: 394.065 Da; [**2B**]$^+$−3CO: 434.036 Da.

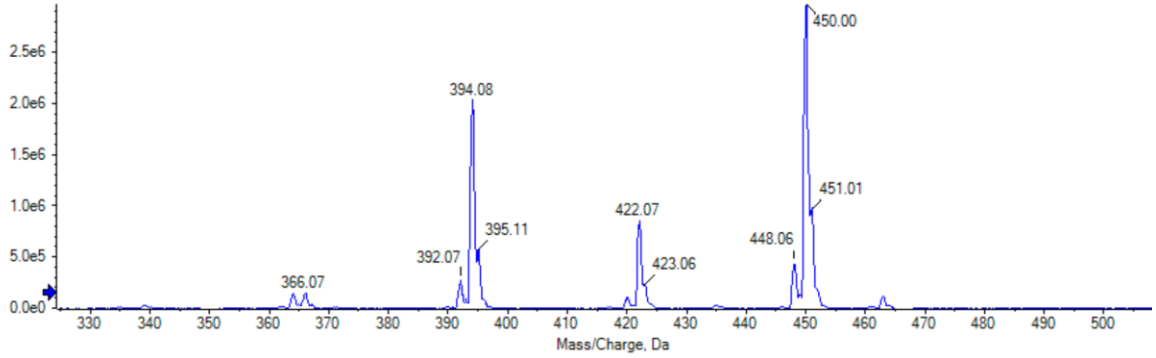

Figure S35. MS spectrum for [**2C**]$^+$ ($C_{21}H_{24}Fe_2NO_3$). Calcd. base peak for [**2C**]$^+$: 450.054 Da; [**2C**]$^+$−CO: 422.060 Da; [**2C**]$^+$−2CO: 394.065 Da.

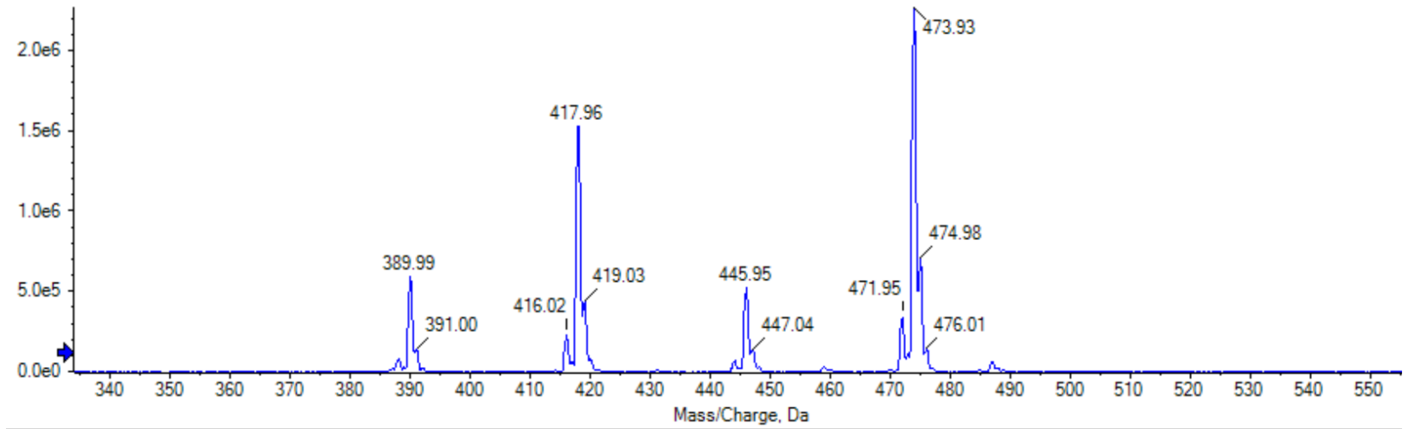

Figure S36. MS spectrum for [**2D**]$^+$ ($C_{22}H_{20}Fe_2NO_4$). Calcd. base peak for [**2D**]$^+$: 474.018 Da; [**2D**]$^+$−CO: 446.023 Da; [**2D**]$^+$−2CO: 418.029 Da; [**2D**]$^+$−3CO: 390.034.

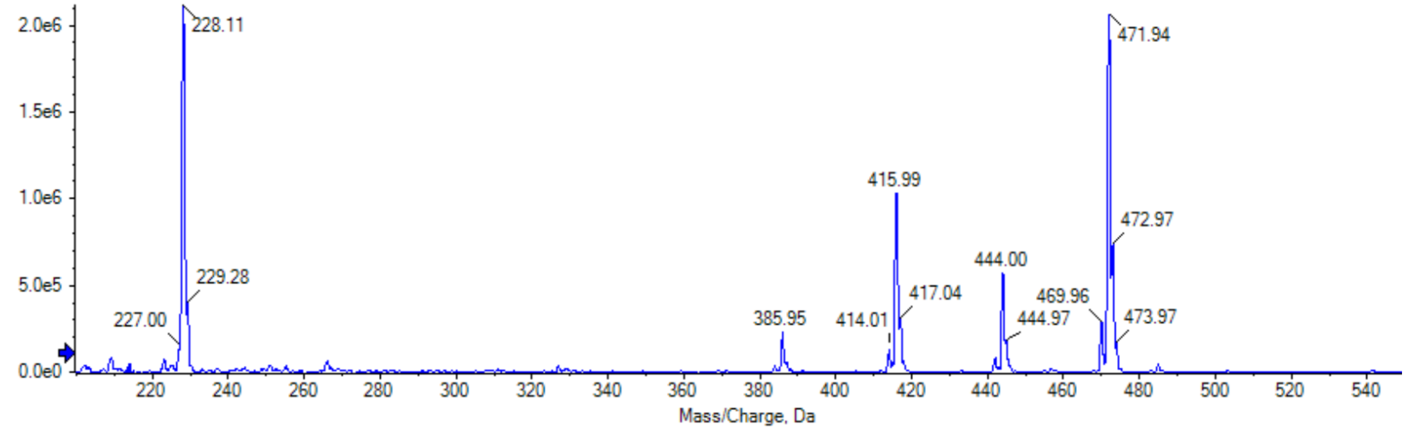

Figure S37. MS spectrum for [**2E**]$^+$ ($C_{23}H_{22}Fe_2NO_3$). Calcd. base peak for [**2E**]$^+$: 472.039 Da; [**2E**] −CO: 444.044 Da; [**2E**]$^+$−2CO: 416.049 Da; [**2E**]$^+$−3CO: 388.054 Da.

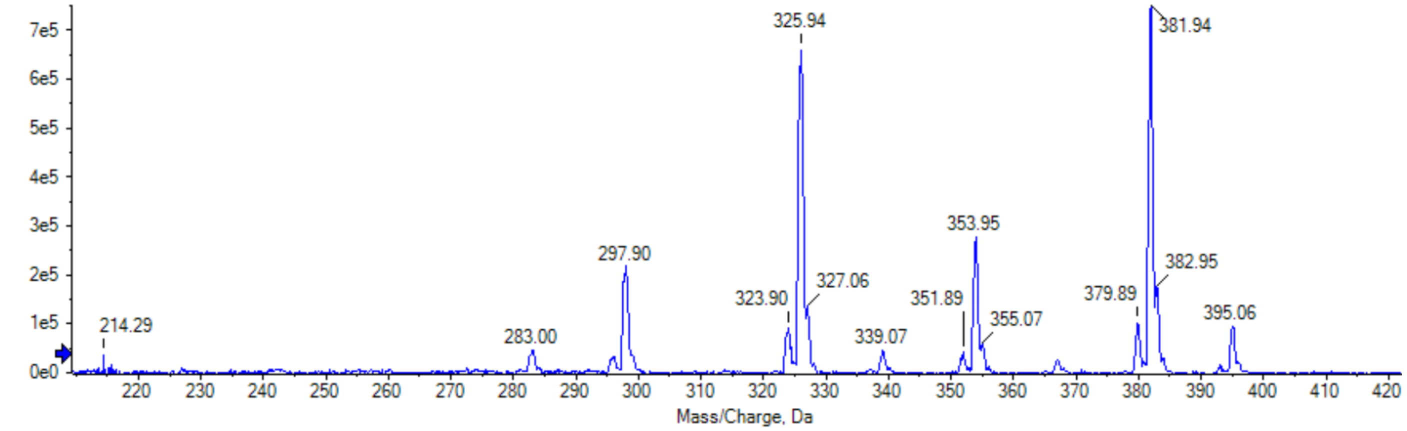

Figure S38. MS spectrum for [**2F**]$^+$ ($C_{16}H_{16}Fe_2NO_3$). Calcd. base peak for [**2F**]$^+$: 381.992 Da; [**2F**]$^+$−CO: 353.997 Da; [**2F**]$^+$−2CO: 326.002 Da; [**2F**]$^+$−3CO: 298.007 Da.

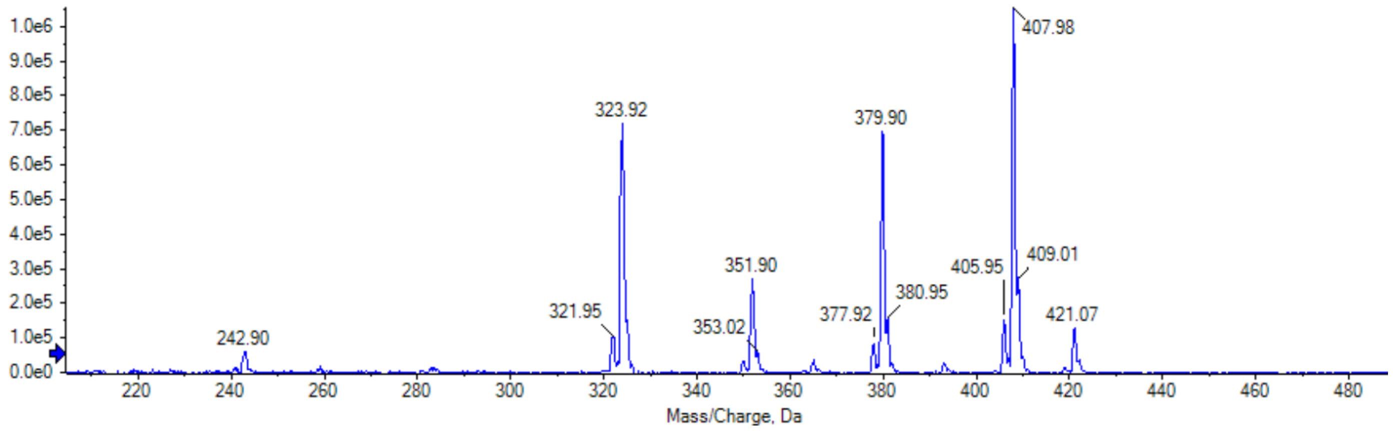

Figure S39. MS spectrum for [**3F**]$^+$ ($C_{18}H_{18}Fe_2NO_3$). Calcd. base peak for [**3F**]$^+$: 408.007 Da; [**3F**]$^+$−CO: 380.013 Da; [**3F**]$^+$−2CO: 352.018 Da; [**3F**]$^+$−3CO: 324.023 Da.

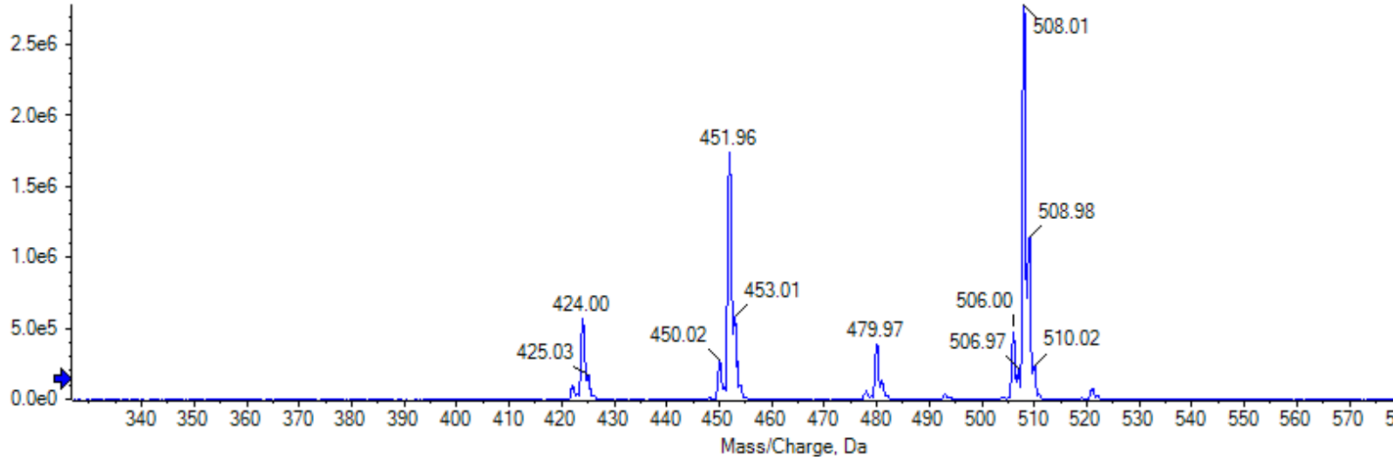

Figure S40. MS spectrum for [**4G**]$^+$ ($C_{26}H_{22}Fe_2NO_3$). Calcd. base peak for [**4G**]$^+$: 508.039 Da; [**4G**]$^+$−CO: 480.044 Da; [**4G**]$^+$−2CO: 452.049 Da; [**4G**]$^+$−3CO: 424.054 Da.

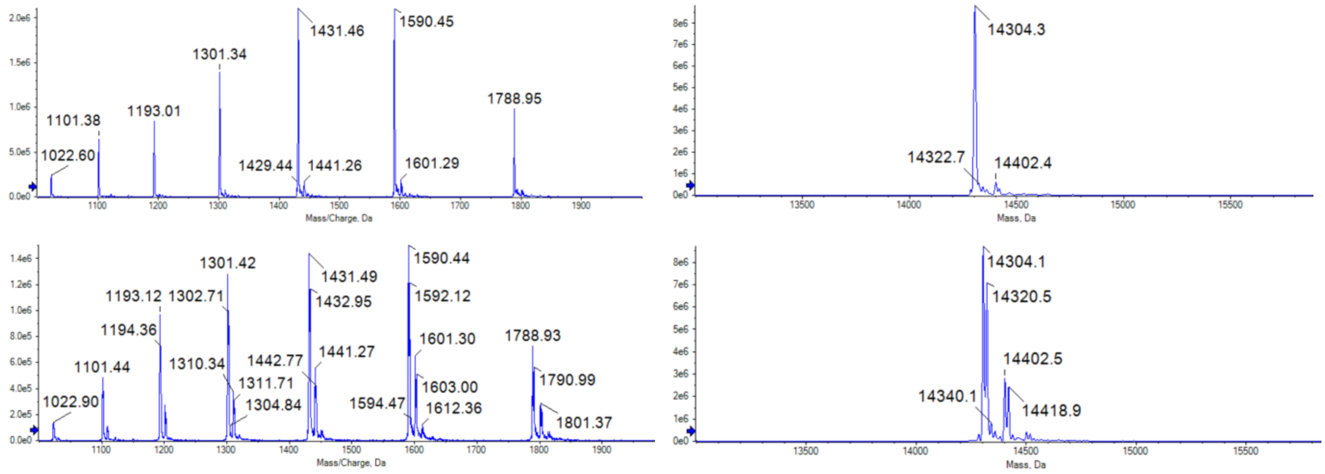

Figure S41. MS spectrum and peak reconstruction for lysozyme (M = 14304 Da) and lysozyme-CH_3 (M = 14320 Da).

7. Notes and references

1 G. Agonigi, M. Bortoluzzi, F. Marchetti, G. Pampaloni, S. Zacchini, V. Zanotti, *Eur. J. Inorg. Chem.* **2018**, 960–971, and references therein.

2 L. Busetto, F. Marchetti, S. Zacchini, V. Zanotti, *Organometallics* **2006**, *25*, 4808-4816.

3 T. Rundlöf, M. Mathiasson, S. Bekiroglu, B. Hakkarainen, T. Bowden, T. Arvidsson, *J. Pharm. Biomed. Anal.* **2010**, *52*, 645–651.

4 Calculated by the formula pD = pH* + 0.4, where pH* is the value measured for H_2O-calibrated pH-meter. a) C. C. Westcott, pH Measurements; Academic Press: New York, **1978**. b) A. K. Covington, M. Paabo, R. A. Robinson, R. G. Bates, *Anal. Chem*. **1968**, *40*, 700-706.
